# Association of DYNC1H1 gene SNP/CNV with disease susceptibility, GCs efficacy, HRQOL, anxiety, and depression in Chinese SLE patients

**DOI:** 10.1002/jcla.23892

**Published:** 2021-07-17

**Authors:** Shunwei Huang, Tingyu Zhang, Yuhua Wang, Linlin Wang, Ziye Yan, Ying Teng, Zhen Li, Qiuyue Lou, Shuang Liu, Jing Cai, Yangfan Chen, Mu Li, Hailiang Huang, Zhouzhou Xu, Yanfeng Zou

**Affiliations:** ^1^ Department of Epidemiology and Biostatistics School of Public Health Anhui Medical University Hefei China; ^2^ The Key Laboratory of Anhui Medical Autoimmune Diseases Hefei China; ^3^ Department of Rheumatology and Immunology The First Affiliated Hospital of Anhui Medical University Hefei China; ^4^ Department of Laboratory Medicine School of Public Health Anhui Medical University Hefei China; ^5^ Department of Rheumatology and Immunology The Second Affiliated Hospital of Anhui Medical University Hefei China

**Keywords:** copy number variation, glucocorticoids, single nucleotide polymorphism, systemic lupus erythematosus

## Abstract

**Background:**

Systemic lupus erythematosus is a heterogeneous autoimmune disease characterized by multi‐system injuries and overproduction of autoantibodies. There are many genetic studies on SLE, but no report has considered the relationship between cytoplasmic dynein and SLE susceptibility.

**Objectives:**

Our study intends to investigate whether DYNC1H1 gene SNP/CNV is related to SLE susceptibility, GCs efficacy, HRQOL, anxiety, and depression in Chinese SLE patients.

**Methods:**

A total of 502 cases and 544 healthy controls were recruited into the case‐control study, and 472 subjects from the case group were followed up for 12 weeks to evaluate GCs efficacy, HRQOL, anxiety, and depression. Multiplex SNaPshot technique was applied to genotype the seven SNPs of DYNC1H1, and AccuCopy^TM^ method was conducted to quantify the copy number of DYNC1H1. Anxiety and depression were evaluated using HAMA and HAMD‐24 scales, respectively. The SF‐36 scale was used to assess HRQOL.

**Results:**

The significant association between SNP rs1190606 and SLE susceptibility was displayed in the dominant model (*P_BH_* = 0.004) as well as its allele model (*P_BH_* = 0.004). We also found that SNP rs2273440 was related to photosensitization symptom in SLE patients (*P_BH_* = 0.032). In the follow‐up study, SNP rs11160668 was connected with the improvement of BP in male patients (*P_BH_* = 0.011). However, no association of DYNC1H1 gene with GCs efficacy, anxiety, and depression was found. No CNV in DYNC1H1 was detected.

**Conclusions:**

The study suggests that DYNC1H1 gene polymorphisms may have an effect on SLE susceptibility and BP improvement of HRQOL in Chinese SLE patients.

## Graphical abstract

In allele analysis, the frequency of allele G of rs1190606 in SLE patients was significantly higher than that in controls (39.14% vs 31.89%; *P* = 5.30×10^‐4^, *P_BH_* = 0.004). In genetic polymorphism analysis, rs1190606 was associated with the susceptibility of SLE (dominant model: *P_adj_* = 5.75×10^‐4^, *P_BH_* = 0.004).

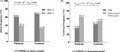

## 1 INTRODUCTION

Systemic lupus erythematosus (SLE) is an intricate autoimmune disease characterized by a broad spectrum of organ damage manifestations and flare‐remission pattern.^1^ Although the pathogenesis of SLE has not been clearly elucidated, the fact that it is a multifactorial disease involving genetic, epigenetic, and environmental factors has been identified.^2^ The report pointed out that SLE mainly harms women, whose disease risk is more than nine times that of men.^3^ In the past decade, more than 100 genetic loci related to SLE have been identified based on genome‐wide association studies (GWAS).^4^ The study on SLE genetics has attracted more and more attention from researchers.

As the most frequently used drug in SLE treatment, glucocorticoids (GCs) mainly exert their biological functions through the glucocorticoid receptor (GR). GCs are divided into endogenous and exogenous, both of which play an essential part in the pathology of SLE.^5^ Generally speaking, SLE patients can be treated effectively with GCs, whereas some unpredictable patients respond poorly to GCs therapy and even have undesirable outcomes.^6^ Therefore, it is worth noting how to identify and deal with individuals who are ineffective in GCs treatment.

Cytoplasmic dynein is a necessary molecular motor belonging to the AAA family of ATPases.^7^ Dynein is mainly responsible for facilitating the retrograde movement of numerous intracellular cargos along microtubules to ensure accurate and efficient cell activities.^8^ Dynein 1 heavy chain 1 (DYNC1H1) encodes an essential subunit, which is the core structure of dynein complex.^9^ DYNC1H1 is located on chromosome 14q32.31, which has a miRNA cluster that regulates 48 lupus susceptibility genes.^10^ Researchers have discovered that dynein can regulate the development of B cells closely related to autoimmune diseases with distinctive genetic mechanisms.^11^ The above findings seem to suggest that DYNC1H1 may be associated with SLE susceptibility. After GCs bind to GR, the formed complex must rely on dynein to drive the movement toward the nucleus.^12^ We assumed that the structural changes of dynein might have an impact on the transport of the complex, leading to glucocorticoid resistance. Previous research has also revealed that GR gene polymorphisms affect the GCs efficacy in SLE patients.^13^ Thus, we hypothesized that DYNC1H1 gene polymorphisms might make a difference to GCs efficacy in SLE. Additionally, the relationship between copy number variation (CNV) of DYNC1H1 and SLE susceptibility was also explored in this paper.

SLE is closely related to central nervous system damage, resulting in a wide range of neurologic sequels, of which anxiety and depression are the most common disorders.^14^ Possible causes of the high incidence of mental disorders in SLE patients include long‐term GCs use, psychological pressure, and recurrent symptoms.^15^ Dynein plays a vital role in the development of neurons, such as neuronal migration and neuronal regeneration.^16^ Mutations in DYNC1H1 induce dysfunction of dynein‐dependent signaling pathways, resulting in various neurological diseases.^17^ No one has yet considered linking the variations in DYNC1H1 with anxiety and depression. Meanwhile, frequent disease activity tends to worsen physical and mental health, resulting in a deterioration in SLE patients’ health‐related quality of life (HRQOL).^18^ Given previous research showing the impact of GR gene polymorphisms on HRQOL, we assumed that DYNC1H1 (the driving force for GR transport) might similarly affect HRQOL.^19^ Therefore, we sought to probe whether DYNC1H1 gene polymorphisms have an effect on mental disorders (anxiety and depression) and HRQOL in SLE patients.

## 2 MATERIALS AND METHODS

### 2.1 Subject and inclusion criteria

The research program has been approved by the ethics committee of Anhui Medical University before implementation. All selected subjects offered written informed consent. This study mainly consisted of a case‐control study aimed at probing the association between DYNC1H1 gene and SLE and a follow‐up study aimed at exploring whether DYNC1H1 gene affects the GCs efficacy, HRQOL, anxiety, and depression in SLE patients.

In the case‐control study, 502 diagnosed SLE cases were collected from the First and Second Affiliated Hospitals of Anhui Medical University, and 544 healthy controls were recruited from the healthy population identified by the physical examination departments of hospitals. Each patient was diagnosed by rheumatologists referring to the SLE diagnostic criteria published by the American College of Rheumatology in 1997. Cases with other autoimmune diseases and critical illness were not included in the case group. Moreover, the included healthy controls did not have SLE or other immune system diseases and denied a family history of SLE.

A total of 472 patients with SLE were recruited in the follow‐up study. All enrolled patients achieved a total point of 5 or higher on the SLE disease activity index (SLEDAI) at baseline. Meanwhile, participants did not accept GCs treatment within the first three months of enrollment or only obtained the minimum dose of GCs for maintenance treatment. Besides, subjects who met any of the following criteria would be eliminated from the study: (a) having severe lupus crisis; (b) patients who are allergic to hydroxychloroquine or have contraindications for GCs; (c) pregnant or lactating woman; and (d) being treated with GCs plus therapy. Of the 472 patients, a total of 19 subjects were lost to follow up. 453 patients who completed a 12‐week follow‐up would be included in the DYNC1H1 gene polymorphism and GCs efficacy, HRQOL, anxiety, depression, and improvement studies. In addition, a total of 547 individuals (262 patients and 285 controls) were recruited to the CNV study.

### 2.2 Assessment of GCs efficacy and division of groups

All patients were required to receive 12 weeks of GCs treatment, with GCs dose depending on the severity of the disease at baseline. Overall, a GCs (prednisone) treatment dose of 10mg/day ‐ 0.5mg/kg/day would be available to patients with total scores below 10, and patients whose total scores on ≥10 would receive a treatment dose of 0.5 – 1.0mg/kg/day. In the meantime, the adjustment to GCs dose required consultation with rheumatologists. Furthermore, combination therapy of hydroxychloroquine, which exerted beneficial effects in improving clinical symptoms and retarding the onset of damage, was provided to patients.^20^

Rheumatologists performed SLEDAI score assessment on all patients at baseline, 4, 8, and 12 weeks to understand their disease status. Additionally, the clinical symptoms of patients were recorded in detail. The change in SLEDAI score after 12 weeks reflected the efficacy of GCs. Patients were assigned to different groups according to the reduction of SLEDAI score and the improvement of clinical symptoms. Patients whose SLEDAI score reduced to below 5 points at 12 weeks or the diminished score (baseline ‐ 12 weeks) reached at least 5 points were divided into the GC‐sensitive groups; otherwise, patients would be classified as the GC‐insensitive group. Moreover, somebody treated with other immunosuppressors owing to poor efficacy would also be assigned to the GC‐insensitive group.

### 2.3 Assessment of HRQOL

The Medical Outcome Study Short Form 36 (SF‐36), a commonly used questionnaire for both research and clinical purposes, was adopted to monitor HRQOL of SLE patients.^21^ It contains evaluations of eight aspects (physical functions [PF], role‐physical [RP], bodily pain [BP], general health [GH], vitality [VT], social functions [SF], role‐emotional [RE], and mental health [MH]. Moreover, the total scores of physical component summary [PCS] and mental component summary [MCS] were calculated through these eight dimensions. The score range of each dimension is 0 – 100, with a higher score demonstrating higher‐level HRQOL. We measured the HRQOL of each subject separately at baseline and 12 weeks. The judgment of baseline HQORL depended on the comparison of SF‐36 total score and median: a total score greater than the median meant the good quality of life, while others meant the poor quality of life. The change in the score of each dimension after 12 weeks was viewed as an indicator to reflect the improvement of HRQOL in SLE patients.

### 2.4 Assessment of anxiety and depression

Both the Hamilton Depression Rating Scale (HAMD‐24) and the Hamilton Anxiety Rating Scale (HAMA) are currently widely adopted psychometric instruments.^22^, ^23^ The HAMD‐24 was applied to evaluate depression, and a score of 8 or above was diagnosed as depression. The HAMA was employed to assess anxiety, and a score greater than 6 was determined as anxiety. Changes in HAMA and HAMD‐24 scale scores after 12 weeks (score at baseline ‐ score at 12 weeks) reflected the improvement of patients’ anxiety and depression.

### 2.5 Tag SNPs electing and genotyping

The HapMap database was adopted to search for single nucleotide polymorphisms (SNPs) in DYCN1H1 gene representing Han‐Chinese. The linkage disequilibrium (LD) was determined by Haploview software version 4.0 to screen candidate tag SNPs. Tap SNPs with minor allele frequency (MAF) > 0.01 and threshold of *r* ^2^ > 0.8 were further investigated. Ultimately, a total of nine tag SNPs in DYCN1H1 gene (rs1004903, rs10131545, rs10132469, rs11160668, rs1190605, rs1190606, rs12161908, rs2273440, and rs3818188) were selected.

The QIAGEN DNA extraction kit (Germany) was employed to acquire genomic DNA from peripheral blood samples. DNA samples were preserved in the medical refrigerator at −80℃ until being used. SNPs genotyping was performed using the Multiplex SNaPshot technique based on ABI fluorescence‐based assay discrimination method (Applied Biosystems, Foster City, CA). The SNaPshot information of DYNC1H1 gene was presented in Supplementary Table S1.

### 2.6 Detection of DYNC1H1 copy number

The quantification of DYNC1H1 copy number was accomplished through a well‐established AccuCopy^TM^ method. AccuCopy^TM^ method is based on competitive PCR amplification, which can accurately obtain the CNV status at multiple genomic loci in the same assay.^24^ The primer information of the detected fragment was shown in Supplementary Table S2.

### 2.7 Statistical analysis

For continuous variables, arithmetic mean with standard deviation (mean (*SD*)) was employed to represent normally distributed data, while median with inter‐quartile range (median, (*P_25_*‐*P_75_*)) was applied to describe abnormally distributed variables. Categorical variables were presented as counts (percentage). In order to assess the differences between groups, Student's *t* test was adopted in the case of normally distributed data and Mann‐Whitney U test in the case of abnormally distributed data. Chi‐squared or Fisher's exact test was utilized to compare differences between qualitative variables. Similarly, Hardy‐Weinberg equilibrium (HWE) also was evaluated by chi‐squared test. We conducted univariate logistic regression to discover potential associations and presented the results in the form of odds ratios (*OR*) with 95% confidence intervals (*95% CIs*) and *P* value. To control the interference of confounding factors in the case‐control study (sex, age) and the follow‐up study (sex, age, height, weight, marital status, cigarette smoking, alcohol drinking, tea consumption, history of GCs therapy, GCs dose, and SLEDAI scores at baseline), analysis results were corrected with multivariate logistic regression.

The above analyses were performed by SPSS version 21.0 (SPSS, Inc., Chicago, IL). This study also performed haplotype analysis using SHEsis software, with the criterion that the haplotype had a minimum frequency greater than 0.03. For false‐positive error and multiple comparisons problems, the Benjamini‐Hochberg (BH) method based on the false discovery rate (FDR) was conducted to adjust *P* value. Additionally, we have further analyzed data using gender as a stratification factor. The threshold of significance was *P* < 0.05 (2‐sided) for all tests.

## 3 RESULTS

### 3.1 Case‐control study

#### 3.1.1 Characteristics and HWE analysis

The basic and clinical characteristics of cases and controls were shown in Supplementary Table S3. The case‐control study consisted of 502 patients and 544 healthy controls. There were 50 males and 452 females in the case group with an average age of 35.32 (12.22) years, while the control group had 63 males and 481 females with an average age of 35.31 (9.65) years. No statistical differences in age and gender between groups were found. Genotype frequencies of DYNC1H1 gene and HWE results were summarized in Supplementary Table S4. Since rs10131545 and rs10132469 did not meet HWE, subsequent analyses would exclude these two SNPs.

#### 3.1.2 Allele of SNPs and SLE susceptibility

**FIGURE 1:**
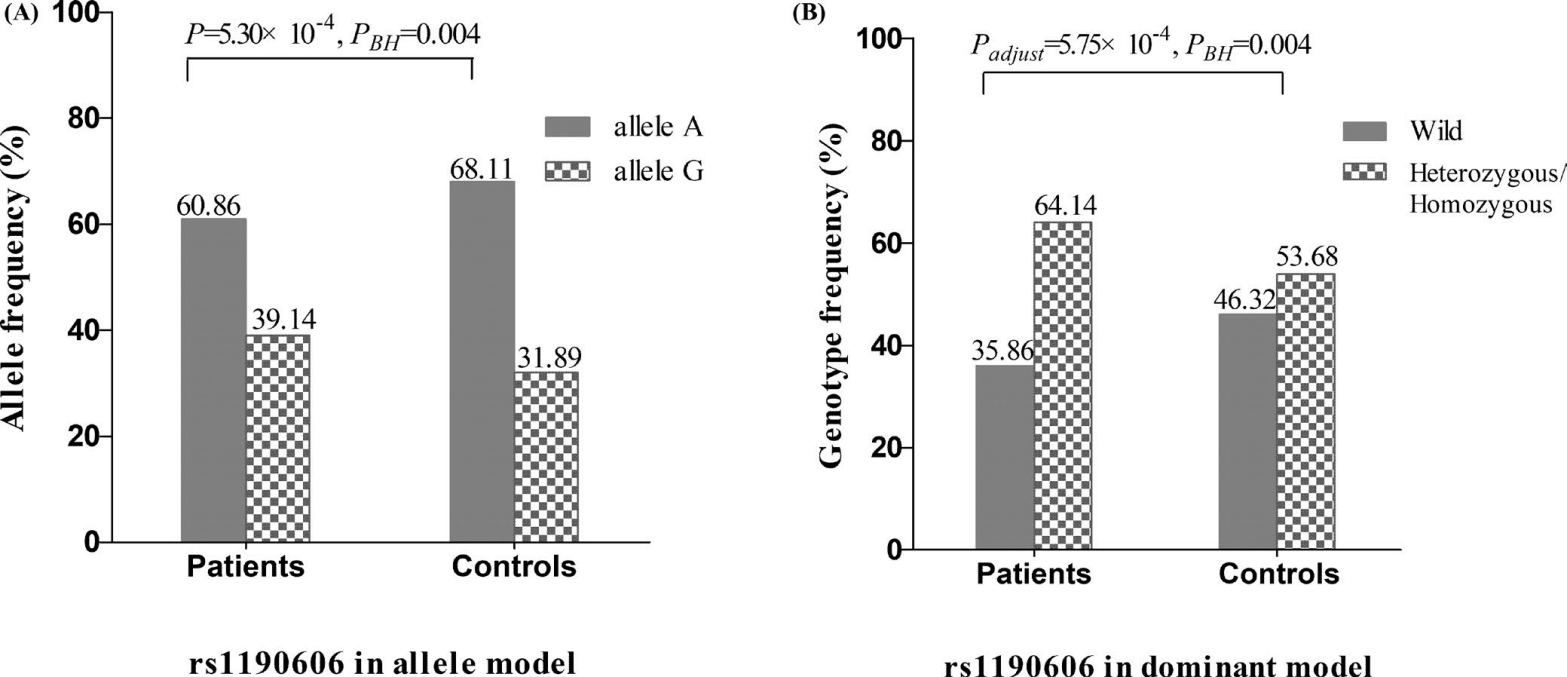
The association between rs1190606 of DYNC1H1 gene and SLE susceptibility: A, Allele distribution of rs1190606 between case and control groups; B, Genotype frequency of rs1190606 in dominant model between case and control groups

We analyzed the distribution of SNP alleles between SLE cases and controls. As shown in Table 1, compared with the control group, the frequency of three minor alleles in the case group was significantly higher, including rs1004903 (allele A, *P* = 0.039), rs1190606 (allele G, *P* = 5.30×10^−4^), and rs2273440 (allele A, *P* = 0.038). After adjusting *P* value, the result showed that the higher frequency of allele G in rs1190606 might contribute to an increased risk of SLE (*P_BH_* = 0.004) (Figure 1A). The results of gender stratification demonstrated this association was more significant in the female subgroup (*P_BH_* = 0.001) (Supplementary Tables S5 and S6).

**TABLE 1:** Comparison of different alleles of DYNC1H1 gene in patients and controls

Allele	Patients (N=502) [n(%)]	Controls (N=544) [n(%)]	*χ*^2^ value	*OR* (*95% CI*)	*P* value	*P_BH_*
rs1004903			4.255	1.234 (1.010–1.507)	**0.039**	0.091
G	739 (73.61)	843 (77.48)				
A	265 (26.39)	245 (22.52)				
rs11160668			1.091	0.898 (0.733–1.099)	0.296	0.414
G	778 (77.49)	822 (75.55)				
A	226 (22.51)	266 (24.45)				
rs1190605			3.611	1.201 (0.994–1.450)	0.057	0.100
G	692 (68.92)	791 (72.70)				
C	312 (31.08)	297 (27.30)				
rs1190606			12.006	1.374 (1.148–1.644)	**5.30×10^−4^**	**0.004**
A	611 (60.86)	741 (68.11)				
G	393 (39.14)	347 (31.89)				
rs12161908			0.114	1.068 (0.729–1.565)	0.736	0.859
G	949 (94.52)	1032 (94.85)				
C	55 (5.48)	56 (5.15)				
rs2273440			4.290	1.233 (1.011–1.503)	**0.038**	0.091
G	733 (73.01)	837 (76.93)				
A	271 (26.99)	251 (23.07)				
rs3818188			0.016	0.989 (0.833–1.174)	0.899	0.899
G	526 (52.39)	567 (52.11)				
A	478 (47.61)	521 (47.89)				

Abbreviations: *BH*, Benjamini‐Hochberg method based on the false discovery rate;*CI*, confidence interval; *OR*, odds ratio.

#### 3.1.3 DYNC1H1 gene polymorphisms and SLE susceptibility

The results of regression analyses were displayed in Table 2. In univariate logistic regression, SNP rs1190606 was associated with SLE in diverse inherited models (dominant model: *P* = 6.09×10^−4^; recessive model: *P* = 0.046). In multivariate logistic regression, the association between rs1190606 and SLE still had statistical significance (dominant model: *P*_adj_ = 5.75×10^−4^; recessive model: *P_adj_* = 0.045). By FDR correction, the statistical significance still existed in the dominant model (*P_BH_* = 0.004) (Figure 1B), suggesting that SNP rs1190606 may be a risk factor for SLE. Gender stratification analysis indicated that the association between rs1190606 and SLE in the female subgroup was in agreement with the above result (Supplementary Tables S7 and S8).

**TABLE 2:** Association between DYNC1H1 polymorphisms and susceptibility of SLE

Polymorphisms	Dominant model					Recessive model				
	Crude *OR* (*95% CI*)	Crude *P* value	Adjusted^*^ *OR* (*95% CI*)	Adjusted *P* value	*P_BH_*	Crude *OR* (*95% CI*)	Crude *P* value	Adjusted^*^ *OR* (*95% CI*)	Adjusted *P* value	*P_BH_*
rs1004903	1.243 (0.971–1.589)	0.084	1.248 (0.975–1.596)	0.078	0.208	1.472 (0.908–2.386)	0.117	1.473 (0.908–2.389)	0.116	0.203
rs11160668	0.921 (0.720–1.178)	0.510	0.926 (0.724–1.186)	0.543	0.760	0.688 (0.396–1.192)	0.182	0.695 (0.400–1.207)	0.196	0.274
rs1190605	1.201 (0.942–1.531)	0.139	1.206 (0.945–1.538)	0.132	0.231	1.473 (0.948–2.288)	0.085	1.469 (0.945–2.283)	0.087	0.203
rs1190606	1.544 (1.204–1.979)	6.09×10^−4^	1.547 (1.207–1.984)	**5.75×10^−4^**	**0.004**	1.465 (1.006–2.131)	0.046	1.469 (1.009–2.139)	**0.045**	0.203
rs12161908	1.027 (0.689–1.533)	0.895	1.023 (0.685–1.526)	0.912	0.960	3.265 (0.338–31.486)	0.306	3.223 (0.333–31.201)	0.312	0.364
rs2273440	1.234 (0.965–1.578)	0.094	1.238 (0.968–1.583)	0.089	0.208	1.477 (0.930–2.347)	0.099	1.476 (0.929–2.346)	0.100	0.203
rs3818188	1.014 (0.773–1.331)	0.918	1.007 (0.767–1.322)	0.960	0.960	0.954 (0.715–1.273)	0.748	0.945 (0.707–1.262)	0.701	0.701

#### 3.1.4 Haplotype of DYNC1H1 gene and SLE susceptibility

Haplotype analysis retained only six combinations with the lowest frequency >0.03 among 38 haplotypes (Supplementary Table S9). The result demonstrated that there were statistical differences in the distribution of haplotypes GAGAGGG and GAGGGGG between SLE patients and controls (*P* = 0.017 and *P* = 0.024). These associations disappeared after correcting *P* value by FDR method. However, the analysis of the female subgroup indicated that haplotypes GAGAGGG and GAGGGGG still had associations with SLE susceptibility (*P_BH_*, 0.015 and 0.006, respectively) (Table 3), whereas no connection was found in males (Supplementary Table S10). These associations further supported the hypothesis that DYNC1H1 gene polymorphisms may make a difference to SLE susceptibility.

**TABLE 3:** Association between haplotypes of DYNC1H1 and susceptibility of SLE (female)

Haplotypes	Cases (frequencies)	Controls (frequencies)	*χ²* value	Fisher's *P* value	Pearson's *P* value	*OR* (*95% CI*)	*P_BH_*
A G C G G A G	204.27 (0.226)	193.47 (0.201)	2.352	0.125	0.125	1.191 (0.952–1.489)	0.250
G A G A G G G	142.96 (0.158)	203.68 (0.212)	7.848	0.005	**0.005**	0.712 (0.562–0.904)	**0.015**
G A G G G G G	33.82 (0.037)	12.73 (0.013)	11.662	0.001	**0.001**	2.959 (1.542–5.676)	**0.006**
G G C A C G G	42.12 (0.047)	44.97 (0.047)	0.005	0.942	0.942	1.016 (0.660–1.564)	0.942
G G G A G G A	339.31 (0.375)	385.64 (0.401)	0.680	0.410	0.410	0.923 (0.763–1.116)	0.492
G G G G G G A	81.50 (0.090)	74.62 (0.078)	1.225	0.268	0.268	1.204 (0.866–1.673)	0.402

Note

frequency <0.03 in both control and case has been dropped.

Abbreviations: *BH*, Benjamini‐Hochberg method based on the false discovery rate;*CI*, confidence interval; *OR*, odds ratio.

#### 3.1.5 DYNC1H1 gene polymorphisms and clinical manifestations

The relationship between genotype distribution in diverse inherited models and clinical manifestations of SLE patients was presented in Supplementary Table S11. The rs2273440 of DYNC1H1 may be related to photosensitization symptom (dominant model: *OR* = 0.576, *95%CI* = 0.398–0.834, *P_BH_* = 0.040). Further gender stratification analysis did not find this association, but the association of rs1190605 with photosensitization symptom in the female subgroup was found (*P_BH_* = 0.040) (Supplementary Tables S12 and S13).

### 3.2 Follow‐up study

#### 3.2.1 Demographic features and genotype frequencies

472 SLE patients recruited from the case group were required to meet the 12‐week follow‐up. 453 subjects reached the follow‐up endpoint, whereas 19 subjects failed. Subsequently, 453 patients were assigned to the GCs‐sensitive group (261 cases) and the GCs‐insensitive group (192 cases) based on GCs efficacy. Comparisons of demographic features showed that no significance of difference existed between groups (Supplementary Table S14). Genotype frequencies and HWE analysis of seven SNPs (rs1004903, rs11160668, rs1190605, rs1190606, rs12161908, rs2273440, and rs3818188) were listed in Supplementary Table S15. Due to failure to pass HWE, SNP rs2273440 would be excluded from the analysis.

#### 3.2.2 Allele of SNPs and GCs efficacy

Differences in allelic distribution between the GC‐sensitive group and the GC‐insensitive group were shown in Supplementary Table S16. No association between SNP alleles and GCs efficacy was observed. Similarly, gender stratification results found no statistical differences (Supplementary Tables S17 and S18).

#### 3.2.3 DYNC1H1 gene polymorphisms and GCs efficacy

No statistical association was observed in logistic regression analysis (Table 4). In gender stratification, a statistical association between SNP rs3818188 and GCs efficacy was observed in males (Recessive model: *P_adj_* = 0.041). However, this association disappeared after FDR correcting (Supplementary Tables S19 and S20).

**TABLE 4:** Association between DYNC1H1 polymorphisms and GCs efficacy in followed up patients

Polymorphisms	Dominant model					Recessive model				
	Crude *OR* (*95% CI*)	Crude *P* value	Adjusted^*^ *OR* (*95% CI*)	Adjusted *P* value	*P_BH_*	Crude *OR* (*95% CI*)	Crude *P* value	Adjusted^*^ *OR* (*95% CI*)	Adjusted *P* value	*P_BH_*
rs1004903	1.024 (0.704–1.491)	0.900	0.997 (0.680–1.464)	0.989	0.991	0.686 (0.341–1.378)	0.289	0.677 (0.332–1.381)	0.283	0.733
rs11160668	0.973 (0.665–1.424)	0.890	0.949 (0.643–1.400)	0.793	0.991	0.615 (0.230–1.649)	0.334	0.673 (0.246–1.842)	0.441	0.733
rs1190605	1.003 (0.691–1.457)	0.986	0.976 (0.666–1.432)	0.902	0.991	0.914 (0.494–1.690)	0.774	0.896 (0.478–1.680)	0.733	0.733
rs1190606	0.992 (0.674–1.459)	0.966	0.969 (0.653–1.438)	0.877	0.991	1.067 (0.626–1.819)	0.811	1.114 (0.643–1.928)	0.701	0.733
rs12161908	0.993 (0.532–1.851)	0.982	1.004 (0.532–1.894)	0.991	0.991	2.737 (0.246–30.403)	0.412	2.852 (0.252–32.229)	0.397	0.733
rs3818188	1.175 (0.767–1.800)	0.458	1.144 (0.740–1.768)	0.546	0.991	1.033 (0.667–1.601)	0.884	1.119 (0.712–1.760)	0.625	0.733

#### 3.2.4 Haplotype of DYNC1H1 gene and GCs efficacy

Haplotype analysis indicated that SNP haplotype combinations had no relationship with GCs efficacy. Gender stratification analysis was also consistent with this conclusion. Details were presented in Supplementary Tables S21‐S23.

#### 3.2.5 Analysis of the improvement of anxiety, depression, and HRQOL

The results of Mann‐Whitney *U* test indicated that there was no link between DYNC1H1 gene polymorphism and the improvement of anxiety and depression (Supplementary Table S24). Supplementary Table S25 demonstrated that rs11160668 was correlated with improvement in RE (*P* = 0.016) and RP (*P*= 0.047), rs1190605 was correlated with improvement in PF (*P* = 0.030) and PCS (*P* = 0.046), and rs1190606 had statistical connection with improvement in SF (*P* = 0.034). After FDR detection, these associations disappeared. However, stratification results found that rs11160668 was correlated with improvement of BP in males (Wild vs. Heterozygous/Homozygous: 16.00 (6.50–27.00) vs. 0 (0–0), *P_BH_* =0.011) (Supplementary Table S26). In addition, logistic regressions were used to analyze whether DYNC1H1 gene has an effect on anxiety, depression, and HRQOL in 453 baseline patients. Regrettably, no statistical association was observed (Supplementary Tables S27‐S29). The same was true for gender stratification (details were not shown here).

### 3.3 CNV analysis of DYNC1H1 gene

For 547 samples, the copy number of DYNC1H1 gene fragment was tested. The original data were displayed in Figure 1, 2. After testing, the copy number of DYNC1H1 in all samples was 2, and no copy number variation was discovered. Thus, no more analysis was performed.

**FIGURE 2:**
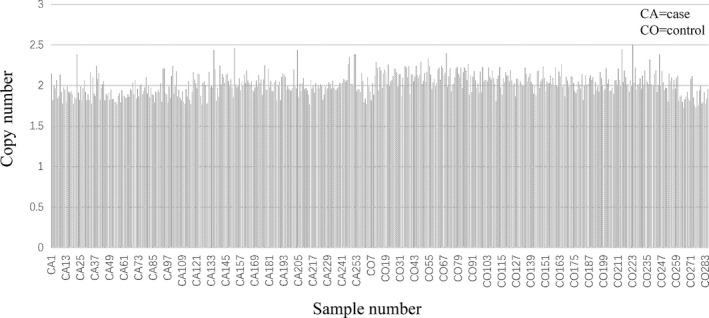
Copy number variation of DYNC1H1 gene between case and control groups

## 4 DISCUSSION

Our findings indicated that DYNC1H1 gene rs1190606 polymorphism was related to SLE. Meanwhile, we observed that the haplotypes GAGAGGG and GAGGGGG defined by seven SNPs were associated with SLE. Besides, results demonstrated that SNP rs11160668 was associated with BP improvement of male patients, and SNP rs1190605 was correlated with photosensitization symptom in female patients. Nevertheless, the effect of DYNC1H1 gene polymorphism on GCs efficacy and mental disorders (anxiety and depression) was not discovered. Similarly, no copy number variation of DYNC1H1 gene was detected. To date, most researches regarding DYNC1H1 mutation have focused on neurodegenerative diseases. This is the first study to link DYNC1H1 gene polymorphisms with SLE susceptibility. We presented the following opinions to illustrate our findings.

The pathological similarities between autoimmune diseases and cancer, such as abnormal cell proliferation and tissue microenvironment disorders, have been recognized for decades.^25^ Due to the function of dynein in maintaining the cell cycle, its role in cancer has been extensively studied. Somatic mutations of DYNC1H1 have been detected in several cancers, including intraductal papillary mucinous neoplasm, pancreatic neuroendocrine neoplasm, and glioblastoma multiforme.^26^, ^27^ DYNC1H1 has been recognized as a driver gene for colorectal cancer to progress to stage‐II.^28^ Huang et al. stated that, compared with cholecystitis and normal gallbladder tissues, the expression of DNYC1H1 in human primary gallbladder cancer (PGC) was markedly down‐regulated. Their results indicated that DYNC1H1 was a potential diagnostic biomarker for PGC.^29^ The reported data showed that DYNC1H1 was overexpressed in gastric cancer cell lines resistant to 5‐fluorouracil, paclitaxel, and cisplatin, suggesting that DYNC1H1 may be used as a biomarker for predicting chemotherapy efficacy in gastric cancer.^30^

Moreover, some studies have revealed that dynein seems to play a key role in certain autoimmune diseases. It has been shown that dynein gene may be associated with rheumatoid arthritis, an autoimmune illness partially similar to the manifestations and pathology of SLE.^31^, ^32^ Kreutzer et al. reported that axonopathy in the multiple sclerosis (MS) model was related to impaired axonal transport and was accompanied by the down‐regulation of the axonal transport‐related protein DYNC1H1, implicating DYNC1H1 may act as an MS‐responsive protein.^33^ It was confirmed that type 1 diabetes could cause changes in the distribution of dynein in the retina and lead to retinal dysfunction, suggesting that abnormal expression of retinal dynein could be used as an early indicator of retinopathy.^34^ On the other hand, B and T lymphocytes are essential for controlling inflammation response and maintaining immune homeostasis. Their development and other cellular activities appear to be inseparable from the participation of dynein. Dynein‐driven T‐cell receptor microclusters (TCR‐MCs) control T‐cell activation by forming the central supramolecular activation cluster (cSMAC). Akiko et al. confirmed that reduction of dynein heavy chains could suppress TCR‐MC translocation and cSMAC formation, thereby regulating T‐cell activation.^35^ Meanwhile, it has been proved that dynein is required for the movement of B‐cell receptor microclusters and antigen gathering during B‐cell activation.^36^ Thus, it is reasonable to speculate that mutations in DYNC1H1 gene may interfere with its biological function and affect the activation of B and T cells, thereby changing the susceptibility to SLE. The above evidence seems to support our findings regarding the relationship between DYNC1H1 gene polymorphisms and SLE susceptibility. Remarkably, we observed a gender difference in the association of DYNC1H1 gene with SLE, which may be related to the high risk of SLE in women, or to the limited male cases included in this study.

As the drug of choice for SLE treatment, GCs mainly rely on GR to exert anti‐inflammatory and immunomodulatory functions. Mediated by transport‐related proteins, GR forms the GR‐Hsp90‐Hsp70‐immunophilin complex, which then is transported retrogradely with the assistance of cytoplasmic dynein.^37^, ^38^ Daghestani et al. screened GR nuclear translocation inhibitors based on changes in cytoplasmic dynein ATPase activity. They believed that dynein‐mediated transport inhibitors mainly targeted dynein and other transport‐related proteins.^39^ Although previous studies confirmed that HSP90AA1 gene involved in GR transport affects GC efficacy, our observations have not suggested the influence of DYNC1H1 gene on GCs efficacy.^40^ This may be because individual differences in response to GCs are caused by a combination of various mechanisms, which requires more exploration.

HRQOL is adopted for the individual's self‐assessment of disease and treatment, including multiple dimensions of psychological, physical, and social functions.^41^ Studies have shown that the quality of life of SLE patients is not only worse than that of normal people, but even patients with general chronic diseases.^42^ Paying attention to the HRQOL of SLE patients is of great significance for evaluating SLE treatment and predicting mortality.^43^ Dynein interacts directly or indirectly with the PPiase domain of FKBP52.^44^ The fact that FKBP5 gene polymorphisms have an influence on HRQOL of patients with SLE has been confirmed.^45^ Considering the interaction between dynein and FKBP5, it is reasonable to speculate that DYNC1H1 gene may have a similar effect on HRQOL. In this study, we observed an association between rs11160668 of DYNC1H1 gene and BP improvement of HRQOL. However, this association only existed in male patients, which may be due to the small sample size caused by gender stratification. More studies with larger sample sizes are warranted to affirm the conclusion.

Variations in DYNC1H1 gene have been repeatedly reported to be involved in the pathogenesis of neurodegenerative diseases.^46^ Additionally, several animal behavioral studies suggested that dynein genes influenced anxiety and depression behaviors in mice. Banks et al. stated that the mutation of DYNC1LI1 gene altered the development of mice neurons, which made the mutant mice present stronger anxiety.^47^ The experiment conducted by Donner et al. found that the high expression of DYNLL2 haplotype enhanced the susceptibility of mice to generalized anxiety disorder.^48^ Bakhtiarzadeh et al. believed that the stress‐induced depression in rats might be related to the decreased expression of dynein gene.^49^ Nevertheless, this investigation found no association of DYNC1H1 with anxiety and depression in patients with SLE. We hypothesized that the difference in conclusions might be partly due to the differences in cross‐species studies, looking forward to more human‐based investigations.

This paper had several inevitable limitations. Firstly, 19 patients withdrew from the follow‐up, which unavoidably affected the analysis results to a certain extent. Secondly, all patients would be given hydroxychloroquine for combination therapy. Because of the slow onset of the drug (3 to 6 months), it would have an impact on the evaluation of GCs efficacy. Thirdly, all subjects were from the Han population in Anhui Province, and the influence of race and geographic environment was not taken into consideration. Fourthly, the limited number of male cases might have an effect on gender stratification analysis. Fifthly, the CNV was not detected in this study, which might be the result of a small sample size. At last, there were still some potential confounding factors that had not been considered, leading to biased results.

## 5 CONCLUSION

Overall, our study for the first time demonstrates that DYNC1H1 (rs1190606) gene polymorphism may be connected with SLE susceptibility. Meanwhile, DYNC1H1 (rs11160668) gene may have an association with the improvement of HRQOL in SLE patients. Nevertheless, this paper failed to find the connection of DYNC1H1 gene with GCs efficacy and mental disorders (anxiety and depression). What's more, no CNV of DYNC1H1 gene was found in our observations. This study is expected to provide a new direction for additional research on the pathogenesis of SLE. It is necessary to conduct more studies covering more SNPs with more representative samples for further exploration.

## CONFLICT OF INTERESTS

The authors declared no potential conflicts of interest with respect to the research, authorship, and/or publication of this article.

## Supporting information

Table S1‐S29

## Data Availability

The data that support the findings of this study are available from the corresponding author upon reasonable request.

## References

[1] Ocampo‐Piraquive V, Nieto‐Aristizábal I, Cañas CA, Tobón GJ. Mortality in systemic lupus erythematosus: causes, predictors and interventions. Expert Rev Clin Immunol. 2018;14(12):1043‐1053. 30338717 10.1080/1744666X.2018.1538789

[2] Constantin MM, Nita IE, Olteanu R, et al. Significance and impact of dietary factors on systemic lupus erythematosus pathogenesis. Exp Ther Med. 2019;17(2):1085‐1090. 30679978 10.3892/etm.2018.6986 PMC6327661

[3] Nusbaum JS, Mirza I, Shum J, et al. Sex Differences in Systemic Lupus Erythematosus: Epidemiology, Clinical Considerations, and Disease Pathogenesis. Mayo Clin Proc. 2020;95(2):384‐394. 32029091 10.1016/j.mayocp.2019.09.012

[4] Ghodke‐Puranik Y, Imgruet M, Dorschner JM, et al. Novel genetic associations with interferon in systemic lupus erythematosus identified by replication and fine‐mapping of trait‐stratified genome‐wide screen. Cytokine. 2020;132:154631. 30685201 10.1016/j.cyto.2018.12.014 PMC7723062

[5] Bazsó A, Szappanos Á, Patócs A, Poór G, Shoenfeld Y, Kiss E. The importance of glucocorticoid receptors in systemic lupus erythaematosus. A systematic review. Autoimmun Rev. 2015;14(4):349‐351. 25526806 10.1016/j.autrev.2014.12.007

[6] Gao HY, Wang Q, Yu XW, et al. Molecular mechanisms of glucocorticoid resistance in systemic lupus erythematosus: A review. Life Sci. 2018;209:383‐387. 30125579 10.1016/j.lfs.2018.08.038

[7] Bhabha G, Johnson GT, Schroeder CM, Vale RD. How Dynein Moves Along Microtubules. Trends Biochem Sci. 2016;41(1):94‐105. 26678005 10.1016/j.tibs.2015.11.004 PMC4706479

[8] Dwivedi D, Sharma M. Multiple Roles, Multiple Adaptors: Dynein During Cell Cycle. Adv Exp Med Biol. 2018;1112:13‐30. 30637687 10.1007/978-981-13-3065-0_2

[9] Strickland AV, Schabhüttl M, Offenbacher H, et al. Mutation screen reveals novel variants and expands the phenotypes associated with DYNC1H1. J Neurol. 2015;262(9):2124‐2134. 26100331 10.1007/s00415-015-7727-2 PMC4573829

[10] Vinuesa CG, Rigby RJ, Yu D. Logic and extent of miRNA‐mediated control of autoimmune gene expression. Int Rev Immunol. 2009;28(3–4):112‐138. 19811318 10.1080/08830180902934909

[11] King A, Li LL, Wong DM, et al. Dynein light chain regulates adaptive and innate B cell development by distinctive genetic mechanisms. PLoS Genet. 2017;13(9):e1007010. 28922373 10.1371/journal.pgen.1007010 PMC5619840

[12] Harrell JM, Murphy PJ, Morishima Y, et al. Evidence for glucocorticoid receptor transport on microtubules by dynein. J Biol Chem. 2004;279(52):54647‐54654. 15485845 10.1074/jbc.M406863200

[13] Zou YF, Xu JH, Wang F, et al. Association study of glucocorticoid receptor genetic polymorphisms with efficacy of glucocorticoids in systemic lupus erythematosus: a prospective cohort study. Autoimmunity. 2013;46(8):531‐536. 24151836 10.3109/08916934.2013.830714

[14] Kheirandish M, Faezi ST, Paragomi P, et al. Prevalence and severity of depression and anxiety in patients with systemic lupus erythematosus: An epidemiologic study in Iranian patients. Mod Rheumatol. 2015;25(3):405‐409. 25295916 10.3109/14397595.2014.962241

[15] Nery FG, Borba EF, Viana VS, et al. Prevalence of depressive and anxiety disorders in systemic lupus erythematosus and their association with anti‐ribosomal P antibodies. Prog Neuropsychopharmacol Biol Psychiatry. 2008;32(3):695‐700. 18077068 10.1016/j.pnpbp.2007.11.014

[16] Marzo MG, Griswold JM, Ruff KM, Buchmeier RE, Fees CP, Markus SM. Molecular basis for dyneinopathies reveals insight into dynein regulation and dysfunction. Elife. 2019;8:e47246. 31364990 10.7554/eLife.47246 PMC6733598

[17] Chen XJ, Xu H, Cooper HM, Liu YB. Cytoplasmic dynein: a key player in neurodegenerative and neurodevelopmental diseases. Sci China Life Sci. 2014;57(4):372‐377. 24664850 10.1007/s11427-014-4639-9

[18] Elera‐Fitzcarrald C, Fuentes A, González LA, Burgos PI, Alarcón GS, Ugarte‐Gil MF. Factors affecting quality of life in patients with systemic lupus erythematosus: important considerations and potential interventions. Expert Rev Clin Immunol. 2018;14(11):915‐931. 30266076 10.1080/1744666X.2018.1529566

[19] Zou YF, Xu JH, Pan FM, et al. Glucocorticoid receptor genetic polymorphisms is associated with improvement of health‐related quality of life in Chinese population with systemic lupus erythematosus. Clin Rheumatol. 2015;34(9):1537‐1544. 26255187 10.1007/s10067-015-3027-6

[20] Ponticelli C, Moroni G. Hydroxychloroquine in systemic lupus erythematosus (SLE). Expert Opin Drug Saf. 2017;16(3):411‐419. 27927040 10.1080/14740338.2017.1269168

[21] Lins L, Carvalho FM. SF‐36 total score as a single measure of health‐related quality of life: Scoping review. SAGE Open Med. 2016;4:2050312116671725. 27757230 10.1177/2050312116671725 PMC5052926

[22] Moberg PJ, Lazarus LW, Mesholam RI, et al. Comparison of the standard and structured interview guide for the Hamilton Depression Rating Scale in depressed geriatric inpatients. Am J Geriatr Psychiatry. 2001;9(1):35‐40. 11156750

[23] Clark DB, Donovan JE. Reliability and validity of the Hamilton Anxiety Rating Scale in an adolescent sample. J Am Acad Child Adolesc Psychiatry. 1994;33(3):354‐360. 8169180 10.1097/00004583-199403000-00009

[24] Du RQ, Lu CC, Jiang ZW, et al. Efficient typing of copy number variations in a segmental duplication‐mediated rearrangement hotspot using multiplex competitive amplification. J Hum Genet. 2012;57(8):545‐551. 22673690 10.1038/jhg.2012.66

[25] Eck SM, Blackburn JS, Schmucker AC, Burrage PS, Brinckerhoff CE. Matrix metalloproteinase and G protein coupled receptors: co‐conspirators in the pathogenesis of autoimmune disease and cancer. J Autoimmun. 2009;33(3–4):214‐221. 19800199 10.1016/j.jaut.2009.09.011 PMC2783549

[26] Jiao YC, Shi CJ, Edil BH, et al. DAXX/ATRX, MEN1, and mTOR pathway genes are frequently altered in pancreatic neuroendocrine tumors. Science. 2011;331(6021):1199‐1203. 21252315 10.1126/science.1200609 PMC3144496

[27] Furukawa T, Kuboki Y, Tanji E, et al. Whole‐exome sequencing uncovers frequent GNAS mutations in intraductal papillary mucinous neoplasms of the pancreas. Sci Rep. 2011;1:161. 22355676 10.1038/srep00161 PMC3240977

[28] Palaniappan A, Ramar K, Ramalingam S. Computational Identification of Novel Stage‐Specific Biomarkers in Colorectal Cancer Progression. PLoS One. 2016;11(5):e0156665. 27243824 10.1371/journal.pone.0156665 PMC4887059

[29] Huang HL, Yao HS, Wang Y, Wang WJ, Hu ZQ, Jin KZ. Proteomic identification of tumor biomarkers associated with primary gallbladder cancer. World J Gastroenterol. 2014;20(18):5511‐5518. 24833881 10.3748/wjg.v20.i18.5511 PMC4017066

[30] Huang H, Han Y, Wu J, Tian ZH, Qu LK, Shou CC. Establishment of drug resistant cell line of MGC‐803 and analysis of differential secretome. Beijing Da Xue Xue Bao Yi Xue Ban. 2014;46(2):183‐189. 24743803

[31] Kraja AT, Corbett J, Ping A, et al. Rheumatoid arthritis, item response theory, Blom transformation, and mixed models. BMC Proc. 2007;1(S1):S116. 18466457 10.1186/1753-6561-1-s1-s116 PMC2367565

[32] Kahlenberg JM, Kaplan MJ. Mechanisms of premature atherosclerosis in rheumatoid arthritis and lupus. Annu Rev Med. 2013;64:249‐263. 23020882 10.1146/annurev-med-060911-090007 PMC4198172

[33] Kreutzer M, Seehusen F, Kreutzer R, et al. Axonopathy is associated with complex axonal transport defects in a model of multiple sclerosis. Brain Pathol. 2012;22(4):454‐471. 21988534 10.1111/j.1750-3639.2011.00541.x PMC8092950

[34] Baptista FI, Pinto MJ, Elvas F, Martins T, Almeida RD, Ambrósio AF. Diabetes induces changes in KIF1A, KIF5B and dynein distribution in the rat retina: implications for axonal transport. Exp Eye Res. 2014;127:91‐103. 25064602 10.1016/j.exer.2014.07.011

[35] Hashimoto‐Tane A, Yokosuka T, Sakata‐Sogawa K, et al. Dynein‐driven transport of T cell receptor microclusters regulates immune synapse formation and T cell activation. Immunity. 2011;34(6):919‐931. 21703543 10.1016/j.immuni.2011.05.012

[36] Schnyder T, Castello A, Feest C, et al. B cell receptor‐mediated antigen gathering requires ubiquitin ligase Cbl and adaptors Grb2 and Dok‐3 to recruit dynein to the signaling microcluster. Immunity. 2011;34(6):905‐918. 21703542 10.1016/j.immuni.2011.06.001

[37] Galigniana MD, Echeverría PC, Erlejman AG, Piwien‐Pilipuk G. Role of molecular chaperones and TPR‐domain proteins in the cytoplasmic transport of steroid receptors and their passage through the nuclear pore. Nucleus. 2010;1(4):299‐308. 21113270 10.4161/nucl.1.4.11743 PMC2990191

[38] Wochnik GM, Rüegg J, Abel GA, Schmidt U, Holsboer F, Rein T. FK506‐binding proteins 51 and 52 differentially regulate dynein interaction and nuclear translocation of the glucocorticoid receptor in mammalian cells. J Biol Chem. 2005;280(6):4609‐4616. 15591061 10.1074/jbc.M407498200

[39] Daghestani HN, Zhu G, Johnston PA, Shinde SN, Brodsky JL, Day BW. Characterization of inhibitors of glucocorticoid receptor nuclear translocation: a model of cytoplasmic dynein‐mediated cargo transport. Assay Drug Dev Technol. 2012;10(1):46‐60. 21919741 10.1089/adt.2010.0367 PMC3277738

[40] Zou YF, Xu JH, Gu YY, et al. Single nucleotide polymorphisms of HSP90AA1 gene influence response of SLE patients to glucocorticoids treatment. Springerplus. 2016;5:222. 27026916 10.1186/s40064-016-1911-4 PMC4771663

[41] Olesińska M, Saletra A. Quality of life in systemic lupus erythematosus and its measurement. Reumatologia. 2018;56(1):45‐54. 29686443 10.5114/reum.2018.74750 PMC5911658

[42] Fung W, Lim LSH, Tomlinson G, et al. Joint trajectories of disease activity, and physical and mental health‐related quality of life in an inception lupus cohort. Rheumatology (Oxford). 2020;59(10):3032‐3041. 32191334 10.1093/rheumatology/keaa091

[43] Manzano BR, da Silva Santos PS, Bariquelo MH, Merlini NRG, Honório HM, Rubira CMF. A case‐control study of oral diseases and quality of life in individuals with rheumatoid arthritis and systemic lupus erythematosus. Clin Oral Investig. 2021;25(4):2081‐2092. 10.1007/s00784-020-03518-8 32803443

[44] Galigniana MD, Harrell JM, Murphy PJM, et al. Binding of hsp90‐associated immunophilins to cytoplasmic dynein: direct binding and in vivo evidence that the peptidylprolyl isomerase domain is a dynein interaction domain. Biochemistry. 2002;41(46):13602‐13610. 12427021 10.1021/bi020399z

[45] Lou QY, Li Z, Teng Y, et al. Associations of FKBP4 and FKBP5 gene polymorphisms with disease susceptibility, glucocorticoid efficacy, anxiety, depression, and health‐related quality of life in systemic lupus erythematosus patients. Clin Rheumatol. 2021;40(1):167‐179. 32557257 10.1007/s10067-020-05195-0

[46] Hoang HT, Schlager MA, Carter AP, Bullock SL. DYNC1H1 mutations associated with neurological diseases compromise processivity of dynein‐dynactin‐cargo adaptor complexes. Proc Natl Acad Sci U S A. 2017;114(9):e1597‐1606. 28196890 10.1073/pnas.1620141114 PMC5338514

[47] Banks GT, Haas MA, Line S, et al. Behavioral and other phenotypes in a cytoplasmic Dynein light intermediate chain 1 mutant mouse. J Neurosci. 2011;31(14):5483‐5494. 21471385 10.1523/JNEUROSCI.5244-10.2011 PMC3096546

[48] Donner J, Pirkola S, Silander K, et al. An association analysis of murine anxiety genes in humans implicates novel candidate genes for anxiety disorders. Biol Psychiatry. 2008;64(8):672‐680. 18639233 10.1016/j.biopsych.2008.06.002 PMC2682432

[49] Bakhtiarzadeh F, Nahavandi A, Goudarzi M, Shirvalilou S, Rakhshan K, Niknazar S. Axonal transport proteins and depressive like behavior, following Chronic Unpredictable Mild Stress in male rat. Physiol Behav. 2018;194:9‐14. 29698729 10.1016/j.physbeh.2018.04.029

