# Supplementary material for: Association of DYNC1H1 gene SNP/CNV with disease susceptibility, GCs efficacy, HRQOL, anxiety, and depression in Chinese SLE patients
Source: J Clin Lab Anal. 2021 Jul 17;35(8):e23892. doi: 10.1002/jcla.23892 (PMC8373356; doi:10.1002/jcla.23892)
Supplement: Supplementary file 1 — Table S1‐S29 [file JCLA-35-e23892-s001.docx]

| **Table S1** SNaPshot information of DYNC1H1 gene | | | | | | | |
| --- | --- | --- | --- | --- | --- | --- | --- |
| **SNPs** | **Gene** | **Alleles** | **Location** | **Reference mRNA** | **SNP Property** | **Length** | **PCRPrimer** |
| rs1004903 | DYNC1H1 | G/A | Chr14:102515015 | NM_001376.4 | Intron74 | 333 | rs1004903F/R |
| rs10131545 | DYNC1H1 | T/C | Chr14:102459725 | NM_001376.4 | Intron11 | 317 | rs10131545F/R |
| rs10132469 | DYNC1H1 | G/A | Chr14:102456391 | NM_001376.4 | Intron10 | 293 | rs10132469F/R |
| rs11160668 | DYNC1H1 | G/A | Chr14:102513086 | NM_001376.4 | Intron72 | 237 | rs11160668F/R |
| rs1190605 | DYNC1H1 | G/C | Chr14:102509953 | NM_001376.4 | Intron69 | 300 | rs1190605F/R |
| rs1190606 | DYNC1H1 | G/A | Chr14:102510428 | NM_001376.4 | Intron70 | 244 | rs1190606F/R |
| rs12161908 | DYNC1H1 | G/C | Chr14:102454910 | NM_001376.4 | Iintron9 | 336 | rs12161908F/R |
| rs2273440 | DYNC1H1 | G/A | Chr14:102510874 | NM_001376.4 | Intron71 | 302 | rs2273440F/R |
| rs3818188 | DYNC1H1 | G/A | Chr14:102446161 | NM_001376.4 | Exon4 | 321 | rs3818188F/R |
| *SNaPshot*, Multiplex *SNaPshot* technique | | | | | | | |

| **Table S2** Primer information of the detected fragment for DYNC1H1 gene | | | | |
| --- | --- | --- | --- | --- |
| **Chromosome** | **Location**  **(GRCh38 database)^*^** | **Amplification length**  **(sample, competitive)^#^** | **Primer binding region 1** | **Primer binding region 2** |
| chr14 | 101986209-101986342 | 134 (+0, -2) | ATGAAGCGGGTGGAAGATGTC | ACCTTCCTTGCCCAGTCATCA |
| ^*^GRCH38 reference primary assembly; ^#^Sample: sample DNA, competitive: competitive DNA | | | | |

| **Table S3** Basic and clinical characteristics of SLE patients and controls | | | | | |
| --- | --- | --- | --- | --- | --- |
| **Parameter** | **Patients (N=502)** | **Controls (N=544)** | **t / χ^2^ value** | ***P* value** |  |
| Age, mean (SD) | 35.32 (12.22) | 35.31 (9.65) | 0.017 | 0.986 |  |
| Sex, no. (%) |  |  | 0.712 | 0.399 |  |
| Female | 452 (90.00) | 481 (88.40) |  |  |  |
| Male | 50 (10.00) | 63 (11.60) |  |  |  |
| Clinical features, no. (%) |  |  |  |  |  |
| Lupus nephritis | 150 (29.88) |  |  |  |  |
| [Photosensitization](D:/%E6%96%87%E4%BB%B6/Dict/8.5.0.0/resultui/html/index.html#/javascript:;) | 192 (38.25) |  |  |  |  |
| [Arthritis](D:/%E6%96%87%E4%BB%B6/Dict/8.5.0.0/resultui/html/index.html#/javascript:;) | 230 (45.82) |  |  |  |  |
| Erythema | 259 (51.59) |  |  |  |  |
| [Alopecia](D:/%E6%96%87%E4%BB%B6/Dict/8.5.0.0/resultui/html/index.html#/javascript:;) | 159 (31.67) |  |  |  |  |
| [Canker](D:/%E6%96%87%E4%BB%B6/Dict/8.5.0.0/resultui/html/index.html#/javascript:;) [sore](D:/%E6%96%87%E4%BB%B6/Dict/8.5.0.0/resultui/html/index.html#/javascript:;) | 93 (18.53) |  |  |  |  |
| P[leurisy](D:/%E6%96%87%E4%BB%B6/Dict/8.5.0.0/resultui/html/index.html#/javascript:;) | 42 (8.37) |  |  |  |  |
| Hematologic disorder | 261 (51.99) |  |  |  |  |
| Anti-dsDNA | 215 (42.83) |  |  |  |  |
| Low complement | 283 (56.37) |  |  |  |  |
| *SD*, standard deviation | | | | | |

| **Table S4** Genotype frequency distribution of DYNC1H1 gene in patients and controls | | | | | | |
| --- | --- | --- | --- | --- | --- | --- |
| **Polymorphisms**  **(minor allele)** | **Genotype [n (%)]** | | |  | **HWE** | |
|  | **Wild** | **Heterozygous** | **Homozygous mutants** |  | **χ² value** | ***P* value** |
| rs1004903 (A) |  |  |  |  |  |  |
| Patients | 278 (55.38) | 183 (36.45) | 41 (8.17) |  | 1.917 | 0.166 |
| Controls | 330 (60.66) | 183 (33.64) | 31 (6.70) |  | 0.704 | 0.401 |
| rs10131545 (C) |  |  |  |  |  |  |
| Patients | 501 (99.80) | 1 (0.20) | 0 (0.00) |  | 0.001 | 0.982 |
| Controls | 544 (100.00) | 0 (0.00) | 0 (0.00) |  | – | – |
| rs10132469 (A) |  |  |  |  |  |  |
| Patients | 502 (100.00) | 0 (0.00) | 0 (0.00) |  | – | – |
| Controls | 544 (100.00) | 0 (0.00) | 0 (0.00) |  | – | – |
| rs11160668 (A) |  |  |  |  |  |  |
| Patients | 298 (59.36) | 182 (36.25) | 22 (4.38) |  | 0.773 | 0.379 |
| Controls | 312 (57.35) | 198 (36.40) | 34 (6.25) |  | 0.119 | 0.731 |
| rs1190605 (C) |  |  |  |  |  |  |
| Patients | 240 (47.81) | 212 (42.23) | 50 (9.96) |  | 0.101 | 0.751 |
| Controls | 285 (52.39) | 221 (40.63) | 38 (6.99) |  | 0.300 | 0.584 |
| rs1190606(G) |  |  |  |  |  |  |
| Patients | 180 (35.86) | 251 (50.00) | 71 (14.14) |  | 1.229 | 0.268 |
| Controls | 252 (46.32) | 237 (43.57) | 55 (10.11) |  | 0.004 | 0.947 |
| rs12161908(C) |  |  |  |  |  |  |
| Patients | 450 (89.64) | 49 (9.76) | 3 (0.60) |  | 1.657 | 0.198 |
| Controls | 489 (89.89) | 54 (9.93) | 1 (0.18) |  | 0.150 | 0.698 |
| rs2273440(A) |  |  |  |  |  |  |
| Patients | 276 (54.98) | 181 (36.06) | 45 (8.96) |  | 3.642 | 0.056 |
| Controls | 327 (60.11) | 183 (33.64) | 34 (6.25) |  | 1.487 | 0.223 |
| rs3818188(A) |  |  |  |  |  |  |
| Patients | 137 (27.29) | 252 (50.20) | 113 (22.51) |  | 0.020 | 0.888 |
| Controls | 150 (27.57) | 267 (49.08) | 127 (23.35) |  | 0.150 | 0.698 |
| *HWE*, Hardy-Weinberg equilibrium | | | | | | |

| **Table S5** Comparison of different alleles of DYNC1H1 gene in patients and controls (male) | | | | | | |
| --- | --- | --- | --- | --- | --- | --- |
| **Allele** | **Patients (N=50)**  **[n (%)]** | **Controls (N=63)**  **[n (%)]** | ***χ*^2^ value** | ***OR* (*95% CI*)** | ***P* value** | ***P_BH_*** |
| rs1004903 |  |  | 0.824 | 1.313 (0.729-2.368) | 0.364 | 0.900 |
| G | 70 (70.00) | 95 (75.40) |  |  |  |  |
| A | 30 (30.00) | 31 (24.60) |  |  |  |  |
| rs11160668 |  |  | 0.036 | 0.946 (0.532-1.682) | 0.850 | 0.986 |
| G | 71 (71.00) | 88 (69.84) |  |  |  |  |
| A | 29 (29.00) | 38 (30.16) |  |  |  |  |
| rs1190605 |  |  | 1.018 | 1.339 (0.759-2.365) | 0.313 | 0.900 |
| G | 66 (66.00) | 91 (72.22) |  |  |  |  |
| C | 34 (34.00) | 35 (27.78) |  |  |  |  |
| rs1190606 |  |  | 0.002 | 0.987 (0.573-1.700) | 0.963 | 0.986 |
| A | 63 (63.00) | 79 (62.70) |  |  |  |  |
| G | 37 (37.00) | 47 (37.30) |  |  |  |  |
| rs12161908 |  |  | 3.07×10^-4^ | 1.009 (0.383-2.659) | 0.986 | 0.986 |
| G | 92 (92.00) | 116 (92.06) |  |  |  |  |
| C | 8 (8.00) | 10 (7.94) |  |  |  |  |
| rs2273440 |  |  | 0.553 | 1.252 (0.692-2.263) | 0.457 | 0.900 |
| G | 71 (71.00) | 95 (75.40) |  |  |  |  |
| A | 29 (29.00) | 31 (24.60) |  |  |  |  |
| rs3818188 |  |  | 0.426 | 0.836 (0.488-1.433) | 0.514 | 0.900 |
| G | 63 (63.00) | 74 (58.73) |  |  |  |  |
| A | 37 (37.00) | 52 (41.27) |  |  |  |  |
| *OR*, odds ratio; *CI*, confidence interval; *BH*, Benjamini-Hochberg method based on the false discovery rate | | | | | | |

| **Table S6** Comparison of different alleles of DYNC1H1 gene in patients and controls (female) | | | | | | |
| --- | --- | --- | --- | --- | --- | --- |
| **Allele** | **Patients (N=452)**  **[n (%)]** | **Controls (N=481)**  **[n (%)]** | ***χ*^2^ value** | ***OR* (*95% CI*)** | ***P* value** | ***P_BH_*** |
| rs1004903 |  |  | 3.587 | 1.228 (0.993-1.519) | 0.058 | 0.135 |
| G | 669 (74.00) | 748 (77.75) |  |  |  |  |
| A | 235 (26.00) | 214 (22.25) |  |  |  |  |
| rs11160668 |  |  | 0.965 | 0.897 (0.722-1.114) | 0.326 | 0.456 |
| G | 707 (78.21) | 734 (76.30) |  |  |  |  |
| A | 197 (21.79) | 228 (23.70) |  |  |  |  |
| rs1190605 |  |  | 2.804 | 1.186 (0.971-1.450) | 0.094 | 0.165 |
| G | 626 (69.25) | 700 (72.77) |  |  |  |  |
| C | 278 (30.75) | 262 (27.23) |  |  |  |  |
| rs1190606 |  |  | 13.731 | 1.434 (1.185-1.735) | **2.11×10^-4^** | **0.001** |
| A | 548 (60.62) | 662 (68.81) |  |  |  |  |
| G | 356 (39.38) | 300 (31.19) |  |  |  |  |
| rs12161908 |  |  | 0.104 | 1.068 (0.716-1.592) | 0.747 | 0.872 |
| G | 853 (94.36) | 911 (94.70) |  |  |  |  |
| C | 51 (5.64) | 51 (5.30) |  |  |  |  |
| rs2273440 |  |  | 3.807 | 1.233 (0.999-1.522) | 0.051 | 0.135 |
| G | 662 (73.23) | 742 (77.13) |  |  |  |  |
| A | 242 (26.77) | 220 (22.87) |  |  |  |  |
| rs3818188 |  |  | 1.75×10^-4^ | 1.001 (0.835-1.201) | 0.989 | 0.989 |
| G | 463 (51.22) | 493 (51.25) |  |  |  |  |
| A | 441 (48.78) | 469 (48.75) |  |  |  |  |
| *OR*, odds ratio; *CI*, confidence interval; *BH*, Benjamini-Hochberg method based on the false discovery rate | | | | | | |

| **Table S7** Association between DYNC1H1 polymorphisms and susceptibility of SLE (male) | | | | | | | | | | | |
| --- | --- | --- | --- | --- | --- | --- | --- | --- | --- | --- | --- |
| **Polymorphisms** | **Dominant model** | | | | |  | **Recessive model** | | | | |
|  | **Crude**  ***OR* (*95% CI*)** | **Crude**  ***P* value** | **Adjusted^*^**  ***OR* (*95% CI*)** | **Adjusted**  ***P* value** | ***P_BH_*** |  | **Crude**  ***OR* (*95% CI*)** | **Crude**  ***P* value** | **Adjusted^*^**  ***OR* (*95% CI*)** | **Adjusted**  ***P* value** | ***P_BH_*** |
| rs1004903 | 1.671 (0.790-3.533) | 0.179 | 1.728 (0.805-3.706) | 0.160 | 0.502 |  | 0.740 (0.168-3.260) | 0.691 | 0.831 (0.185-3.722) | 0.808 | 0.809 |
| rs11160668 | 0.969 (0.461-2.035) | 0.933 | 0.932 (0.438-1.983) | 0.854 | 0.958 |  | 0.826 (0.220-3.103) | 0.777 | 0.848 (0.223-3.224) | 0.809 | 0.809 |
| rs1190605 | 1.795 (0.843-3.820) | 0.129 | 1.915 (0.884-4.150) | 0.100 | 0.502 |  | 0.740 (0.168-3.260) | 0.691 | 0.831 (0.185-3.722) | 0.808 | 0.809 |
| rs1190606 | 0.923 (0.431-1.975) | 0.837 | 1.021 (0.468-2.226) | 0.958 | 0.958 |  | 1.119 (0.376-3.329) | 0.840 | 1.197 (0.394-3.632) | 0.751 | 0.809 |
| rs12161908 | 1.009 (0.256-3.972) | 0.990 | 1.085 (0.270-4.366) | 0.908 | 0.958 |  | − | − | − | − | 0.809 |
| rs2273440 | 1.542 (0.730-3.257) | 0.257 | 1.621 (0.755-3.479) | 0.215 | 0.502 |  | 0.740 (0.168-3.260) | 0.691 | 0.831 (0.185-3.722) | 0.808 | 0.809 |
| rs3818188 | 1.116 (0.513-2.430) | 0.782 | 1.101 (0.499-2.426) | 0.812 | 0.958 |  | 0.370 (0.111-1.227) | 0.104 | 0.340 (0.100-1.151) | 0.083 | 0.498 |
| *OR*, odds ratio; *CI*, confidence interval; *BH*, Benjamini-Hochberg method based on the false discovery rate;  **^*^**Adjusted for age | | | | | | | | | | | |

| **Table S8** Association between DYNC1H1 polymorphisms and susceptibility of SLE (female) | | | | | | | | | | | |
| --- | --- | --- | --- | --- | --- | --- | --- | --- | --- | --- | --- |
| **Polymorphisms** | **Dominant model** | | | | |  | **Recessive model** | | | | |
|  | **Crude**  ***OR* (*95% CI*)** | **Crude**  ***P* value** | **Adjusted^*^**  ***OR* (*95% CI*)** | **Adjusted**  ***P* value** | ***P_BH_*** |  | **Crude**  ***OR* (*95% CI*)** | **Crude**  ***P* value** | **Adjusted^*^**  ***OR* (*95% CI*)** | **Adjusted**  ***P* value** | ***P_BH_*** |
| rs1004903 | 1.204 (0.927-1.563) | 0.163 | 1.204 (0.927-1.563) | 0.164 | 0.383 |  | 1.606 (0.959-2.692) | 0.072 | 1.621 (0.966-2.718) | 0.067 | 0.117 |
| rs11160668 | 0.921 (0.709-1.197) | 0.539 | 0.919 (0.708-1.194) | 0.528 | 0.739 |  | 0.671 (0.366-1.231) | 0.197 | 0.668 (0.364-1.225) | 0.192 | 0.269 |
| rs1190605 | 1.150 (0.890-1.487) | 0.286 | 1.147 (0.887-1.483) | 0.295 | 0.516 |  | 1.575 (0.990-2.508) | 0.055 | 1.588 (0.997-2.531) | 0.051 | 0.117 |
| rs1190606 | 1.645 (1.264-2.140) | 0.001 | 1.648 (1.266-2.144) | **0.001** | **0.007** |  | 1.523 (1.020-2.274) | 0.040 | 1.516 (1.015-2.264) | **0.042** | 0.117 |
| rs12161908 | 1.024 (0.674-1.557) | 0.911 | 1.026 (0.675-1.559) | 0.905 | 0.951 |  | 3.207 (0.332-30.945) | 0.314 | 3.066 (0.317-29.696) | 0.333 | 0.389 |
| rs2273440 | 1.205 (0.929-1.563) | 0.160 | 1.205 (0.929-1.564) | 0.160 | 0.383 |  | 1.597 (0.976-2.611) | 0.062 | 1.613 (0.986-2.640) | 0.057 | 0.117 |
| rs3818188 | 0.993 (0.742-1.328) | 0.960 | 0.991 (0.741-1.326) | 0.951 | 0.951 |  | 1.011 (0.749-1.366) | 0.941 | 1.016 (0.752-1.372) | 0.919 | 0.919 |
| *OR*, odds ratio; *CI*, confidence interval; *BH*, Benjamini-Hochberg method based on the false discovery rate;  **^*^**Adjusted for age | | | | | | | | | | | |

| **Table S9** Association between haplotypes of DYNC1H1gene and susceptibility of SLE | | | | | | | |
| --- | --- | --- | --- | --- | --- | --- | --- |
| **Haplotypes** | **Cases (frequencies)** | **Controls (frequencies)** | ***χ²* value** | **Fisher’s *P* value** | **Pearson’s *P* value** | ***OR* (*95% CI*)** | ***P_BH_*** |
| A G C G G A G | 232.36 (0.231) | 223.47 (0.205) | 2.878 | 0.090 | 0.090 | 1.199 (0.972-1.478) | 0.180 |
| G A G A G G G | 170.13 (0.169) | 233.18 (0.214) | 5.727 | **0.017** | **0.017** | 0.764 (0.613-0.953) | 0.072 |
| G A G G G G G | 35.30 (0.035) | 21.42 (0.020) | 5.089 | **0.024** | **0.024** | 1.854 (1.076-3.195) | 0.072 |
| G G C A C G G | 46.12 (0.046) | 50.18 (0.046) | 0.006 | 0.936 | 0.936 | 1.017 (0.675-1.532) | 0.936 |
| G G G A G G A | 370.10 (0.369) | 429.76 (0.395) | 0.805 | 0.370 | 0.370 | 0.921 (0.769-1.102) | 0.444 |
| G G G G G G A | 86.97 (0.087) | 82.27 (0.076) | 1.125 | 0.289 | 0.289 | 1.186 (0.865-1.625) | 0.434 |
| *OR*, odds ratio; *CI*, confidence interval; *BH*, Benjamini-Hochberg method based on the false discovery rate;  frequency < 0.03 in both control & case has been dropped | | | | | | | |

| **Table S10** Association between haplotypes of DYNC1H1 and susceptibility of SLE (male) | | | | | | | |
| --- | --- | --- | --- | --- | --- | --- | --- |
| **Haplotypes** | **Cases (frequencies)** | **Controls (frequencies)** | ***χ²* value** | **Fisher’s *P* value** | **Pearson’s *P* value** | ***OR* (*95% CI*)** | ***P_BH_*** |
| A G C G G A G | 27.99 (0.280) | 30.00 (0.238) | 0.734 | 0.392 | 0.392 | 1.300 (0.712-2.374) | 0.822 |
| G A G A G G G | 26.57 (0.266) | 29.00 (0.230) | 0.568 | 0.451 | 0.451 | 1.264 (0.687-2.327) | 0.822 |
| G A G G G G G | 1.44 (0.014) | 9.00 (0.071) | 3.924 | **0.048** | **0.048** | 0.196 (0.033-1.163) | 0.286 |
| G G C A C G G | 3.17 (0.032) | 5.00 (0.040) | 0.075 | 0.784 | 0.784 | 0.819 (0.195-3.431) | 0.822 |
| G G G A G G A | 32.25 (0.322) | 45.00 (0.357) | 0.145 | 0.703 | 0.703 | 0.897 (0.513-1.569) | 0.822 |
| G G G G G G A | 4.73 (0.047) | 7.00 (0.056) | 0.050 | 0.822 | 0.822 | 0.872 (0.263-2.889) | 0.822 |
| *OR*, odds ratio; *CI*, confidence interval; *BH*, Benjamini-Hochberg method based on the false discovery rate;  frequency < 0.03 in both control & case has been dropped. | | | | | | | |

| **Table S11** Association between DYNC1H1 polymorphisms and the clinical manifestations of SLE patients | | | | | | | | | | | |
| --- | --- | --- | --- | --- | --- | --- | --- | --- | --- | --- | --- |
| **Polymorphisms** | **Dominant model** | | | | |  | **Recessive model** | | | | |
|  | **Crude**  ***OR* (*95% CI*)** | **Crude**  ***P* value** | **Adjusted^*^**  ***OR* (*95% CI*)** | **Adjusted *P* value** | ***P_BH_*** |  | **Crude**  ***OR* (*95% CI*)** | **Crude**  ***P* value** | **Adjusted^*^**  ***OR* (*95% CI*)** | **Adjusted *P* value** | ***P_BH_*** |
| rs1004903 |  |  |  |  |  |  |  |  |  |  |  |
| Lupus nephritis | 0.826 (0.561-1.216) | 0.334 | 0.819 (0.556-1.207) | 0.313 | 0.586 |  | 0.849 (0.414-1.743) | 0.656 | 0.852 (0.415-1.751) | 0.664 | 0.727 |
| [Photosensitization](D:/%E6%96%87%E4%BB%B6/Dict/8.5.0.0/resultui/html/index.html#/javascript:;) | 0.624 (0.432-0.901) | **0.012** | 0.610 (0.421-0.883) | **0.009** | 0.009 |  | 0.567 (0.301-1.150) | 0.121 | 0.578 (0.282-1.184) | 0.134 | 0.627 |
| [Arthritis](D:/%E6%96%87%E4%BB%B6/Dict/8.5.0.0/resultui/html/index.html#/javascript:;) | 1.116 (0.784-1.588) | 0.544 | 1.131 (0.793-1.612) | 0.498 | 0.586 |  | 0.588 (0.301-1.150) | 0.121 | 0.583 (0.298-1.142) | 0.116 | 0.627 |
| [Erythema](D:/%E6%96%87%E4%BB%B6/Dict/8.5.0.0/resultui/html/index.html#/javascript:;) | 0.783 (0.550-1.114) | 0.174 | 0.788 (0.552-1.123) | 0.187 | 0.586 |  | 0.885 (0.467-1.676) | 0.707 | 0.892 (0.469-1.694) | 0.727 | 0.727 |
| [Alopecia](D:/%E6%96%87%E4%BB%B6/Dict/8.5.0.0/resultui/html/index.html#/javascript:;) | 0.896 (0.613-1.309) | 0.569 | 0.898 (0.612-1.317) | 0.583 | 0.586 |  | 0.676 (0.323-1.415) | 0.298 | 0.685 (0.325-1.441) | 0.319 | 0.648 |
| [Canker](D:/%E6%96%87%E4%BB%B6/Dict/8.5.0.0/resultui/html/index.html#/javascript:;) [sore](D:/%E6%96%87%E4%BB%B6/Dict/8.5.0.0/resultui/html/index.html#/javascript:;) | 1.270 (0.809-1.994) | 0.299 | 1.299 (0.826-2.042) | 0.258 | 0.586 |  | 0.737 (0.301-1.807) | 0.505 | 0.713 (0.290-1.755) | 0.462 | 0.660 |
| P[leurisy](D:/%E6%96%87%E4%BB%B6/Dict/8.5.0.0/resultui/html/index.html#/javascript:;) | 0.529 (0.268-1.044) | 0.066 | 0.525 (0.266-1.039) | 0.064 | 0.320 |  | 0.256 (0.034-1.911) | 0.184 | 0.259 (0.035-1.933) | 0.188 | 0.627 |
| Hematologic disorder | 1.085 (0.763-1.544) | 0.649 | 1.104 (0.775-1.572) | 0.585 | 0.586 |  | 1.334 (0.698-2.550) | 0.383 | 1.310 (0.684-2.508) | 0.415 | 0.660 |
| Anti-dsDNA | 1.106 (0.775-1.578) | 0.578 | 1.119 (0.784-1.599) | 0.536 | 0.586 |  | 1.168 (0.615-2.217) | 0.636 | 1.161 (0.611-2.206) | 0.649 | 0.727 |
| Low complement | 1.093 (0.766-1.559) | 0.623 | 1.105 (0.772-1.580) | 0.586 | 0.586 |  | 0.717 (0.378-1.359) | 0.308 | 0.723 (0.380-1.377) | 0.324 | 0.648 |
| rs11160668 |  |  |  |  |  |  |  |  |  |  |  |
| Lupus nephritis | 0.926 (0.626-1.368) | 0.698 | 0.918 (0.621-1.358) | 0.668 | 0.999 |  | 0.875 (0.3367-2.281) | 0.785 | 0.858 (0.328-2.244) | 0.755 | 0.839 |
| [Photosensitization](D:/%E6%96%87%E4%BB%B6/Dict/8.5.0.0/resultui/html/index.html#/javascript:;) | 1.035 (0.717-1.492) | 0.855 | 1.018 (0.704-1.470) | 0.926 | 0.999 |  | 1.652 (0.702-3.888) | 0.250 | 1.605 (0.680-3.790) | 0.280 | 0.839 |
| [Arthritis](D:/%E6%96%87%E4%BB%B6/Dict/8.5.0.0/resultui/html/index.html#/javascript:;) | 0.891 (0.623-1.275) | 0.528 | 0.902 (0.630-1.291) | 0.572 | 0.999 |  | 0.664 (0.784-1.612) | 0.366 | 0.682 (0.280-1.661) | 0.400 | 0.839 |
| [Erythema](D:/%E6%96%87%E4%BB%B6/Dict/8.5.0.0/resultui/html/index.html#/javascript:;) | 0.992 (0.694-1.416) | 0.964 | 1.000 (0.699-1.431) | 0.999 | 0.999 |  | 0.773 (0.328-1.824) | 0.557 | 0.792 (0.334-1.876) | 0.596 | 0.839 |
| [Alopecia](D:/%E6%96%87%E4%BB%B6/Dict/8.5.0.0/resultui/html/index.html#/javascript:;) | 0.838 (0.569-1.232) | 0.368 | 0.842 (0.571-1.243) | 0.388 | 0.999 |  | 1.245 (0.511-3.031) | 0.629 | 1.292 (0.525-3.175) | 0.577 | 0.839 |
| [Canker](D:/%E6%96%87%E4%BB%B6/Dict/8.5.0.0/resultui/html/index.html#/javascript:;) [sore](D:/%E6%96%87%E4%BB%B6/Dict/8.5.0.0/resultui/html/index.html#/javascript:;) | 0.723 (0.451-1.157) | 0.177 | 0.738 (0.460-1.184) | 0.208 | 0.999 |  | 0.976 (0.323-2.956) | 0.966 | 1.022 (0.336-3.109) | 0.969 | 0.969 |
| P[leurisy](D:/%E6%96%87%E4%BB%B6/Dict/8.5.0.0/resultui/html/index.html#/javascript:;) | 1.680 (0.892-3.167) | 0.109 | 1.683 (0.892-3.176) | 0.108 | 0.999 |  | 2.586 (0.833-8.028) | 0.100 | 2.609 (0.837-8.135) | 0.098 | 0.790 |
| Hematologic disorder | 0.998 (0.699-1.425) | 0.991 | 1.014 (0.709-1.451) | 0.937 | 0.999 |  | 0.513 (0.211-1.245) | 0.134 | 0.527 (0.216-1.283) | 0.158 | 0.790 |
| Anti-dsDNA | 0.923 (0.644-1.324) | 0.663 | 0.933 (0.650-1.340) | 0.708 | 0.999 |  | 1.119 (0.474-2.640) | 0.798 | 1.149 (0.485-2.719) | 0.752 | 0.839 |
| Low complement | 1.000 (0.698-1.432) | 1.000 | 1.008 (0.702-1.448) | 0.965 | 0.999 |  | 1.123 (0.471-2.678) | 0.793 | 1.159 (0.483-2.782) | 0.741 | 0.839 |
| rs1190605 |  |  |  |  |  |  |  |  |  |  |  |
| Lupus nephritis | 0.758 (0.517-1.111) | 0.155 | 0.751 (0.511-1.103) | 0.144 | 0.480 |  | 1.006 (0.532-1.904) | 0.985 | 1.014 (0.535-1.921) | 0.966 | 0.966 |
| [Photosensitization](D:/%E6%96%87%E4%BB%B6/Dict/8.5.0.0/resultui/html/index.html#/javascript:;) | 0.639 (0.445-0.918) | **0.015** | 0.618 (0.429-0.891) | **0.010** | 0.100 |  | 0.666 (0.353-1.255) | 0.209 | 0.681 (0.361-1.286) | 0.237 | 0.516 |
| [Arthritis](D:/%E6%96%87%E4%BB%B6/Dict/8.5.0.0/resultui/html/index.html#/javascript:;) | 1.136 (0.799-1.615) | 0.478 | 1.149 (0.807-1.637) | 0.440 | 0.733 |  | 0.700 (0.384-1.276) | 0.244 | 0.690 (0.378-1.260) | 0.227 | 0.516 |
| [Erythema](D:/%E6%96%87%E4%BB%B6/Dict/8.5.0.0/resultui/html/index.html#/javascript:;) | 0.903 (0.636-1.283) | 0.570 | 0.898 (0.631-1.279) | 0.551 | 0.787 |  | 1.018 (0.567-1.827) | 0.952 | 1.020 (0.567-1.835) | 0.948 | 0.966 |
| [Alopecia](D:/%E6%96%87%E4%BB%B6/Dict/8.5.0.0/resultui/html/index.html#/javascript:;) | 0.832 (0.571-1.212) | 0.339 | 0.815 (0.557-1.193) | 0.293 | 0.704 |  | 0.655 (0.333-1.291) | 0.222 | 0.657 (0.332-1.301) | 0.228 | 0.516 |
| [Canker](D:/%E6%96%87%E4%BB%B6/Dict/8.5.0.0/resultui/html/index.html#/javascript:;) [sore](D:/%E6%96%87%E4%BB%B6/Dict/8.5.0.0/resultui/html/index.html#/javascript:;) | 1.202 (0.764-1.890) | 0.426 | 1.241 (0.787-1.958) | 0.352 | 0.704 |  | 0.693 (0.301-1.593) | 0.388 | 0.667 (0.289-1.538) | 0.342 | 0.570 |
| P[leurisy](D:/%E6%96%87%E4%BB%B6/Dict/8.5.0.0/resultui/html/index.html#/javascript:;) | 0.597 (0.314-1.135) | 0.115 | 0.586 (0.307-1.118) | 0.105 | 0.480 |  | 0.429 (0.101-1.832) | 0.253 | 0.433 (0.101-1.848) | 0.258 | 0.516 |
| Hematologic disorder | 1.059 (0.746-1.503) | 0.750 | 1.085 (0.762-1.543) | 0.652 | 0.811 |  | 1.575 (0.864-2.870) | 0.138 | 1.542 (0.845-2.814) | 0.159 | 0.516 |
| Anti-dsDNA | 0.930 (0.653-1.326) | 0.690 | 0.939 (0.658-1.340) | 0.730 | 0.811 |  | 1.261 (0.702-2.265) | 0.437 | 1.248 (0.694-2.244) | 0.459 | 0.656 |
| Low complement | 0.977 (0.687-1.392) | 0.900 | 0.970 (0.679-1.385) | 0.866 | 0.866 |  | 1.076 (0.596-1.944) | 0.808 | 1.082 (0.596-1.962) | 0.796 | 0.966 |
| rs1190606 |  |  |  |  |  |  |  |  |  |  |  |
| Lupus nephritis | 0.841 (0.567-1.249) | 0.392 | 0.845 (0.569-1.255) | 0.404 | 0.971 |  | 1.147 (0.669-1.965) | 0.618 | 1.150 (0.671-1.972) | 0.611 | 0.806 |
| [Photosensitization](D:/%E6%96%87%E4%BB%B6/Dict/8.5.0.0/resultui/html/index.html#/javascript:;) | 0.829 (0.570-1.204) | 0.324 | 0.830 (0.571-1.207) | 0.330 | 0.971 |  | 0.689 (0.402-1.182) | 0.176 | 0.680 (0.396-1.168) | 0.163 | 0.714 |
| [Arthritis](D:/%E6%96%87%E4%BB%B6/Dict/8.5.0.0/resultui/html/index.html#/javascript:;) | 1.017 (0.705-1.466) | 0.930 | 1.009 (0.699-1.457) | 0.961 | 0.987 |  | 0.790 (0.475-1.315) | 0.365 | 0.786 (0.472-1.311) | 0.356 | 0.714 |
| [Erythema](D:/%E6%96%87%E4%BB%B6/Dict/8.5.0.0/resultui/html/index.html#/javascript:;) | 0.867 (0.601-1.249) | 0.442 | 0.855 (0.592-1.235) | 0.404 | 0.971 |  | 0.959 (0.581-1.585) | 0.871 | 0.942 (0.569-1.561) | 0.818 | 0.818 |
| [Alopecia](D:/%E6%96%87%E4%BB%B6/Dict/8.5.0.0/resultui/html/index.html#/javascript:;) | 1.129 (0.761-1.676) | 0.547 | 1.111 (0.746-1.654) | 0.604 | 0.971 |  | 0.759 (0.433-1.333) | 0.338 | 0.730 (0.414-1.288) | 0.277 | 0.714 |
| [Canker](D:/%E6%96%87%E4%BB%B6/Dict/8.5.0.0/resultui/html/index.html#/javascript:;) [sore](D:/%E6%96%87%E4%BB%B6/Dict/8.5.0.0/resultui/html/index.html#/javascript:;) | 0.910 (0.571-1.450) | 0.692 | 0.904 (0.567-1.442) | 0.672 | 0.971 |  | 0.879 (0.451-1.711) | 0.704 | 0.887 (0.455-1.730) | 0.725 | 0.806 |
| P[leurisy](D:/%E6%96%87%E4%BB%B6/Dict/8.5.0.0/resultui/html/index.html#/javascript:;) | 1.129 (0.578-2.205) | 0.722 | 1.122 (0.574-2.193) | 0.736 | 0.971 |  | 0.807 (0.306-2.127) | 0.664 | 0.793 (0.300-2.096) | 0.641 | 0.806 |
| Hematologic disorder | 0.952 (0.661-1.372) | 0.792 | 0.948 (0.658-1.368) | 0.777 | 0.971 |  | 0.882 (0.534-1.457) | 0.623 | 0.889 (0.537-1.471) | 0.646 | 0.806 |
| Anti-dsDNA | 1.003 (0.694-1.450) | 0.986 | 0.997 (0.689-1.442) | 0.987 | 0.987 |  | 1.269 (0.767-2.100) | 0.353 | 1.267 (0.765-2.099) | 0.357 | 0.714 |
| Low complement | 0.822 (0.568-1.190) | 0.300 | 0.809 (0.558-1.174) | 0.265 | 0.971 |  | 0.629 (0.380-1.041) | 0.0712 | 0.611 (0.368-1.016) | 0.058 | 0.580 |
| rs12161908 |  |  |  |  |  |  |  |  |  |  |  |
| Lupus nephritis | 1.048 (0.563-1.954) | 0.882 | 1.053 (0.565-1.964) | 0.870 | 0.959 |  | − | − | − | − | − |
| [Photosensitization](D:/%E6%96%87%E4%BB%B6/Dict/8.5.0.0/resultui/html/index.html#/javascript:;) | 1.208 (0.675-2.162) | 0.525 | 1.224 (0.683-2.195) | 0.497 | 0.959 |  | 0.806 (0.073-8.952) | 0.861 | 0.774 (0.069-8.653) | 0.835 | 0.835 |
| [Arthritis](D:/%E6%96%87%E4%BB%B6/Dict/8.5.0.0/resultui/html/index.html#/javascript:;) | 1.107 (0.623-1.967) | 0.729 | 1.098 (0.618-1.953) | 0.749 | 0.959 |  | 2.377 (0.214-26.383) | 0.481 | 2.251 (0.202-25.141) | 0.510 | 0.831 |
| [Erythema](D:/%E6%96%87%E4%BB%B6/Dict/8.5.0.0/resultui/html/index.html#/javascript:;) | 1.316 (1.316-2.351) | 0.354 | 1.314 (0.734-2.352) | 0.358 | 0.959 |  | 1.883 (0.170-20.899) | 0.606 | 1.614 (0.145-18.032) | 0.697 | 0.831 |
| [Alopecia](D:/%E6%96%87%E4%BB%B6/Dict/8.5.0.0/resultui/html/index.html#/javascript:;) | 0.694 (0.359-1.340) | 0.277 | 0.693 (0.357-1.342) | 0.277 | 0.959 |  | 4.357 (0.392-48.404) | 0.231 | 3.404 (0.304-38.081) | 0.320 | 0.812 |
| [Canker](D:/%E6%96%87%E4%BB%B6/Dict/8.5.0.0/resultui/html/index.html#/javascript:;) [sore](D:/%E6%96%87%E4%BB%B6/Dict/8.5.0.0/resultui/html/index.html#/javascript:;) | 0.912 (0.428-1.943) | 0.811 | 0.897 (0.420-1.915) | 0.779 | 0.959 |  | − | − | − | − | − |
| P[leurisy](D:/%E6%96%87%E4%BB%B6/Dict/8.5.0.0/resultui/html/index.html#/javascript:;) | 1.188 (0.445-3.168) | 0.731 | 1.193 (0.447-3.185) | 0.725 | 0.959 |  | − | − | − | − | − |
| Hematologic disorder | 0.997 (0.561-1.771) | 0.992 | 0.985 (0.554-1.752) | 0.959 | 0.959 |  | 0.460 (0.041-5.102) | 0.527 | 0.471 (0.042-5.263) | 0.211 | 0.812 |
| Anti-dsDNA | 0.817 (0.454-1.473) | 0.502 | 0.811 (0.450-1.463) | 0.486 | 0.959 |  | 0.666 (0.060-7.392) | 0.741 | 0.634 (0.057-7.086) | 0.712 | 0.831 |
| Low complement | 0.973 (0.546-1.734) | 0.926 | 0.969 (0.542-1.732) | 0.916 | 0.959 |  | 0.385 (0.035-4.271) | 0.437 | 0.315 (0.028-3.519) | 0.348 | 0.812 |
| rs2273440 |  |  |  |  |  |  |  |  |  |  |  |
| Lupus nephritis | 0.840 (0.571-1.235) | 0.375 | 0.835 (0.567-1.229) | 0.360 | 0.599 |  | 0.841 (0.422-1.676) | 0.622 | 0.845 (0.423-1.687) | 0.633 | 0.713 |
| [Photosensitization](D:/%E6%96%87%E4%BB%B6/Dict/8.5.0.0/resultui/html/index.html#/javascript:;) | 0.587 (0.406-0.848) | **0.005** | 0.576 (0.398-0.834) | **0.004** | **0.040** |  | 0.560 (0.282-1.113) | 0.098 | 0.571 (0.287-1.136) | 0.110 | 0.713 |
| [Arthritis](D:/%E6%96%87%E4%BB%B6/Dict/8.5.0.0/resultui/html/index.html#/javascript:;) | 1.048 (0.737-1.492) | 0.793 | 1.058 (0.743-1.507) | 0.756 | 0.841 |  | 0.696 (0.370-1.306) | 0.259 | 0.688 (0.366-1.293) | 0.246 | 0.713 |
| [Erythema](D:/%E6%96%87%E4%BB%B6/Dict/8.5.0.0/resultui/html/index.html#/javascript:;) | 0.783 (0.550-1.113) | 0.173 | 0.784 (0.550-1.117) | 0.177 | 0.590 |  | 0.888 (0.481-1.638) | 0.704 | 0.891 (0.481-1.648) | 0.713 | 0.713 |
| [Alopecia](D:/%E6%96%87%E4%BB%B6/Dict/8.5.0.0/resultui/html/index.html#/javascript:;) | 0.979 (0.670-1.428) | 0.911 | 0.977 (0.667-1.432) | 0.907 | 0.907 |  | 0.865 (0.441-1.698) | 0.674 | 0.874 (0.443-1.725) | 0.698 | 0.713 |
| [Canker](D:/%E6%96%87%E4%BB%B6/Dict/8.5.0.0/resultui/html/index.html#/javascript:;) [sore](D:/%E6%96%87%E4%BB%B6/Dict/8.5.0.0/resultui/html/index.html#/javascript:;) | 1.245 (0.793-1.955) | 0.341 | 1.267 (0.806-1.992) | 0.305 | 0.599 |  | 0.654 (0.269-1.594) | 0.351 | 0.632 (0.258-1.544) | 0.314 | 0.713 |
| P[leurisy](D:/%E6%96%87%E4%BB%B6/Dict/8.5.0.0/resultui/html/index.html#/javascript:;) | 0.585 (0.585-1.140) | 0.115 | 0.581 (0.298-1.134) | 0.112 | 0.560 |  | 0.231 (0.031-1.717) | 0.152 | 0.232 (0.031-1.732) | 0.155 | 0.713 |
| Hematologic disorder | 1.156 (0.813-1.644) | 0.420 | 1.173 (0.823-1.670) | 0.377 | 0.599 |  | 1.171 (0.632-2.167) | 0.616 | 1.147 (0.618-2.127) | 0.664 | 0.713 |
| Anti-dsDNA | 1.148 (0.805-1.638) | 0.446 | 1.158 (0.811-1.654) | 0.419 | 0.599 |  | 1.186 (0.642-2.193) | 0.586 | 1.176 (0.636-2.177) | 0.605 | 0.713 |
| Low complement | 1.054 (0.739-1.502) | 0.773 | 1.058 (0.740-1.512) | 0.757 | 0.841 |  | 0.792 0.429-1.462) | 0.456 | 0.796 (0.429-1.475) | 0.468 | 0.713 |
| rs3818188 |  |  |  |  |  |  |  |  |  |  |  |
| Lupus nephritis | 1.212 (0.782-1.877) | 0.389 | 1.221 (0.788-1.894) | 0.371 | 0.669 |  | 1.320 (0.845-2.062) | 0.222 | 1.349 (0.860-2.116) | 0.192 | 0.628 |
| [Photosensitization](D:/%E6%96%87%E4%BB%B6/Dict/8.5.0.0/resultui/html/index.html#/javascript:;) | 1.208 (0.803-1.819) | 0.365 | 1.227 (0.814-1.850) | 0.329 | 0.669 |  | 1.447 (0.947-2.211) | 0.088 | 1.513 (0.985-2.324) | 0.058 | 0.580 |
| [Arthritis](D:/%E6%96%87%E4%BB%B6/Dict/8.5.0.0/resultui/html/index.html#/javascript:;) | 1.074 (0.724-1.594) | 0.723 | 1.062 (0.715-1.578) | 0.764 | 0.770 |  | 1.160 (0.762-1.764) | 0.489 | 1.131 (0.741-1.726) | 0.568 | 0.987 |
| [Erythema](D:/%E6%96%87%E4%BB%B6/Dict/8.5.0.0/resultui/html/index.html#/javascript:;) | 1.070 (0.722-1.585) | 0.736 | 1.061 (0.715-1.574) | 0.770 | 0.770 |  | 1.299 (0.852-1.981) | 0.224 | 1.283 (0.838-1.965) | 0.251 | 0.628 |
| [Alopecia](D:/%E6%96%87%E4%BB%B6/Dict/8.5.0.0/resultui/html/index.html#/javascript:;) | 1.426 (0.920-2.210) | 0.112 | 1.420 (0.913-2.209) | 0.119 | 0.669 |  | 1.375 (0.887-2.133) | 0.155 | 1.365 (0.874-2.131) | 0.171 | 0.628 |
| [Canker](D:/%E6%96%87%E4%BB%B6/Dict/8.5.0.0/resultui/html/index.html#/javascript:;) [sore](D:/%E6%96%87%E4%BB%B6/Dict/8.5.0.0/resultui/html/index.html#/javascript:;) | 1.357 (0.798-2.309) | 0.260 | 1.337 (0.785-2.278) | 0.286 | 0.669 |  | 1.005 (0.586-1.722) | 0.986 | 0.959 (0.557-1.649) | 0.988 | 0.988 |
| P[leurisy](D:/%E6%96%87%E4%BB%B6/Dict/8.5.0.0/resultui/html/index.html#/javascript:;) | 1.413 (0.658-3.036) | 0.375 | 1.415 (0.658-3.045) | 0.374 | 0.669 |  | 0.934 (0.433-2.014) | 0.861 | 0.936 (0.432-2.030) | 0.867 | 0.988 |
| Hematologic disorder | 0.859 (0.579-1.274) | 0.450 | 0.847 (0.570-1.258) | 0.410 | 0.669 |  | 1.161 (0.762-1.767) | 0.487 | 1.123 (0.735-1.716) | 0.592 | 0.987 |
| Anti-dsDNA | 0.873 (0.588-1.297) | 0.501 | 0.864 (0.581-1.284) | 0.468 | 0.669 |  | 1.029 (0.674-1.570) | 0.896 | 1.004 (0.656-1.537) | 0.986 | 0.988 |
| Low complement | 0.930 (0.626-1.384) | 0.722 | 0.920 (0.617-1.372) | 0.682 | 0.770 |  | 1.014 0.664-1.547) | 0.949 | 0.996 (0.649-1.527) | 0.984 | 0.988 |
| *OR*, odds ratio; *CI*, confidence interval; *BH*, Benjamini-Hochberg method based on the false discovery rate;  **^*^**Adjusted for sex, age | | | | | | | | | | | |

| **Table S12** Association between DYNC1H1 polymorphisms and the clinical manifestations of SLE patients (male) | | | | | | | | | | | |  |  |
| --- | --- | --- | --- | --- | --- | --- | --- | --- | --- | --- | --- | --- | --- |
| **Polymorphisms** | **Dominant model** | | | | |  | **Recessive model** | | | | | |  |
|  | **Crude**  ***OR* (*95% CI*)** | **Crude**  ***P* value** | **Adjusted^*^**  ***OR* (*95% CI*)** | **Adjusted *P* value** | ***P_BH_*** |  | **Crude**  ***OR* (*95% CI*)** | **Crude**  ***P* value** | **Adjusted^*^**  ***OR* (*95% CI*)** | **Adjusted *P* value** | ***P_BH_*** | | |
|  |  |  |  |  |  |  |  |  |  |  |  |  |  |
| rs1004903 |  |  |  |  |  |  |  |  |  |  |  | | |
| Lupus nephritis | 0.312(0.092-1.058) | 0.062 | 0.318(0.093-1.085) | 0.067 | 0.350 |  | 0.969(0.082-11.513) | 0.980 | 1.118(0.090-13.837) | 0.931 | 0.931 | | |
| Photosensitization | 0.630(0.205-1.935) | 0.420 | 0.639(0.207-1.972) | 0.436 | 0.540 |  | 0.568(0.048-6.703) | 0.654 | 0.602(0.050-7.311) | 0.690 | 0.931 | | |
| [Arthritis](file:///D:\%25E6%2596%2587%25E4%25BB%25B6\Dict\8.5.0.0\resultui\html\index.html#/javascript:;) | 2.631(0.794-8.721) | 0.114 | 2.550(0.764-8.518) | 0.128 | 0.427 |  | 0.806(0.068-9.537) | 0.864 | 0.678(0.055-8.330) | 0.762 | 0.931 | | |
| [Erythema](file:///D:\%25E6%2596%2587%25E4%25BB%25B6\Dict\8.5.0.0\resultui\html\index.html#/javascript:;) | 0.750(0.244-2.303) | 0.615 | 0.699(0.222-2.198) | 0.540 | 0.540 |  | 2.700(0.229-31.891) | 0.431 | 2.266(0.186-27.670) | 0.522 | 0.931 | | |
| [Alopecia](file:///D:\%25E6%2596%2587%25E4%25BB%25B6\Dict\8.5.0.0\resultui\html\index.html#/javascript:;) | 2.000(0.514-7.779) | 0.317 | 2.004(0.513-7.831) | 0.318 | 0.454 |  | − | − | − | − | − | | |
| [Canker](file:///D:\%25E6%2596%2587%25E4%25BB%25B6\Dict\8.5.0.0\resultui\html\index.html#/javascript:;) [sore](file:///D:\%25E6%2596%2587%25E4%25BB%25B6\Dict\8.5.0.0\resultui\html\index.html#/javascript:;) | 0.138(0.015-1.287) | 0.082 | 0.100(0.008-1.206) | 0.070 | 0.350 |  | − | − | − | − | − | | |
| P[leurisy](file:///D:\%25E6%2596%2587%25E4%25BB%25B6\Dict\8.5.0.0\resultui\html\index.html#/javascript:;) | 0.256(0.025-2.654) | 0.254 | 0.239(0.022-2.558) | 0.237 | 0.454 |  | − | − | − | − | − | | |
| Hematologic disorder | 0.539(0.174-1.671) | 0.285 | 0.555(0.178-1.731) | 0.310 | 0.454 |  | 0.619(0.052-7.307) | 0.703 | 0.708(0.058-8.649) | 0.787 | 0.931 | | |
| Anti-dsDNA | 1.571(0.486-5.083) | 0.451 | 1.505(0.460-4.928) | 0.499 | 0.540 |  | 3.872(0.326-46.003) | 0.284 | 3.369(0.275-41.220) | 0.342 | 0.931 | | |
| Low complement | 1.944(0.628-6.021) | 0.249 | 1.982(0.635-6.181) | 0.239 | 0.454 |  | − | − | − | − | − | | |
| rs11160668 |  |  |  |  |  |  |  |  |  |  |  | | |
| Lupus nephritis | 1.195(0.370-3.858) | 0.765 | 1.172(0.361-3.807) | 0.792 | 0.990 |  | 2.067(0.265-16.128) | 0.489 | 2.015(0.258-15.768) | 0.504 | 0.769 | | |
| [Photosensitization](file:///D:\%25E6%2596%2587%25E4%25BB%25B6\Dict\8.5.0.0\resultui\html\index.html#/javascript:;) | 0.615(0.201-1.887) | 0.396 | 0.606(0.197-1.867) | 0.383 | 0.818 |  | 1.190(0.154-9.192) | 0.867 | 1.171(0.151-9.064) | 0.880 | 0.880 | | |
| [Arthritis](file:///D:\%25E6%2596%2587%25E4%25BB%25B6\Dict\8.5.0.0\resultui\html\index.html#/javascript:;) | 0.599(0.189-1.898) | 0.384 | 0.613(0.192-1.958) | 0.409 | 0.818 |  | 1.706(0.220-13.243) | 0.610 | 1.797(0.230-14.061) | 0.577 | 0.769 | | |
| [Erythema](file:///D:\%25E6%2596%2587%25E4%25BB%25B6\Dict\8.5.0.0\resultui\html\index.html#/javascript:;) | 1.385(0.451-4.246) | 0.569 | 1.453(0.465-4.540) | 0.521 | 0.868 |  | 0.397(0.038-4.105) | 0.438 | 0.417(0.040-4.342) | 0.465 | 0.769 | | |
| [Alopecia](file:///D:\%25E6%2596%2587%25E4%25BB%25B6\Dict\8.5.0.0\resultui\html\index.html#/javascript:;) | 0.242(0.056-1.040) | 0.057 | 0.242(0.056-1.040) | 0.057 | 0.285 |  | − | − | − | − | − | | |
| [Canker](file:///D:\%25E6%2596%2587%25E4%25BB%25B6\Dict\8.5.0.0\resultui\html\index.html#/javascript:;) [sore](file:///D:\%25E6%2596%2587%25E4%25BB%25B6\Dict\8.5.0.0\resultui\html\index.html#/javascript:;) | 1.000(0.182-5.507) | 1.000 | 0.989(0.167-5.863) | 0.990 | 0.990 |  | − | − | − | − | − | | |
| P[leurisy](file:///D:\%25E6%2596%2587%25E4%25BB%25B6\Dict\8.5.0.0\resultui\html\index.html#/javascript:;) | 3.272(0.317-33.830) | 0.320 | 3.397(0.324-35.621) | 0.308 | 0.818 |  | 21.999(1.959-247.049) | **0.012** | 26.025(2.001-338.571) | **0.013** | 0.104 | | |
| Hematologic disorder | 1.000(0.327-3.055) | 1.000 | 0.976(0.317-3.006) | 0.966 | 0.990 |  | 1.300(0.168-10.046) | 0.801 | 1.259(0.162-9.767) | 0.825 | 0.880 | | |
| Anti-dsDNA | 1.000(0.315-3.174) | 1.000 | 1.036(0.322-3.329) | 0.953 | 0.990 |  | 1.875(0.241-14.590) | 0.548 | 1.991(0.253-15.637) | 0.513 | 0.769 | | |
| Low complement | 0.265(0.082-0.855) | 0.026 | 0.260(0.080-0.845) | **0.025** | 0.250 |  | 0.333(0.032-3.446) | 0.357 | 0.329(0.032-3.408) | 0.351 | 0.769 | | |
| rs1190605 |  |  |  |  |  |  |  |  |  |  |  | | |
| Lupus nephritis | 0.263(0.077-0.901) | **0.034** | 0.272(0.078-0.948) | **0.041** | 0.410 |  | 0.969(0.082-11.513) | 0.980 | 1.118(0.090-13.837) | 0.931 | 0.931 | | |
| [Photosensitization](file:///D:\%25E6%2596%2587%25E4%25BB%25B6\Dict\8.5.0.0\resultui\html\index.html#/javascript:;) | 1.289(0.408-4.077) | 0.666 | 1.355(0.419-4.382) | 0.612 | 0.680 |  | 0.568(0.048-6.703) | 0.654 | 0.602(0.050-7.311) | 0.690 | 0.931 | | |
| [Arthritis](file:///D:\%25E6%2596%2587%25E4%25BB%25B6\Dict\8.5.0.0\resultui\html\index.html#/javascript:;) | 2.306(0.666-7.986) | 0.187 | 2.160(0.611-7.630) | 0.231 | 0.578 |  | 0.806(0.068-9.537) | 0.864 | 0.678(0.055-8.330) | 0.762 | 0.931 | | |
| [Erythema](file:///D:\%25E6%2596%2587%25E4%25BB%25B6\Dict\8.5.0.0\resultui\html\index.html#/javascript:;) | 1.607(0.500-5.170) | 0.426 | 1.456(0.441-4.810) | 0.537 | 0.671 |  | 2.700(0.229-31.891) | 0.431 | 2.266(0.186-27.670) | 0.522 | 0.931 | | |
| [Alopecia](file:///D:\%25E6%2596%2587%25E4%25BB%25B6\Dict\8.5.0.0\resultui\html\index.html#/javascript:;) | 0.817(0.217-3.068) | 0.764 | 0.805(0.209-3.100) | 0.753 | 0.753 |  | − | − | − | − | − | | |
| [Canker](file:///D:\%25E6%2596%2587%25E4%25BB%25B6\Dict\8.5.0.0\resultui\html\index.html#/javascript:;) [sore](file:///D:\%25E6%2596%2587%25E4%25BB%25B6\Dict\8.5.0.0\resultui\html\index.html#/javascript:;) | 0.259(0.042-1.577) | 0.143 | 0.268(0.040-1.803) | 0.176 | 0.578 |  | − | − | − | − | − | | |
| P[leurisy](file:///D:\%25E6%2596%2587%25E4%25BB%25B6\Dict\8.5.0.0\resultui\html\index.html#/javascript:;) | 0.178(0.017-1.851) | 0.149 | 0.143(0.012-1.682) | 0.122 | 0.578 |  | − | − | − | − | − | | |
| Hematologic disorder | 1.607(0.500-5.170) | 0.426 | 1.786(0.537-5.941) | 0.344 | 0.613 |  | 0.619(0.052-7.307) | 0.703 | 0.708(0.058-8.649) | 0.787 | 0.931 | | |
| Anti-dsDNA | 0.655(0.201-2.135) | 0.482 | 0.567(0.165-1.946) | 0.368 | 0.613 |  | 3.872(0.326-46.003) | 0.284 | 3.369(0.275-41.220) | 0.342 | 0.931 | | |
| Low complement | 1.467(0.464-4.638) | 0.514 | 1.526(0.472-4.934) | 0.480 | 0.671 |  | − | − | − | − | − | | |
| rs1190606 |  |  |  |  |  |  |  |  |  |  |  | | |
| Lupus nephritis | 0.304(0.090-1.029) | 0.056 | 0.315(0.092-1.074) | 0.065 | 0.325 |  | 0.747(0.129-4.321) | 0.744 | 0.779(0.133-4.563) | 0.782 | 0.877 | | |
| [Photosensitization](file:///D:\%25E6%2596%2587%25E4%25BB%25B6\Dict\8.5.0.0\resultui\html\index.html#/javascript:;) | 0.765(0.246-2.381) | 0.643 | 0.784(0.249-2.468) | 0.677 | 0.752 |  | 0.419(0.073-2.400) | 0.329 | 0.427(0.074-2.456) | 0.340 | 0.680 | | |
| [Arthritis](file:///D:\%25E6%2596%2587%25E4%25BB%25B6\Dict\8.5.0.0\resultui\html\index.html#/javascript:;) | 4.000(1.081-14.805) | **0.038** | 3.833(1.026-14.323) | **0.046** | 0.325 |  | 0.612(0.106-3.521) | 0.582 | 0.570(0.097-3.352) | 0.534 | 0.712 | | |
| [Erythema](file:///D:\%25E6%2596%2587%25E4%25BB%25B6\Dict\8.5.0.0\resultui\html\index.html#/javascript:;) | 0.935(0.299-2.920) | 0.907 | 0.848(0.264-2.725) | 0.782 | 0.782 |  | 0.947(0.189-4.756) | 0.948 | 0.878(0.170-4.529) | 0.877 | 0.877 | | |
| [Alopecia](file:///D:\%25E6%2596%2587%25E4%25BB%25B6\Dict\8.5.0.0\resultui\html\index.html#/javascript:;) | 1.454(0.373-5.679) | 0.590 | 1.457(0.369-5.761) | 0.591 | 0.739 |  | 0.485(0.052-4.487) | 0.524 | 0.481(0.052-4.475) | 0.520 | 0.712 | | |
| [Canker](file:///D:\%25E6%2596%2587%25E4%25BB%25B6\Dict\8.5.0.0\resultui\html\index.html#/javascript:;) [sore](file:///D:\%25E6%2596%2587%25E4%25BB%25B6\Dict\8.5.0.0\resultui\html\index.html#/javascript:;) | 0.286(0.047-1.737) | 0.174 | 0.280(0.041-1.890) | 0.191 | 0.472 |  | − | − | − | − | − | | |
| P[leurisy](file:///D:\%25E6%2596%2587%25E4%25BB%25B6\Dict\8.5.0.0\resultui\html\index.html#/javascript:;) | 0.195(0.019-2.031) | 0.172 | 0.170(0.015-1.900) | 0.150 | 0.472 |  | − | − | − | − | − | | |
| Hematologic disorder | 0.474(0.150-1.499) | 0.204 | 0.495(0.155-1.583) | 0.236 | 0.472 |  | 3.822(0.664-21.993) | 0.133 | 4.172(0.703-24.746) | 0.116 | 0.464 | | |
| Anti-dsDNA | 1.556(0.467-5.182) | 0.472 | 1.452(0.428-4.928) | 0.550 | 0.739 |  | 5.769(0.988-33.676) | 0.052 | 5.592(0.947-33.038) | 0.058 | 0.464 | | |
| Low complement | 1.714(0.545-5.396) | 0.357 | 1.770(0.554-5.653) | 0.335 | 0.558 |  | 3.158(0.551-18.114) | 0.197 | 3.243(0.560-18.789) | 0.189 | 0.504 | | |
| rs12161908 |  |  |  |  |  |  |  |  |  |  |  | | |
| Lupus nephritis | 0.625(0.060-6.509) | 0.694 | 0.648(0.061-6.842) | 0.718 | 0.862 |  | − | − | − | − | − | | |
| [Photosensitization](file:///D:\%25E6%2596%2587%25E4%25BB%25B6\Dict\8.5.0.0\resultui\html\index.html#/javascript:;) | 3.900(0.377-40.367) | 0.254 | 4.044(0.386-42.403) | 0.244 | 0.732 |  | − | − | − | − | − | | |
| [Arthritis](file:///D:\%25E6%2596%2587%25E4%25BB%25B6\Dict\8.5.0.0\resultui\html\index.html#/javascript:;) | 0.519(0.050-5.379) | 0.582 | 0.480(0.045-5.135) | 0.544 | 0.816 |  | − | − | − | − | − | | |
| [Erythema](file:///D:\%25E6%2596%2587%25E4%25BB%25B6\Dict\8.5.0.0\resultui\html\index.html#/javascript:;) | 4.263(0.411-44.170) | 0.224 | 4.152(0.389-44.290) | 0.239 | 0.732 |  | − | − | − | − | − | | |
| [Alopecia](file:///D:\%25E6%2596%2587%25E4%25BB%25B6\Dict\8.5.0.0\resultui\html\index.html#/javascript:;) | − | − | − | − | − |  | − | − | − | − | − | | |
| [Canker](file:///D:\%25E6%2596%2587%25E4%25BB%25B6\Dict\8.5.0.0\resultui\html\index.html#/javascript:;) [sore](file:///D:\%25E6%2596%2587%25E4%25BB%25B6\Dict\8.5.0.0\resultui\html\index.html#/javascript:;) | 2.734(0.237-31.557) | 0.420 | 3.368(0.227-50.038) | 0.378 | 0.756 |  | − | − | − | − | − | | |
| P[leurisy](file:///D:\%25E6%2596%2587%25E4%25BB%25B6\Dict\8.5.0.0\resultui\html\index.html#/javascript:;) | − | − | − | − | − |  | − | − | − | − | − | | |
| Hematologic disorder | − | − | − | − | − |  | − | − | − | − | − | | |
| Anti-dsDNA | − | − | − | − | − |  | − | − | − | − | − | | |
| Low complement | 1.091(0.141-8.420) | 0.933 | 1.108(0.143-8.596) | 0.922 | 0.922 |  | − | − | − | − | − | | |
| rs2273440 |  |  |  |  |  |  |  |  |  |  |  | | |
| Lupus nephritis | 0.355(0.105-1.195) | 0.095 | 0.366(0.108-1.245) | 0.108 | 0.473 |  | 0.969(0.082-11.513) | 0.980 | 1.118(0.090-13.837) | 0.931 | 0.931 | | |
| [Photosensitization](file:///D:\%25E6%2596%2587%25E4%25BB%25B6\Dict\8.5.0.0\resultui\html\index.html#/javascript:;) | 0.529(0.171-1.631) | 0.268 | 0.538(0.173-1.673) | 0.284 | 0.473 |  | 0.568(0.048-6.703) | 0.654 | 0.602(0.050-7.311) | 0.690 | 0.931 | | |
| [Arthritis](file:///D:\%25E6%2596%2587%25E4%25BB%25B6\Dict\8.5.0.0\resultui\html\index.html#/javascript:;) | 2.081(0.646-6.707) | 0.220 | 1.981(0.607-6.465) | 0.257 | 0.473 |  | 0.806(0.068-9.537) | 0.864 | 0.678(0.055-8.330) | 0.762 | 0.931 | | |
| [Erythema](file:///D:\%25E6%2596%2587%25E4%25BB%25B6\Dict\8.5.0.0\resultui\html\index.html#/javascript:;) | 0.867(0.283-2.651) | 0.802 | 0.789(0.250-2.485) | 0.686 | 0.686 |  | 2.700(0.229-31.891) | 0.431 | 2.266(0.186-27.670) | 0.522 | 0.931 | | |
| [Alopecia](file:///D:\%25E6%2596%2587%25E4%25BB%25B6\Dict\8.5.0.0\resultui\html\index.html#/javascript:;) | 2.222(0.571-8.647) | 0.249 | 2.241(0.570-8.811) | 0.248 | 0.473 |  | − | − | − | − | − | | |
| [Canker](file:///D:\%25E6%2596%2587%25E4%25BB%25B6\Dict\8.5.0.0\resultui\html\index.html#/javascript:;) [sore](file:///D:\%25E6%2596%2587%25E4%25BB%25B6\Dict\8.5.0.0\resultui\html\index.html#/javascript:;) | 0.152(0.016-1.411) | 0.098 | 0.127(0.011-1.432) | 0.095 | 0.473 |  | − | − | − | − | − | | |
| P[leurisy](file:///D:\%25E6%2596%2587%25E4%25BB%25B6\Dict\8.5.0.0\resultui\html\index.html#/javascript:;) | 0.280(0.027-2.896) | 0.286 | 0.253(0.023-2.751) | 0.259 | 0.473 |  | − | − | − | − | − | | |
| Hematologic disorder | 0.625(0.203-1.924) | 0.413 | 0.654(0.210-2.036) | 0.463 | 0.514 |  | 0.619(0.052-7.307) | 0.703 | 0.708(0.058-8.649) | 0.787 | 0.931 | | |
| Anti-dsDNA | 1.781(0.550-5.765) | 0.336 | 1.679(0.511-5.522) | 0.393 | 0.491 |  | 3.872(0.326-46.003) | 0.284 | 3.369(0.275-41.220) | 0.342 | 0.931 | | |
| Low complement | 1.633(0.533-5.003) | 0.390 | 1.679(0.541-5.209) | 0.370 | 0.491 |  | − | − | − | − | − | | |
| rs3818188 |  |  |  |  |  |  |  |  |  |  |  | | |
| Lupus nephritis | 2.112(0.564-7.911) | 0.267 | 2.068(0.549-7.789) | 0.283 | 0.648 |  | 2.067(0.265-16.128) | 0.489 | 1.949(0.247-15.400) | 0.527 | 0.947 | | |
| [Photosensitization](file:///D:\%25E6%2596%2587%25E4%25BB%25B6\Dict\8.5.0.0\resultui\html\index.html#/javascript:;) | 1.948(0.583-6.509) | 0.279 | 1.927(0.575-6.456) | 0.288 | 0.648 |  | 1.190(0.154-9.192) | 0.867 | 1.150(0.147-8.971) | 0.894 | 0.957 | | |
| [Arthritis](file:///D:\%25E6%2596%2587%25E4%25BB%25B6\Dict\8.5.0.0\resultui\html\index.html#/javascript:;) | 0.816(0.246-2.706) | 0.740 | 0.842(0.252-2.819) | 0.781 | 0.810 |  | 0.519(0.050-5.379) | 0.582 | 0.561(0.053-5.915) | 0.631 | 0.947 | | |
| [Erythema](file:///D:\%25E6%2596%2587%25E4%25BB%25B6\Dict\8.5.0.0\resultui\html\index.html#/javascript:;) | 1.190(0.364-3.891) | 0.773 | 1.250(0.376-4.158) | 0.716 | 0.810 |  | − | − | − | − | − | | |
| [Alopecia](file:///D:\%25E6%2596%2587%25E4%25BB%25B6\Dict\8.5.0.0\resultui\html\index.html#/javascript:;) | − | − | − | − | − |  | 1.061(0.100-11.260) | 0.961 | 1.068(0.099-11.461) | 0.957 | 0.957 | | |
| [Canker](file:///D:\%25E6%2596%2587%25E4%25BB%25B6\Dict\8.5.0.0\resultui\html\index.html#/javascript:;) [sore](file:///D:\%25E6%2596%2587%25E4%25BB%25B6\Dict\8.5.0.0\resultui\html\index.html#/javascript:;) | 2.857(0.306-26.650) | 0.357 | 2.753(0.273-27.798) | 0.391 | 0.704 |  | 10.499(1.149-95.905) | 0.037 | 10.706(1.003-114.236) | 0.050 | 0.450 | | |
| P[leurisy](file:///D:\%25E6%2596%2587%25E4%25BB%25B6\Dict\8.5.0.0\resultui\html\index.html#/javascript:;) | 0.146(0.014-1.527) | 0.108 | 0.148(0.014-1.555) | 0.111 | 0.648 |  | 4.778(0.374-61.061) | 0.229 | 5.344(0.391-73.045) | 0.209 | 0.941 | | |
| Hematologic disorder | 1.190(0.364-3.891) | 0.773 | 1.157(0.351-3.815) | 0.810 | 0.810 |  | 0.397(0.038-4.105) | 0.438 | 0.363(0.034-3.825) | 0.399 | 0.947 | | |
| Anti-dsDNA | 0.489(0.146-1.636) | 0.246 | 0.501(0.149-1.693) | 0.266 | 0.648 |  | 1.875(0.241-14.590) | 0.548 | 2.105(0.262-16.902) | 0.484 | 0.947 | | |
| Low complement | 1.518(0.465-4.953) | 0.489 | 1.507(0.461-4.930) | 0.498 | 0.747 |  | 1.091(0.141-8.420) | 0.933 | 1.067(0.137-8.312) | 0.951 | 0.957 | | |
| *OR*, odds ratio; *CI*, confidence interval; *BH*, Benjamini-Hochberg method based on the false discovery rate;  **^*^**Adjusted for age | | | | | | | | | | | |  |  |

| **Table S13** Association between DYNC1H1 polymorphisms and the clinical manifestations of SLE patients (female) | | | | | | | | | | | |
| --- | --- | --- | --- | --- | --- | --- | --- | --- | --- | --- | --- |
| **Polymorphisms** | **Dominant model** | | | | |  | **Recessive model** | | | | |
|  | **Crude**  ***OR* (*95% CI*)** | **Crude**  ***P* value** | **Adjusted^*^**  ***OR* (*95% CI*)** | **Adjusted *P* value** | ***P_BH_*** |  | **Crude**  ***OR* (*95% CI*)** | **Crude**  ***P* value** | **Adjusted^*^**  ***OR* (*95% CI*)** | **Adjusted *P* value** | ***P_BH_*** |
| rs1004903 |  |  |  |  |  |  |  |  |  |  |  |
| Lupus nephritis | 0.918 (0.610-1.382) | 0.682 | 0.918 (0.610-1.382) | 0.682 | 0.844 |  | 0.845 (0.398-1.793) | 0.661 | 0.846 (0.398-1.798) | 0.664 | 0.738 |
| [Photosensitization](file:///D:\%25E6%2596%2587%25E4%25BB%25B6\Dict\8.5.0.0\resultui\html\index.html#/javascript:;) | 0.611 (0.413-0.903) | **0.013** | 0.608 (0.411-0.900) | **0.013** | 0.130 |  | 0.573 (0.271-1.211) | 0.145 | 0.585 (0.276-1.238) | 0.161 | 0.537 |
| [Arthritis](file:///D:\%25E6%2596%2587%25E4%25BB%25B6\Dict\8.5.0.0\resultui\html\index.html#/javascript:;) | 1.038 (0.715-1.507) | 0.844 | 1.038 (0.715-1.507) | 0.844 | 0.844 |  | 0.567 (0.282-1.139) | 0.111 | 0.566 (0.281-1.138) | 0.110 | 0.537 |
| [Erythema](file:///D:\%25E6%2596%2587%25E4%25BB%25B6\Dict\8.5.0.0\resultui\html\index.html#/javascript:;) | 0.797 (0.549-1.157) | 0.232 | 0.795 (0.547-1.155) | 0.228 | 0.570 |  | 0.801 (0.412-1.558) | 0.514 | 0.820 (0.420-1.599) | 0.560 | 0.705 |
| [Alopecia](file:///D:\%25E6%2596%2587%25E4%25BB%25B6\Dict\8.5.0.0\resultui\html\index.html#/javascript:;) | 0.846 (0.568-1.260) | 0.410 | 0.838 (0.561-1.253) | 0.389 | 0.648 |  | 0.722 (0.341-1.530) | 0.395 | 0.755 (0.354-1.611) | 0.468 | 0.705 |
| [Canker](file:///D:\%25E6%2596%2587%25E4%25BB%25B6\Dict\8.5.0.0\resultui\html\index.html#/javascript:;) [sore](file:///D:\%25E6%2596%2587%25E4%25BB%25B6\Dict\8.5.0.0\resultui\html\index.html#/javascript:;) | 1.501 (0.939-2.400) | 0.090 | 1.502 (0.939-2.401) | 0.089 | 0.403 |  | 0.771 (0.312-1.906) | 0.573 | 0.766 (0.309-1.896) | 0.564 | 0.705 |
| P[leurisy](file:///D:\%25E6%2596%2587%25E4%25BB%25B6\Dict\8.5.0.0\resultui\html\index.html#/javascript:;) | 0.571 (0.281-1.163) | 0.123 | 0.570 (0.280-1.160) | 0.121 | 0.403 |  | 0.275 (0.037-2.065) | 0.210 | 0.280 (0.037-2.105) | 0.216 | 0.540 |
| Hematologic disorder | 1.191 (0.820-1.730) | 0.359 | 1.192 (0.821-1.731) | 0.356 | 0.648 |  | 1.406 (0.713-2.770) | 0.325 | 1.392 (0.705-2.747) | 0.340 | 0.680 |
| Anti-dsDNA | 1.081 (0.743-1.573) | 0.682 | 1.082 (0.744-1.573) | 0.681 | 0.844 |  | 1.053 (0.539-2.053) | 0.881 | 1.049 (0.537-2.050) | 0.888 | 0.888 |
| Low complement | 1.042 (0.715-1.518) | 0.830 | 1.040 (0.712-1.517) | 0.840 | 0.844 |  | 0.575 (0.295-1.123) | 0.105 | 0.596 (0.304-1.170) | 0.133 | 0.537 |
| rs11160668 |  |  |  |  |  |  |  |  |  |  |  |
| Lupus nephritis | 0.887 (0.585-1.345) | 0.573 | 0.887 (0.585-1.344) | 0.571 | 0.933 |  | 0.676 (0.218-2.092) | 0.497 | 0.675 (0.218-2.090) | 0.496 | 0.827 |
| [Photosensitization](file:///D:\%25E6%2596%2587%25E4%25BB%25B6\Dict\8.5.0.0\resultui\html\index.html#/javascript:;) | 1.085 (0.736-1.601) | 0.680 | 1.079 (0.731-1.593) | 0.703 | 0.933 |  | 1.712 (0.666-4.403) | 0.264 | 1.702 (0.661-4.383) | 0.270 | 0.718 |
| [Arthritis](file:///D:\%25E6%2596%2587%25E4%25BB%25B6\Dict\8.5.0.0\resultui\html\index.html#/javascript:;) | 0.944 (0.647-1.377) | 0.764 | 0.944 (0.647-1.378) | 0.764 | 0.933 |  | 0.559 (0.206-1.515) | 0.253 | 0.559 (0.206-1.515) | 0.253 | 0.718 |
| [Erythema](file:///D:\%25E6%2596%2587%25E4%25BB%25B6\Dict\8.5.0.0\resultui\html\index.html#/javascript:;) | 0.969 (0.664-1.413) | 0.869 | 0.962 (0.659-1.404) | 0.842 | 0.933 |  | 0.903 (0.352-2.319) | 0.833 | 0.897 (0.349-2.305) | 0.821 | 0.912 |
| [Alopecia](file:///D:\%25E6%2596%2587%25E4%25BB%25B6\Dict\8.5.0.0\resultui\html\index.html#/javascript:;) | 0.950 (0.635-1.422) | 0.804 | 0.938 (0.625-1.408) | 0.757 | 0.933 |  | 1.698 (0.656-4.396) | 0.275 | 1.684 (0.645-4.392) | 0.287 | 0.718 |
| [Canker](file:///D:\%25E6%2596%2587%25E4%25BB%25B6\Dict\8.5.0.0\resultui\html\index.html#/javascript:;) [sore](file:///D:\%25E6%2596%2587%25E4%25BB%25B6\Dict\8.5.0.0\resultui\html\index.html#/javascript:;) | 0.717 (0.438-1.172) | 0.185 | 0.718 (0.439-1.174) | 0.186 | 0.933 |  | 1.208 (0.388-3.765) | 0.744 | 1.210 (0.388-3.773) | 0.742 | 0.912 |
| P[leurisy](file:///D:\%25E6%2596%2587%25E4%25BB%25B6\Dict\8.5.0.0\resultui\html\index.html#/javascript:;) | 1.588 (0.816-3.090) | 0.174 | 1.580 (0.812-3.077) | 0.178 | 0.933 |  | 1.383 (0.306-6.252) | 0.674 | 1.374 (0.304-6.217) | 0.680 | 0.912 |
| Hematologic disorder | 1.013 (0.694-1.478) | 0.946 | 1.016 (0.696-1.483) | 0.933 | 0.933 |  | 0.431 (0.159-1.170) | 0.099 | 0.433 (0.159-1.174) | 0.100 | 0.718 |
| Anti-dsDNA | 0.927 (0.634-1.356) | 0.696 | 0.928 (0.634-1.358) | 0.700 | 0.933 |  | 1.037 (0.402-2.679) | 0.940 | 1.038 (0.402-2.682) | 0.938 | 0.938 |
| Low complement | 1.183 (0.807-1.734) | 0.389 | 1.170 (0.797-1.719) | 0.422 | 0.933 |  | 1.514 (0.558-4.108) | 0.415 | 1.499 (0.550-4.083) | 0.429 | 0.827 |
| rs1190605 |  |  |  |  |  |  |  |  |  |  |  |
| Lupus nephritis | 0.844 (0.563-1.266) | 0.412 | 0.843 (0.562-1.264) | 0.409 | 0.682 |  | 1.020 (0.527-1.974) | 0.954 | 1.022 (0.527-1.980) | 0.949 | 0.949 |
| [Photosensitization](file:///D:\%25E6%2596%2587%25E4%25BB%25B6\Dict\8.5.0.0\resultui\html\index.html#/javascript:;) | 0.579 (0.394-0.851) | **0.005** | 0.570 (0.387-0.839) | **0.004** | **0.040** |  | 0.684 (0.355-1.319) | 0.257 | 0.697 (0.361-1.345) | 0.282 | 0.670 |
| [Arthritis](file:///D:\%25E6%2596%2587%25E4%25BB%25B6\Dict\8.5.0.0\resultui\html\index.html#/javascript:;) | 1.080 (0.746-1.563) | 0.683 | 1.081 (0.746-1.565) | 0.682 | 0.888 |  | 0.682 (0.367-1.267) | 0.226 | 0.681 (0.366-1.266) | 0.225 | 0.670 |
| [Erythema](file:///D:\%25E6%2596%2587%25E4%25BB%25B6\Dict\8.5.0.0\resultui\html\index.html#/javascript:;) | 0.864 (0.597-1.250) | 0.438 | 0.853 (0.589-1.236) | 0.401 | 0.682 |  | 0.941 (0.514-1.721) | 0.842 | 0.960 (0.523-1.760) | 0.895 | 0.949 |
| [Alopecia](file:///D:\%25E6%2596%2587%25E4%25BB%25B6\Dict\8.5.0.0\resultui\html\index.html#/javascript:;) | 0.847 (0.571-1.255) | 0.407 | 0.821 (0.552-1.222) | 0.331 | 0.682 |  | 0.686 (0.345-1.364) | 0.282 | 0.711 (0.355-1.423) | 0.335 | 0.670 |
| [Canker](file:///D:\%25E6%2596%2587%25E4%25BB%25B6\Dict\8.5.0.0\resultui\html\index.html#/javascript:;) [sore](file:///D:\%25E6%2596%2587%25E4%25BB%25B6\Dict\8.5.0.0\resultui\html\index.html#/javascript:;) | 1.374 (0.857-2.202) | 0.187 | 1.379 (0.859-2.212) | 0.183 | 0.682 |  | 0.711 (0.307-1.646) | 0.426 | 0.707 (0.305-1.639) | 0.419 | 0.698 |
| P[leurisy](file:///D:\%25E6%2596%2587%25E4%25BB%25B6\Dict\8.5.0.0\resultui\html\index.html#/javascript:;) | 0.673 (0.344-1.318) | 0.249 | 0.665 (0.339-1.304) | 0.235 | 0.682 |  | 0.456 (0.106-1.956) | 0.290 | 0.463 (0.108-1.990) | 0.301 | 0.670 |
| Hematologic disorder | 1.031 (0.712-1.492) | 0.872 | 1.036 (0.716-1.501) | 0.850 | 0.904 |  | 1.655 (0.885-3.095) | 0.115 | 1.642 (0.877-3.075) | 0.121 | 0.670 |
| Anti-dsDNA | 0.976 (0.673-1.415) | 0.897 | 0.977 (0.673-1.418) | 0.904 | 0.904 |  | 1.157 (0.631-2.120) | 0.638 | 1.154 (0.629-2.117) | 0.644 | 0.920 |
| Low complement | 0.952 (0.656-1.382) | 0.795 | 0.931 (0.640-1.356) | 0.710 | 0.888 |  | 0.914 (0.497-1.678) | 0.771 | 0.948 (0.514-1.750) | 0.865 | 0.949 |
| rs1190606 |  |  |  |  |  |  |  |  |  |  |  |
| Lupus nephritis | 0.958 (0.628-1.461) | 0.842 | 0.958 (0.628-1.461) | 0.842 | 0.960 |  | 1.203 (0.683-2.120) | 0.521 | 1.202 (0.682-2.119) | 0.524 | 0.873 |
| [Photosensitization](file:///D:\%25E6%2596%2587%25E4%25BB%25B6\Dict\8.5.0.0\resultui\html\index.html#/javascript:;) | 0.842 (0.567-1.252) | 0.396 | 0.841 (0.565-1.251) | 0.393 | 0.960 |  | 0.729 (0.414-1.285) | 0.275 | 0.718 (0.407-1.268) | 0.254 | 0.873 |
| [Arthritis](file:///D:\%25E6%2596%2587%25E4%25BB%25B6\Dict\8.5.0.0\resultui\html\index.html#/javascript:;) | 0.879 (0.598-1.294) | 0.514 | 0.879 (0.598-1.294) | 0.514 | 0.960 |  | 0.809 (0.474-1.381) | 0.437 | 0.809 (0.474-1.381) | 0.437 | 0.873 |
| [Erythema](file:///D:\%25E6%2596%2587%25E4%25BB%25B6\Dict\8.5.0.0\resultui\html\index.html#/javascript:;) | 0.853 (0.579-1.255) | 0.419 | 0.851 (0.578-1.254) | 0.416 | 0.960 |  | 0.960 (0.566-1.630) | 0.880 | 0.948 (0.558-1.612) | 0.844 | 0.920 |
| [Alopecia](file:///D:\%25E6%2596%2587%25E4%25BB%25B6\Dict\8.5.0.0\resultui\html\index.html#/javascript:;) | 1.094 (0.724-1.655) | 0.669 | 1.092 (0.720-1.656) | 0.680 | 0.960 |  | 0.786 (0.438-1.409) | 0.419 | 0.755 (0.418-1.362) | 0.350 | 0.873 |
| [Canker](file:///D:\%25E6%2596%2587%25E4%25BB%25B6\Dict\8.5.0.0\resultui\html\index.html#/javascript:;) [sore](file:///D:\%25E6%2596%2587%25E4%25BB%25B6\Dict\8.5.0.0\resultui\html\index.html#/javascript:;) | 0.987 (0.606-1.609) | 0.960 | 0.988 (0.606-1.610) | 0.960 | 0.960 |  | 0.963 (0.490-1.895) | 0.914 | 0.966 (0.491-1.900) | 0.920 | 0.920 |
| P[leurisy](file:///D:\%25E6%2596%2587%25E4%25BB%25B6\Dict\8.5.0.0\resultui\html\index.html#/javascript:;) | 1.380 (0.666-2.861) | 0.387 | 1.378 (0.665-2.859) | 0.389 | 0.960 |  | 0.912 (0.342-2.429) | 0.853 | 0.899 (0.337-2.400) | 0.832 | 0.920 |
| Hematologic disorder | 1.024 (0.696-1.506) | 0.906 | 1.024 (0.696-1.507) | 0.904 | 0.960 |  | 0.756 (0.445-1.284) | 0.300 | 0.760 (0.447-1.291) | 0.310 | 0.873 |
| Anti-dsDNA | 0.951 (0.645-1.403) | 0.802 | 0.952 (0.645-1.403) | 0.802 | 0.960 |  | 1.085 (0.638-1.847) | 0.763 | 1.087 (0.639-1.851) | 0.758 | 0.920 |
| Low complement | 0.747 (0.504-1.107) | 0.146 | 0.744 (0.501-1.105) | 0.142 | 0.960 |  | 0.529 (0.310-0.902) | **0.019** | 0.512 (0.299-0.877) | **0.015** | 0.150 |
| rs12161908 |  |  |  |  |  |  |  |  |  |  |  |
| Lupus nephritis | 1.102 (0.577-2.104) | 0.769 | 1.103 (0.577-2.106) | 0.768 | 0.861 |  | − | − | − | − | − |
| [Photosensitization](file:///D:\%25E6%2596%2587%25E4%25BB%25B6\Dict\8.5.0.0\resultui\html\index.html#/javascript:;) | 1.109 (0.601-2.047) | 0.740 | 1.118 (0.606-2.066) | 0.721 | 0.861 |  | 0.836 (0.075-9.293) | 0.884 | 0.753 (0.067-8.425) | 0.818 | 0.818 |
| [Arthritis](file:///D:\%25E6%2596%2587%25E4%25BB%25B6\Dict\8.5.0.0\resultui\html\index.html#/javascript:;) | 1.161 (0.638-2.112) | 0.625 | 1.161 (0.638-2.113) | 0.626 | 0.861 |  | 2.296 (0.207-25.507) | 0.499 | 2.312 (0.207-25.846) | 0.496 | 0.818 |
| [Erythema](file:///D:\%25E6%2596%2587%25E4%25BB%25B6\Dict\8.5.0.0\resultui\html\index.html#/javascript:;) | 1.188 (0.650-2.170) | 0.576 | 1.197 (0.655-2.189) | 0.559 | 0.861 |  | 1.821 (0.164-20.226) | 0.626 | 1.646 (0.147-18.398) | 0.686 | 0.818 |
| [Alopecia](file:///D:\%25E6%2596%2587%25E4%25BB%25B6\Dict\8.5.0.0\resultui\html\index.html#/javascript:;) | 0.748 (0.383-1.462) | 0.396 | 0.762 (0.389-1.494) | 0.429 | 0.861 |  | 4.193 (0.377-46.620) | 0.243 | 3.314 (0.296-37.104) | 0.331 | 0.818 |
| [Canker](file:///D:\%25E6%2596%2587%25E4%25BB%25B6\Dict\8.5.0.0\resultui\html\index.html#/javascript:;) [sore](file:///D:\%25E6%2596%2587%25E4%25BB%25B6\Dict\8.5.0.0\resultui\html\index.html#/javascript:;) | 0.823 (0.370-1.827) | 0.632 | 0.821 (0.370-1.825) | 0.629 | 0.861 |  | − | − | − | − | − |
| P[leurisy](file:///D:\%25E6%2596%2587%25E4%25BB%25B6\Dict\8.5.0.0\resultui\html\index.html#/javascript:;) | 1.308 (0.485-3.526) | 0.596 | 1.319 (0.489-3.560) | 0.585 | 0.861 |  | − | − | − | − | − |
| Hematologic disorder | 0.800 (0.440-1.457) | 0.466 | 0.798 (0.438-1.453) | 0.460 | 0.861 |  | 0.443 (0.040-4.924) | 0.508 | 0.463 (0.041-5.177) | 0.532 | 0.818 |
| Anti-dsDNA | 0.916 (0.500-1.681) | 0.778 | 0.915 (0.499-1.679) | 0.775 | 0.861 |  | 0.645 (0.058-7.170) | 0.722 | 0.653 (0.058-7.303) | 0.730 | 0.818 |
| Low complement | 0.953 (0.521-1.743) | 0.876 | 0.965 (0.527-1.769) | 0.909 | 0.909 |  | 0.370 (0.033-4.112) | 0.419 | 0.304 (0.027-3.397) | 0.333 | 0.818 |
| rs2273440 |  |  |  |  |  |  |  |  |  |  |  |
| Lupus nephritis | 0.923 (0.614-1.388) | 0.701 | 0.923 (0.614-1.388) | 0.700 | 0.875 |  | 0.838 (0.408-1.720) | 0.630 | 0.839 (0.408-1.725) | 0.633 | 0.791 |
| [Photosensitization](file:///D:\%25E6%2596%2587%25E4%25BB%25B6\Dict\8.5.0.0\resultui\html\index.html#/javascript:;) | 0.585 (0.396-0.865) | **0.007** | 0.583 (0.394-0.863) | **0.007** | 0.070 |  | 0.566 (0.277-1.158) | 0.119 | 0.576 (0.281-1.181) | 0.132 | 0.608 |
| [Arthritis](file:///D:\%25E6%2596%2587%25E4%25BB%25B6\Dict\8.5.0.0\resultui\html\index.html#/javascript:;) | 0.987 (0.680-1.432) | 0.945 | 0.987 (0.680-1.432) | 0.945 | 0.950 |  | 0.679 (0.353-1.303) | 0.244 | 0.677 (0.352-1.302) | 0.243 | 0.608 |
| [Erythema](file:///D:\%25E6%2596%2587%25E4%25BB%25B6\Dict\8.5.0.0\resultui\html\index.html#/javascript:;) | 0.781 (0.538-1.133) | 0.193 | 0.779 (0.537-1.131) | 0.190 | 0.470 |  | 0.808 (0.428-1.527) | 0.512 | 0.825 (0.436-1.562) | 0.555 | 0.791 |
| [Alopecia](file:///D:\%25E6%2596%2587%25E4%25BB%25B6\Dict\8.5.0.0\resultui\html\index.html#/javascript:;) | 0.920 (0.618-1.368) | 0.680 | 0.913 (0.612-1.362) | 0.655 | 0.875 |  | 0.923 0.465-1.833) | 0.820 | 0.964 (0.482-1.929) | 0.918 | 0.918 |
| [Canker](file:///D:\%25E6%2596%2587%25E4%25BB%25B6\Dict\8.5.0.0\resultui\html\index.html#/javascript:;) [sore](file:///D:\%25E6%2596%2587%25E4%25BB%25B6\Dict\8.5.0.0\resultui\html\index.html#/javascript:;) | 1.452 (0.908-2.320) | 0.119 | 1.452 (0.909-2.321) | 0.119 | 0.470 |  | 0.677 (0.276-1.662) | 0.395 | 0.673 (0.274-1.654) | 0.388 | 0.776 |
| P[leurisy](file:///D:\%25E6%2596%2587%25E4%25BB%25B6\Dict\8.5.0.0\resultui\html\index.html#/javascript:;) | 0.631 (0.314-1.268) | 0.196 | 0.630 (0.313-1.265) | 0.194 | 0.470 |  | 0.246 (0.033-1.839) | 0.172 | 0.250 (0.033-1.870) | 0.177 | 0.608 |
| Hematologic disorder | 1.253 (0.863-1.819) | 0.236 | 1.254 (0.864-1.820) | 0.235 | 0.470 |  | 1.209 (0.637-2.296) | 0.561 | 1.198 (0.630-2.278) | 0.581 | 0.791 |
| Anti-dsDNA | 1.109 (0.763-1.612) | 0.589 | 1.109 (0.763-1.612) | 0.588 | 0.875 |  | 1.077 (0.569-2.039) | 0.820 | 1.074 (0.567-2.035) | 0.827 | 0.918 |
| Low complement | 1.015 (0.697-1.477) | 0.939 | 1.012 (0.694-1.476) | 0.950 | 0.950 |  | 0.650 (0.344-1.229) | 0.185 | 0.672 (0.354-1.277) | 0.225 | 0.608 |
| rs3818188 |  |  |  |  |  |  |  |  |  |  |  |
| Lupus nephritis | 1.136 (0.714-1.808) | 0.589 | 1.136 (0.714-1.808) | 0.590 | 0.794 |  | 1.322 (0.834-2.097) | 0.235 | 1.323 (0.834-2.099) | 0.234 | 0.585 |
| [Photosensitization](file:///D:\%25E6%2596%2587%25E4%25BB%25B6\Dict\8.5.0.0\resultui\html\index.html#/javascript:;) | 1.151 (0.744-1.778) | 0.528 | 1.151 (0.744-1.780) | 0.528 | 0.794 |  | 1.518 (0.980-2.351) | 0.062 | 1.531 (0.987-2.375) | 0.057 | 0.563 |
| [Arthritis](file:///D:\%25E6%2596%2587%25E4%25BB%25B6\Dict\8.5.0.0\resultui\html\index.html#/javascript:;) | 1.096 (0.721-1.667) | 0.667 | 1.096 (0.721-1.667) | 0.667 | 0.794 |  | 1.163 (0.756-1.791) | 0.492 | 1.163 (0.756-1.791) | 0.492 | 0.820 |
| [Erythema](file:///D:\%25E6%2596%2587%25E4%25BB%25B6\Dict\8.5.0.0\resultui\html\index.html#/javascript:;) | 1.043 (0.687-1.584) | 0.844 | 1.042 (0.686-1.584) | 0.846 | 0.846 |  | 1.398 (0.904-2.162) | 0.133 | 1.410 (0.911-2.183) | 0.123 | 0.563 |
| [Alopecia](file:///D:\%25E6%2596%2587%25E4%25BB%25B6\Dict\8.5.0.0\resultui\html\index.html#/javascript:;) | 1.172 (0.746-1.842) | 0.492 | 1.175 (0.745-1.853) | 0.488 | 0.794 |  | 1.348 (0.859-2.115) | 0.193 | 1.376 (0.873-2.167) | 0.169 | 0.563 |
| [Canker](file:///D:\%25E6%2596%2587%25E4%25BB%25B6\Dict\8.5.0.0\resultui\html\index.html#/javascript:;) [sore](file:///D:\%25E6%2596%2587%25E4%25BB%25B6\Dict\8.5.0.0\resultui\html\index.html#/javascript:;) | 1.264 (0.729-2.191) | 0.404 | 1.264 (0.730-2.191) | 0.403 | 0.794 |  | 0.854 (0.487-1.497) | 0.581 | 0.852 (0.486-1.495) | 0.577 | 0.820 |
| P[leurisy](file:///D:\%25E6%2596%2587%25E4%25BB%25B6\Dict\8.5.0.0\resultui\html\index.html#/javascript:;) | 2.027 (0.825-4.976) | 0.123 | 2.028 (0.826-4.979) | 0.123 | 0.794 |  | 0.826 (0.367-1.861) | 0.645 | 0.831 (0.369-1.872) | 0.656 | 0.820 |
| Hematologic disorder | 0.812 (0.533-1.237) | 0.332 | 0.812 (0.533-1.237) | 0.332 | 0.794 |  | 1.178 (0.764-1.817) | 0.459 | 1.174 (0.761-1.812) | 0.468 | 0.820 |
| Anti-dsDNA | 0.925 (0.607-1.407) | 0.715 | 0.925 (0.607-1.408) | 0.715 | 0.794 |  | 0.975 (0.631-1.507) | 0.911 | 0.974 (0.631-1.506) | 0.907 | 0.967 |
| Low complement | 0.860 (0.562-1.315) | 0.486 | 0.858 (0.560-1.314) | 0.481 | 0.794 |  | 0.978 (0.633-1.511) | 0.919 | 0.991 (0.640-1.535) | 0.967 | 0.967 |
| *OR*, odds ratio; *CI*, confidence interval; *BH*, Benjamini-Hochberg method based on the false discovery rate;  **^*^**Adjusted for age | | | | | | | | | | | |

| **Table** **S14** Comparison of baseline characteristics of patients between GCs-sensitive and GCs-insensitive group | | | | |
| --- | --- | --- | --- | --- |
| **Characteristic** | **GCs-sensitive group (n=261)** | **GCs-insensitive group (n=192)** | **Overall (N=453)** | ***P* value** |
| Age, mean (SD) | 34.93 (12.36) | 35.26 (11.73) | 35.07 (12.09) | 0.772 |
| [Height](D:/%E6%96%87%E4%BB%B6/Dict/8.5.0.0/resultui/html/index.html#/javascript:;), mean (SD) | 160.66 (5.07) | 160.91 (4.88) | 160.76 (4.99) | 0.589 |
| [Weight](D:/%E6%96%87%E4%BB%B6/Dict/8.5.0.0/resultui/html/index.html#/javascript:;), mean (SD) | 54.31 (8.60) | 55.32 (8.51) | 54.74 (8.57) | 0.216 |
| Anxiety, no. (%) | 219 (83.91) | 154 (80.21) | 373 (82.34) | 0.308 |
| Depression, no. (%) | 223 (85.44) | 156 (81.25) | 379 (83.66) | 0.233 |
| Male, no. (%) | 24 (9.20) | 21 (10.94) | 45 (9.93) | 0.540 |
| Married, no. (%) | 197 (75.48) | 147 (76.56) | 344 (75.94) | 0.790 |
| Smoking, no. (%) | 14 (5.36) | 10 (5.21) | 24 (5.30) | 0.942 |
| Drinking, no. (%) | 23 (8.81) | 23 (11.98) | 46 (10.15) | 0.270 |
| Tea consumption, no. (%) | 87 (33.33) | 59 (30.73) | 146 (32.23) | 0.558 |
| History of GCs therapy, no. (%) | 61 (23.37) | 35 (18.23) | 96 (21.20) | 0.186 |
| SLEDAI scores, mean (SD) | 11.77 (3.00) | 11.49 (3.11) | 11.65 (3.05) | 0.333 |
| GCs dose mg/d, mean (SD) | 42.72 (15.46) | 40.94 (16.72) | 41.96 (16.01) | 0.242 |
| *SD*, standard deviation | | | | |

| **Table S15** Genotype frequency distribution of DYNC1H1 gene between GCs-sensitive and GCs-insensitive groups | | | | | | | | | | | | | | |
| --- | --- | --- | --- | --- | --- | --- | --- | --- | --- | --- | --- | --- | --- | --- |
| **Polymorphisms**  **(minor allele)** | **GCs-sensitive group (n=261)** | | |  | **GCs-insensitive group (n=192)** | | |  | **Overall (N=453)** | | |  | **HWE** | |
|  | **Wild** | **Heterozygous** | **Homozygous mutants** |  | **Wild** | **Heterozygous** | **Homozygous mutants** |  | **Wild** | **Heterozygous** | **Homozygous mutants** |  | **χ² value** | ***P* value** |
| rs1004903 (A) | 147 (56.32) | 89 (34.10) | 25 (9.58) |  | 107 (55.73) | 72 (37.50) | 13 (6.77) |  | 254 (56.07) | 161 (35.54) | 38 (8.39) |  | 2.901 | 0.089 |
| rs11160668 (A) | 156 (59.77) | 92 (35.25) | 13 (4.98) |  | 116(60.42) | 70 (36.46) | 6 (3.12) |  | 272 (60.05) | 162 (35.76) | 19 (4.19) |  | 0.705 | 0.401 |
| rs1190605 (C) | 128 (49.04) | 105 (40.23) | 28 (10.73) |  | 94 (48.96) | 79 (41.14) | 19 (9.90) |  | 222 (49.01) | 184 (40.62) | 47 (10.37) |  | 0.923 | 0.337 |
| rs1190606 (G) | 96 (36.78) | 129 (49.43) | 36 (13.79) |  | 71 (36.98) | 93 (48.44) | 28 (14.58) |  | 167 (36.86) | 222 (49.01) | 64 (14.13) |  | 0.510 | 0.475 |
| rs12161908 (C) | 235 (90.04) | 25 (9.58) | 1 (0.38) |  | 173 (90.11) | 17 (8.85) | 2 (1.04) |  | 408 (90.07) | 42 (9.27) | 3 (0.66) |  | 2.620 | 0.106 |
| rs2273440 (A) | 144 (55.17) | 92 (35.25) | 25 (9.58) |  | 107 (55.73) | 69 (35.94) | 16 (8.33) |  | 251 (55.41) | 161 (35.54) | 41 (9.05) |  | 4.055 | **0.044** |
| rs3818188 (A) | 72 (27.59) | 128 (49.04) | 61 (23.37) |  | 47 (24.48) | 99 (51.56) | 46 (23.96) |  | 119 (26.27) | 227 (50.11) | 107 (23.62) |  | 0.004 | 0.951 |
| *HWE*, Hardy-Weinberg equilibrium | | | | | | | | | | | | | | |

| **Table S16** Comparison of different alleles of DYNC1H1 in GCs-sensitive and GCs-insensitive groups | | | | | | |
| --- | --- | --- | --- | --- | --- | --- |
| **Allele** | **GCs-sensitive (N=261)**  **[n(%)]** | **GCs-insensitive (N=192)**  **[n(%)]** | ***χ*^2^ value** | ***OR* (*95% CI*)** | ***P* value** | ***P_BH_*** |
| rs1004903 |  |  | 0.140 | 0.944 (0.699-1.275) | 0.708 | 0.928 |
| G | 383 (73.37) | 286 (74.48) |  |  |  |  |
| A | 139 (26.63) | 98 (25.52) |  |  |  |  |
| rs11160668 |  |  | 0.201 | 0.930 (0.676-1.279) | 0.654 | 0.928 |
| G | 404 (77.39) | 302 (78.65) |  |  |  |  |
| A | 118 (22.61) | 82 (21.35) |  |  |  |  |
| rs1190605 |  |  | 0.015 | 0.983 (0.738-1.308) | 0.904 | 0.928 |
| G | 361 (69.16) | 267 (69.53) |  |  |  |  |
| C | 161 (30.84) | 117 (30.47) |  |  |  |  |
| rs1190606 |  |  | 0.008 | 1.013 (0.773-1.327) | 0.928 | 0.928 |
| A | 321 (61.49) | 235 (61.20) |  |  |  |  |
| G | 201 (38.51) | 149 (38.80) |  |  |  |  |
| rs12161908 |  |  | 0.039 | 1.060 (0.590-1.906) | 0.844 | 0.928 |
| G | 495 (94.83) | 363 (94.53) |  |  |  |  |
| C | 27 (5.17) | 21 (5.47) |  |  |  |  |
| rs3818188 |  |  | 0.302 | 1.077 (0.827-1.402) | 0.583 | 0.928 |
| G | 272 (52.11) | 193 (50.26) |  |  |  |  |
| A | 250 (47.89) | 191 (49.74) |  |  |  |  |
| *OR*, odds ratio; *CI*, confidence interval; *BH*, Benjamini-Hochberg method based on the false discovery rate | | | | | | |

| **Table S17** Comparison of different alleles of DYNC1H1 in GCs-sensitive and GCs-insensitive groups (male) | | | | | | |
| --- | --- | --- | --- | --- | --- | --- |
| **Allele** | **GCs-sensitive (N=24)**  **[n(%)]** | **GCs-insensitive (N=21)**  **[n(%)]** | ***χ*^2^ value** | ***OR* (*95% CI*)** | ***P* value** | ***P_BH_*** |
| rs1004903 |  |  | 1.437 | 0.570 (0.226-1.436) | 0.231 | 1.000 |
| G | 31 (64.58) | 32 (76.19) |  |  |  |  |
| A | 17 (35.42) | 10 (23.81) |  |  |  |  |
| rs11160668 |  |  | 0.396 | 1.345 (0.534-3.390) | 0.529 | 1.000 |
| G | 36 (75.00) | 29 (69.05) |  |  |  |  |
| A | 12 (25.00) | 13 (30.95) |  |  |  |  |
| rs1190605 |  |  | 0.000 | 1.000 (0.438-2.285) | 1.000 | 1.000 |
| G | 30 (62.50) | 30 (64.29) |  |  |  |  |
| C | 18 (37.50) | 18 (35.71) |  |  |  |  |
| rs1190606 |  |  | 0.031 | 0.926 (0.392-2.188) | 0.861 | 1.000 |
| A | 30 (62.50) | 27 (64.29) |  |  |  |  |
| G | 18 (37.50) | 15 (35.71) |  |  |  |  |
| rs12161908 |  |  | 0.014 | 2.350 (0.205-26.886) | 0.906 | 1.000 |
| G | 47 (97.92) | 40 (95.24) |  |  |  |  |
| C | 1 (2.08) | 2 (4.76) |  |  |  |  |
| rs3818188 |  |  | 0.083 | 1.133 (0.485-2.649) | 0.773 | 1.000 |
| G | 30 (62.50) | 25 (59.22) |  |  |  |  |
| A | 18 (37.50) | 17 (40.48) |  |  |  |  |
| *OR*, odds ratio; *CI*, confidence interval; *BH*, Benjamini-Hochberg method based on the false discovery rate | | | | | | |

| **Table S18** Comparison of different alleles of DYNC1H1 in GCs-sensitive and GCs-insensitive groups (female) | | | | | | |
| --- | --- | --- | --- | --- | --- | --- |
| **Allele** | **GCs-sensitive (N=237)**  **[n(%)]** | **GCs-insensitive (N=171)**  **[n(%)]** | ***χ*^2^ value** | ***OR* (*95% CI*)** | ***P* value** | ***P_BH_*** |
| rs1004903 |  |  | 0.001 | 1.000 (0.727-1.374) | 0.998 | 0.998 |
| G | 352 (74.26) | 254 (74.27) |  |  |  |  |
| A | 122 (25.74) | 88 (25.73) |  |  |  |  |
| rs11160668 |  |  | 0.564 | 0.877 (0.624-1.234) | 0.453 | 0.998 |
| G | 368 (77.64) | 273 (79.82) |  |  |  |  |
| A | 106 (22.36) | 69 (20.18) |  |  |  |  |
| rs1190605 |  |  | 0.027 | 1.025 (0.758-1.387) | 0.870 | 0.998 |
| G | 331 (69.83) | 237 (69.30) |  |  |  |  |
| C | 143 (30.17) | 105 (30.70) |  |  |  |  |
| rs1190606 |  |  | 0.028 | 1.024 (0.770-1.362) | 0.868 | 0.998 |
| A | 291 (61.39) | 208 (60.82) |  |  |  |  |
| G | 183 (38.61) | 134 (39.18) |  |  |  |  |
| rs12161908 |  |  | 0.002 | 1.014 (0.551-1.863) | 0.965 | 0.998 |
| G | 448 (94.51) | 323 (94.44) |  |  |  |  |
| C | 26 (5.49) | 19 (5.56) |  |  |  |  |
| rs3818188 |  |  | 0.297 | 1.080 (0.818-1.427) | 0.586 | 0.998 |
| G | 242 (51.05) | 168 (49.12) |  |  |  |  |
| A | 232 (48.95) | 174 (50.88) |  |  |  |  |
| *OR*, odds ratio; *CI*, confidence interval; *BH*, Benjamini-Hochberg method based on the false discovery rate | | | | | | |

| **Table S19** Association between DYNC1N1 polymorphisms and GCs efficacy in followed up patients (male) | | | | | | | | | | | |
| --- | --- | --- | --- | --- | --- | --- | --- | --- | --- | --- | --- |
| **Polymorphisms** | **Dominant model** | | | | |  | **Recessive model** | | | | |
|  | **Crude *OR* (*95% CI*)** | **Crude**  ***P* value** | **Adjusted^*^**  ***OR* (*95% CI*)** | **Adjusted** ***P* value** | ***P_BH_*** |  | **Crude *OR* (*95% CI*)** | **Crude**  ***P* value** | **Adjusted^*^**  ***OR* (*95% CI*)** | **Adjusted**  ***P* value** | ***P_BH_*** |
| rs1004903 | 0.647 (0.200-2.113) | 0.473 | 0.832 (0.186-3.723) | 0.810 | 0.810 |  | − | − | − | − | − |
| rs11160668 | 1.540 (0.473-5.011) | 0.473 | 2.664 (0.488-14.540) | 0.258 | 0.810 |  | 1.158 (0.148-9.029) | 0.889 | 0.912 (0.070-11.884) | 0.944 | 0.995 |
| rs1190605 | 0.800 (0.242-2.645) | 0.715 | 1.662 (0.308-8.969) | 0.555 | 0.810 |  | − | − | − | − | − |
| rs1190606 | 0.660 (0.201-2.170) | 0.494 | 0.827 (0.183-3.739) | 0.805 | 0.810 |  | 1.647 (0.323-8.388) | 0.548 | 1.008 (0.087-11.636) | 0.995 | 0.995 |
| rs12161908 | 2.421 (0.204-28.800) | 0.484 | 6.642 (0.214-206.226) | 0.280 | 0.810 |  | − | − | − | − | − |
| rs3818188 | 1.920 (0.523-7.029) | 0.326 | 1.548 (0.300-7.987) | 0.602 | 0.810 |  | 0.350 (0.034-3.650) | 0.380 | 0.021 (0.001-0.846) | **0.041** | 0.123 |
| *OR*, odds ratio; *CI*, confidence interval; *BH*, Benjamini-Hochberg method based on the false discovery rate;  **^*^**Adjusted for age, height, weight, marital status, smoking, drinking, tea consumption, history of GCs therapy, GCs dose, and SLEDAI scores at baseline | | | | | | | | | | | |

| **Table S20** Association between DYNC1N1 polymorphisms and GCs efficacy in followed up patients (female) | | | | | | | | | | | |
| --- | --- | --- | --- | --- | --- | --- | --- | --- | --- | --- | --- |
| **Polymorphisms** | **Dominant model** | | | | |  | **Recessive model** | | | | |
|  | **Crude *OR* (*95% CI*)** | **Crude**  ***P* value** | **Adjusted^*^**  ***OR* (*95% CI*)** | **Adjusted** ***P* value** | ***P_BH_*** |  | **Crude *OR* (*95% CI*)** | **Crude**  ***P* value** | **Adjusted^*^**  ***OR* (*95% CI*)** | **Adjusted**  ***P* value** | ***P_BH_*** |
| rs1004903 | 1.070 (0.720-1.592) | 0.737 | 1.034 (0.688-1.555) | 0.871 | 0.957 |  | 0.804 (0.393-1.645) | 0.550 | 0.812 (0.389-1.697) | 0.580 | 0.859 |
| rs11160668 | 0.917 (0.612-1.372) | 0.672 | 0.921 (0.608-1.394) | 0.696 | 0.957 |  | 0.492 (0.154-1.572) | 0.232 | 0.557 (0.170-1.820) | 0.333 | 0.859 |
| rs1190605 | 1.020 (0.689-1.512) | 0.920 | 0.968 (0.645-1.453) | 0.876 | 0.957 |  | 1.060 (0.564-1.994) | 0.857 | 1.065 (0.557-2.038) | 0.849 | 0.859 |
| rs1190606 | 1.046 (0.694-1.575) | 0.830 | 0.989 (0.649-1.505) | 0.957 | 0.957 |  | 1.009 (0.573-1.779) | 0.975 | 1.055 (0.588-1.891) | 0.859 | 0.859 |
| rs12161908 | 0.936 (0.489-1.793) | 0842 | 0.942 (0.484-1.834) | 0.861 | 0.957 |  | 2.793 (0.251-31.052) | 0.403 | 2.549 (0.224-28.982) | 0.451 | 0.859 |
| rs3818188 | 1.112 (0.708-1.748) | 0.645 | 1.051 (0.660-1.672) | 0.835 | 0.957 |  | 1.102 (0.702-1.731) | 0.672 | 1.187 (0.742-1.897) | 0.475 | 0.859 |
| *OR*, odds ratio; *CI*, confidence interval; *BH*, Benjamini-Hochberg method based on the false discovery rate;  **^*^**Adjusted for age, height, weight, marital status, smoking, drinking, tea consumption, history of GCs therapy, GCs dose, and SLEDAI scores at baseline | | | | | | | | | | | |

| **Table S21** Association between haplotypes of DYNC1H1 gene and GCs efficacy | | | | | | | |
| --- | --- | --- | --- | --- | --- | --- | --- |
| **Haplotypes** | **GCs-sensitive**  **(frequencies)** | **GCs-insensitive**  **(frequencies)** | ***χ*² value** | **Fisher’s *P* value** | **Pearson’s *P* value** | ***OR* (*95% CI*)** | ***P_BH_*** |
| A G C G G G | 85.88 (0.224) | 121.61 (0.233) | 0.105 | 0.746 | 0.746 | 0.949 (0.691-1.303) | 0.754 |
| G A G A G G | 65.60 (0.171) | 85.20 (0.163) | 0.098 | 0.754 | 0.754 | 1.058 (0.742-1.509) | 0.754 |
| G A G G G G | 9.05 (0.024) | 23.40 (0.045) | 2.896 | 0.089 | 0.089 | 0.514 (0.236-1.121) | 0.345 |
| G G C A C G | 17.99 (0.047) | 21.99 (0.042) | 0.120 | 0.729 | 0.729 | 1.119 (0.591-2.119) | 0.754 |
| G G G A G A | 143.94 (0.375) | 202.18 (0.387) | 0.142 | 0.706 | 0.706 | 0.948 (0.719-1.250) | 0.754 |
| G G G G G A | 40.18 (0.105) | 39.09 (0.075) | 2.481 | 0.115 | 0.115 | 1.448 (0.912-2.301) | 0.345 |
| *OR*, odds ratio; *CI*, confidence interval; *BH*, Benjamini-Hochberg method based on the false discovery rate;  frequency < 0.03 in both groups has been dropped. | | | | | | | |

| **Table S22** Association between haplotypes of DYNC1H1 gene and GCs efficacy (male) | | | | | | | |
| --- | --- | --- | --- | --- | --- | --- | --- |
| **Haplotypes** | **GCs-sensitive**  **(frequencies)** | **GCs-insensitive**  **(frequencies)** | ***χ*² value** | **Fisher’s *P* value** | **Pearson’s *P* value** | ***OR* (*95% CI*)** | ***P_BH_*** |
| A G C G G G | 10.00 (0.238) | 15.97 (0.333) | 1.264 | 0.261 | 0.261 | 0.585 (0.229-1.496) | 0.546 |
| G A G A G G | 12.00 (0.286) | 9.98 (0.208) | 0.564 | 0.453 | 0.453 | 1.450 (0.548-3.839) | 0.546 |
| G G C A C G | 2.00 (0.048) | 1.00 (0.021) | 0.365 | 0.546 | 0.546 | 2.254 (0.285-17.846) | 0.546 |
| G G G A G A | 13.00 (0.310) | 18.00 (0.375) | 0.645 | 0.422 | 0.422 | 0.695 (0.286-1.690) | 0.546 |
| G G G G G A | 4.00 (0.095) | 0.00 (0.000) | 3.789 | 0.052 | 0.052 | 3374.524 (176.643-64465.645) | 0.260 |
| *OR*, odds ratio; *CI*, confidence interval; *BH*, Benjamini-Hochberg method based on the false discovery rate;  frequency < 0.03 in both groups has been dropped. | | | | | | | |

| **Table S23** Association between haplotypes of DYNC1H1 gene and GCs efficacy (female) | | | | | | | |
| --- | --- | --- | --- | --- | --- | --- | --- |
| **Haplotypes** | **GCs-sensitive**  **(frequencies)** | **GCs-insensitive**  **(frequencies)** | ***χ*² value** | **Fisher’s *P* value** | **Pearson’s *P* value** | ***OR* (*95% CI*)** | ***P_BH_*** |
| A G C G G G | 75.83 (0.222) | 105.59 (0.223) | 0.000 | 0.985 | 0.985 | 1.003 (0.716-1.406) | 0.985 |
| G A G A G G | 52.27 (0.153) | 73.99 (0.156) | 0.007 | 0.932 | 0.932 | 0.983 (0.667-1.448) | 0.985 |
| G A G G G G | 8.98 (0.026) | 23.07 (0.049) | 2.588 | 0.108 | 0.108 | 0.530 (0.242-1.162) | 0.648 |
| G G C A C G | 15.99 (0.047) | 20.99 (0.044) | 0.036 | 0.850 | 0.850 | 1.066 (0.547-2.078) | 0.985 |
| G G G A G A | 132.30 (0.387) | 185.39 (0.391) | 0.002 | 0.964 | 0.964 | 0.993 (0.742-1.329) | 0.985 |
| G G G G G A | 35.22 (0.103) | 38.45 (0.081) | 1.236 | 0.266 | 0.266 | 1.313 (0.811-2.126) | 0.798 |
| *OR*, odds ratio; *CI*, confidence interval; *BH*, Benjamini-Hochberg method based on the false discovery rate;  frequency < 0.03 in both groups has been dropped. | | | | | | | |

| **Table S24** Comparison of improvement in anxiety and depression of patients among different genotypes | | | | | | | | | | | |
| --- | --- | --- | --- | --- | --- | --- | --- | --- | --- | --- | --- |
| **Disease improvement** | **Polymorphisms** | **Dominant model** | | | |  | **Recessive model** | | | | |
|  |  | **Wild** | **Heterozygous/**  **Homozygous mutants** | ***P* value** | ***P_BH_*** |  | **Wild/**  **Heterozygous** | | **Homozygous mutants** | ***P* value** | ***P_BH_*** |
| Anxiety |  |  |  |  |  |  |  | |  |  |  |
|  | rs1004903 | 2.00 (0-5.00) | 2.00 (0-4.00) | 0.486 | 0.916 |  | 2.00 (0-5.00) | | 1.50 (0-3.00) | 0.424 | 0.647 |
|  | rs11160668 | 2.00 (0-5.00) | 2.00 (0-5.00) | 0.353 | 0.916 |  | 2.00 (0-5.00) | | 3.00 (-1.00-6.00) | 0.431 | 0.647 |
|  | rs1190605 | 2.00 (0-5.00) | 2.00 (0-4.00) | 0.701 | 0.916 |  | 2.00 (0-5.00) | | 2.00 (0-5.00) | 0.996 | 0.996 |
|  | rs1190606 | 2.00 (0-5.00) | 2.00 (0-5.00) | 0.916 | 0.916 |  | 2.00 (0-5.00) | | 2.00 (-0.50-4.00) | 0.607 | 0.728 |
|  | rs12161908 | 2.00 (0-5.00) | 3.00 (0-5.00) | 0.407 | 0.916 |  | 2.00 (0-5.00) | | 5.00 (1.00-5.00) | 0.403 | 0.647 |
|  | rs3818188 | 2.00 (-1.00-5.00) | 2.00 (0-5.00) | 0.848 | 0.916 |  | 2.00 (0-5.00) | | 2.00 (0-5.00) | 0.314 | 0.647 |
| Depression |  |  |  |  |  |  |  |  | |  |  |
|  | rs1004903 | 4.00 (0-9.00) | 4.00 (0-7.00) | 0.724 | 0.961 |  | 4.00 (0-8.00) | 4.50 (3.00-7.00) | | 0.388 | 0.548 |
|  | rs11160668 | 4.00 (0-8.00) | 3.00 (0-7.00) | 0.336 | 0.961 |  | 4.00 (0-7.00) | 6.00 (-1.00-13.00) | | 0.429 | 0.548 |
|  | rs1190605 | 4.00 (0-9.00) | 4.00 (0-7.00) | 0.776 | 0.961 |  | 4.00 (0-8.00) | 5.00 (2.00-7.00) | | 0.457 | 0.548 |
|  | rs1190606 | 4.00 (0-8.00) | 4.00 (0-8.00) | 0.961 | 0.961 |  | 4.00 (0-7.00) | 4.00 (2.00-8.50) | | 0.225 | 0.548 |
|  | rs12161908 | 4.00 (0-8.00) | 4.00 (1.0-7.00) | 0.717 | 0.961 |  | 4.00 (0-8.00) | 7.00 (3.00-10.00) | | 0.354 | 0.548 |
|  | rs3818188 | 4.00 (0-7.00) | 4.00 (0-8.00) | 0.886 | 0.961 |  | 4.00 (0-7.00) | 4.00 (0-9.00) | | 0.559 | 0.559 |
| *BH*, Benjamini-Hochberg method based on the false discovery rate; | | | | | | | | | | | |

| **Table S25** Comparison of improvement in health-related quality of life of patients among different genotypes | | | | | | | | | |
| --- | --- | --- | --- | --- | --- | --- | --- | --- | --- |
| **Polymorphisms** | **Dominant model** | | | |  | **Recessive model** | | | |
|  | **Wild** | **Heterozygous/**  **Homozygous mutants** | ***P* value** | ***P_BH_*** |  | **Wild/**  **Heterozygous** | **Homozygous mutants** | ***P* value** | ***P_BH_*** |
| rs1004903, no. (%) | 254 (56.07) | 199 (43.93) |  |  |  | 415 (91.61) | 38 (8.39) |  |  |
| Total score | 4.16 (-0.94-13.44) | 4.06 (-0.69-11.06) | 0.825 | 0.978 |  | 4.63 (-0.69-13.00) | 1.50 (-1.13-9.31) | 0.202 | 0.647 |
| Physical function | 5.00 (0-20.00) | 5.00 (0-20.00) | 0.954 | 0.978 |  | 5.00 (0-25.00) | 5.00 (0-15.00) | 0.393 | 0.647 |
| Role-physical | 0 (0-0) | 0 (0-0) | 0.884 | 0.978 |  | 0 (0-0) | 0 (0-0) | 0.409 | 0.647 |
| Bodily pain | 10.00 (0-26.00) | 2.00 (0-22.00) | 0.370 | 0.978 |  | 9.00 (0-23.00) | 0 (0-22.00) | 0.331 | 0.647 |
| General health | 0 (-5.00-10.00) | 0 (-5.00-5.00) | 0.410 | 0.978 |  | 0 (-5.00-5.00) | 0 (-10.00-5.00) | 0.277 | 0.647 |
| Vitality | 0 (-5.00-10.00) | 0 (-5.00-10.00) | 0.978 | 0.978 |  | 0 (-5.00-10.00) | 2.50 (0-10.00) | 0.864 | 0.864 |
| Social function | 0 (0-12.50) | 0 (0-12.50) | 0.588 | 0.978 |  | 0 (0-12.50) | 0 (0-12.50) | 0.718 | 0.835 |
| Role-emotional | 0 (0-0) | 0 (0-0) | 0.875 | 0.978 |  | 0 (0-0) | 0 (0-0) | 0.693 | 0.835 |
| Mental health | 0 (-4.00-8.00) | 0 (-4.00-4.00) | 0.132 | 0.978 |  | 0 (-4.00-4.00) | -4.00 (-4.00-8.00) | 0.412 | 0.647 |
| PCS | 5.50 (-0.25-17.00) | 5.00 (0-15.50) | 0.558 | 0.978 |  | 5.25 (0-17.00) | 2.63 (-3.75-13.00) | 0.105 | 0.647 |
| MCS | 2.31 (-1.88-10.58) | 2.38 (-1.50-10.75) | 0.754 | 0.978 |  | 2.50 (-1.63-10.58) | 2.13 (-1.63-6.38) | 0.759 | 0.835 |
| rs11160668, no. (%) | 272 (60.04) | 181 (39.96) |  |  |  | 434 (95.81) | 19 (4.19) |  |  |
| Total score | 3.22 (-0.59-10.47) | 5.25 ( -1.13- 14.38) | 0.318 | 0.500 |  | 4.31 (-1.00-12.81) | 3.50 (0.88-6.88) | 0.819 | 0.993 |
| Physical function | 5.00 (0-25.00) | 5.00 (0-20.00) | 0.420 | 0.513 |  | 5.00 (0-20.00) | 0 (-5.00-20.00) | 0.396 | 0.993 |
| Role-physical | 0 (0-0) | 0 (0-25.00) | **0.047** | 0.259 |  | 0 (0-0) | 0 (0-0) | 0.871 | 0.993 |
| Bodily pain | 7.50 (0-22.00) | 4.00 (0-23.00) | 0.898 | 0.898 |  | 6.00 (0-22.00) | 10.00 (0-22.00) | 0.993 | 0.993 |
| General health | 0 (-5.00-5.00) | 0 (-5.00-10.00) | 0.618 | 0.680 |  | 0 (-5.00-5.00) | 0 (-10.00-10.00) | 0.418 | 0.993 |
| Vitality | 0 (-5.00-10.00) | 0 (-5.00-10.00) | 0.270 | 0.500 |  | 0 (-5.00-10.00) | 0 (-5.00-5.00) | 0.328 | 0.993 |
| Social function | 0 (0-12.50) | 0 (0-12.50) | 0.370 | 0.509 |  | 0 (0-12.50) | 12.50 (0-12.50) | 0.805 | 0.993 |
| Role-emotional | 0 (0-0) | 0 (0-0) | **0.016** | 0.176 |  | 0 (0-0) | 0 (0-0) | 0.899 | 0.993 |
| Mental health | 0 (-4-8.00) | 0 (-4.00-4.00) | 0.279 | 0.500 |  | 0 (-4.00-4.00) | 0 (-8.00-0) | 0.160 | 0.993 |
| PCS | 5.00 (-0.13-14.75) | 6.25 (0-20.00) | 0.265 | 0.500 |  | 5.13 (0-17.00) | 5.00 (-0.75-13.50) | 0.888 | 0.993 |
| MCS | 2.13 (-1.25-9.00) | 2.88 (-2.50-11.88) | 0.298 | 0.500 |  | 2.38 (-1.88-10.75) | 2.13 (0-4.38) | 0.910 | 0.993 |
| rs1190605, no. (%) | 222 (49.01) | 231 (50.99) |  |  |  | 406 (89.62) | 47 (10.38) |  |  |
| Total score | 4.06 (-0.94-13.50) | 4.38 (-0.69-11.29) | 0.896 | 0.939 |  | 4.59 (-0.69-13.17) | 1.81 (-1.75-9.31) | 0.091 | 0.334 |
| Physical function | 5.00 (0-25.00) | 5.00 (0-20.00) | 0.520 | 0.939 |  | 5.00 (0-25.00) | 0 (0-10.00) | **0.030** | 0.253 |
| Role-physical | 0 (0-0) | 0 (0-0) | 0.806 | 0.939 |  | 0 (0-0) | 0 (0-0) | 0.241 | 0.404 |
| Bodily pain | 10.00 (0-26.00) | 4.00 (0-22.00) | 0.806 | 0.939 |  | 9.50 (0-22.00) | 0 (0-22.00) | 0.419 | 0.461 |
| General health | 0 (-5.00-10.00) | 0 (-5.00-5.00) | 0.529 | 0.939 |  | 0 (-5.00-5.00) | 0 (-10.00-5.00) | 0.161 | 0.404 |
| Vitality | 0 (-5.00-10.00) | 0 (-5.00-10.00) | 0.939 | 0.939 |  | 0 (-5.00-10.00) | 0 (-5.00-10.00) | 0.780 | 0.780 |
| Social function | 0 (0-12.50) | 0 (0-12.50) | 0.605 | 0.939 |  | 0 (0-12.50) | 0 (0-12.50) | 0.257 | 0.404 |
| Role-emotional | 0 (0-0) | 0 (0-0) | 0.929 | 0.939 |  | 0 (0-0) | 0 (0-0) | 0.309 | 0.425 |
| Mental health | 0 (-4.00-8.00) | 0 (-4.00-4.00) | 0.349 | 0.939 |  | 0 (-4.00-4.00) | -4.00 (-4.00-4.00) | 0.223 | 0.404 |
| PCS | 5.13 (-0.50-17.00) | 5.00 (0-16.25) | 0.633 | 0.939 |  | 5.50 (0-17.50) | 1.50 (-3.75-13.00) | **0.046** | 0.253 |
| MCS | 2.31 (-2.13-10.58) | 2.38 (-1.25-10.38) | 0.682 | 0.939 |  | 2.50 (-1.25-10.58) | 1.50 (-2.25-6.38) | 0.378 | 0.461 |
| rs1190606, no. (%) | 167 (36.87) | 286 (63.13) |  |  |  | 389 (85.87) | 64 (14.13) |  |  |
| Total score | 3.31 (-2.00-13.50) | 4.63 (-0.31-11.81) | 0.606 | 0.984 |  | 4.25 (-1.00-13.17) | 3.56 (-0.03-10.60) | 0.988 | 0.988 |
| Physical function | 5.00 (0-20.00) | 5.00 (0-25.00) | 0.247 | 0.861 |  | 5.00 (0-25.00) | 5.00 (0-20.00) | 0.821 | 0.988 |
| Role-physical | 0 (0-0) | 0 (0-0) | 0.792 | 0.984 |  | 0 (0-0) | 0 (0-0) | 0.493 | 0.775 |
| Bodily pain | 10.00 (0-26.00) | 5.00 (0-22.00) | 0.984 | 0.984 |  | 10.00 (0-23.00) | 0 (0-22.00) | 0.355 | 0.775 |
| General health | 0 (-5.00-5.00) | 0 (-5.00-10.00) | 0.927 | 0.984 |  | 0 (-5.00-5.00) | 0 (-5.00-15.00) | 0.900 | 0.988 |
| Vitality | 0 (-5.00-10.00) | 0 (-5.0 0- 10.00) | 0.281 | 0.861 |  | 0 (-5.00-10.00) | 5.00 (-5.00-10.00) | 0.341 | 0.775 |
| Social function | 0 (0-12.50) | 0 (0-12.50) | **0.034** | 0.374 |  | 0 (0-12.50) | 12.50 (0-12.50) | 0.433 | 0.775 |
| Role-emotional | 0 (0-0) | 0 (0-0) | 0.937 | 0.984 |  | 0 (0-0) | 0 (0-0) | 0.613 | 0.843 |
| Mental health | 0 (-4.00-4.00) | 0 (-4.00-4.00) | 0.423 | 0.931 |  | 0 (-4.00-4.00) | 0 (-4.00-8.00) | 0.343 | 0.775 |
| PCS | 5.00 (-1.25-19.25) | 5.38 (0-15.50) | 0.874 | 0.984 |  | 5.25 (0-17.50) | 4.50 (-1.25-12.50) | 0.350 | 0.775 |
| MCS | 2.13 (-2.13-10.38) | 2.56 (-1.25-10.88) | 0.313 | 0.861 |  | 2.13 (-2.00-10.38) | 3.81 (-0.88-10.81) | 0.286 | 0.775 |
| rs12161908, no. (%) | 408 (90.07) | 45 (9.93) |  |  |  | 450 (99.34) | 3 (0.66) |  |  |
| Total score | 4.06 (-0.56-12.28) | 5.79 (-2.19-11.63) | 0.836 | 0.966 |  | 4.06 (-0.94-12.00) | 11.63 (8.00-31.13) | 0.125 | 0.344 |
| Physical function | 5.00 (0-25.00) | 5.00 (0-15.00) | 0.275 | 0.966 |  | 5.00 (0-20.00) | 25.00 (10-40.00) | 0.122 | 0.344 |
| Role-physical | 0 (0-0) | 0 (0-0) | 0.878 | 0.966 |  | 0 (0-0) | 0 (0-100.00) | 0.460 | 0.633 |
| Bodily pain | 6.00 (0-22.00) | 10.00 (0-26.00) | 0.668 | 0.966 |  | 6.00 (0-22.00) | 38.00 (-14.00-48.00) | 0.381 | 0.633 |
| General health | 0 (-5.00-5.00) | 0 (0-5.00) | 0.838 | 0.966 |  | 0 (-5.00-5.00) | 5.00 (0-15.00) | 0.404 | 0.633 |
| Vitality | 0 (-5.00-10.00) | 0 (-5.00-10.00) | 0.978 | 0.978 |  | 0 (-5.00-10.00) | -5.00 (-10.00-0) | 0.114 | 0.344 |
| Social function | 0 (0-12.50) | 0 (0-12.50) | 0.714 | 0.966 |  | 0 (0-12.50) | 0 (0-50.00) | 0.660 | 0.807 |
| Role-emotional | 0 (0-0) | 0 (0-0) | 0.601 | 0.966 |  | 0 (0-0) | 0 (0-100.00) | 0.432 | 0.633 |
| Mental health | 0 (-4.00-4.00) | 0 (0-4.00) | 0.218 | 0.966 |  | 0 (-4.00-4.00) | 0 (-4.00-8.00) | 0.790 | 0.869 |
| PCS | 5.13 (-0.25-16.50) | 3.75 (0-19.50) | 0.816 | 0.966 |  | 5.00 (-0.25-16.25) | 23.25 (19.50-24.00) | 0.058 | 0.344 |
| MCS | 2.38 (-1.38-10.48) | 0.88 (-2.25-10.88) | 0.796 | 0.966 |  | 2.38 (-1.63-10.58) | 0 (-3.50-38.25) | 0.961 | 0.961 |
| rs3818188, no. (%) | 119 (26.27) | 334 (73.73) |  |  |  | 346 (76.38) | 107 (23.62) |  |  |
| Total score | 3.88 (-0.63-12.56) | 4.38 (-0.94-12.00) | 0.790 | 0.810 |  | 4.59 (-0.94-11.94) | 3.19 (-0.81-13.50) | 0.794 | 0.888 |
| Physical function | 5.00 (0-20.00) | 5.00 ( 0- 25.00) | 0.158 | 0.515 |  | 5.00 (0-20.00) | 10.00 (0-25.00) | 0.138 | 0.888 |
| Role-physical | 0 (0-0) | 0 (0-0) | 0.234 | 0.515 |  | 0 (0-0) | 0 (0-0) | 0.349 | 0.888 |
| Bodily pain | 0 (0-22.00) | 10.00 (0-23.00) | 0.284 | 0.521 |  | 9.00 (0-22.00) | 4.00 (0-23.00) | 0.969 | 0.969 |
| General health | 0 (-5.00-10.00) | 0 (-5.00-5.00) | 0.096 | 0.515 |  | 0 (-5.00-5.00) | 0 (-5.00-5.00) | 0.735 | 0.888 |
| Vitality | 0 (-5.00-10.00) | 0 (-5.00-10.00) | 0.810 | 0.810 |  | 0 (-5.00-10.00) | 0 (-5.00-10.00) | 0.548 | 0.888 |
| Social function | 0 (0-12.50) | 0 (0-12.50) | 0.518 | 0.810 |  | 0 (0-12.50) | 0 (0-12.50) | 0.440 | 0.888 |
| Role-emotional | 0 (0-0) | 0 (0-0) | 0.225 | 0.515 |  | 0 (0-0) | 0 (0-0) | 0.677 | 0.888 |
| Mental health | 0 (-4.00-4.00) | 0 (-4.00-4.00) | 0.153 | 0.515 |  | 0 (-4.00-4.00) | 0 (-4.00-8.00) | 0.221 | 0.888 |
| PCS | 5.00 (-1.25-14.75) | 5.50 (0-17.00) | 0.480 | 0.810 |  | 5.38 (0-16.75) | 5.00 (-0.75-17.00) | 0.807 | 0.888 |
| MCS | 2.13 (-1.63-12.13) | 2.50 (-1.63-9.88) | 0.574 | 0.810 |  | 2.56 (-1.63-10.75) | 2.00 (-1.25-10.25) | 0.769 | 0.888 |
| *BH*, Benjamini-Hochberg method based on the false discovery rate; | | | | | | | | | |

| **Table S26** Comparison of improvement in health related quality of life of patients among different genotypes (male) | | | | | | | | | |
| --- | --- | --- | --- | --- | --- | --- | --- | --- | --- |
| **Polymorphisms** | **Dominant model** | | | |  | **Recessive model** | | | |
|  | **Wild** | **Heterozygous/**  **Homozygous mutants** | ***P* value** | ***P_BH_*** |  | **Wild/**  **Heterozygous** | **Homozygous mutants** | ***P* value** | ***P_BH_*** |
| rs1004903, no. (%) | 21 (46.67) | 24 (53.33) |  |  |  | 42 (93.33) | 3 (6.67) |  |  |
| Total score | 3.19 (-0.94-6.88) | 5.00 (-0.44-10.56) | 0.251 | 0.385 |  | 3.78 (-0.31-9.75) | 0.44 (-3.13-40.44) | 0.856 | 0.964 |
| Physical function | 0 (-5.00-20.00) | 5.00 (0-20.00) | 0.101 | 0.311 |  | 0 (-5.00-15.00) | 20.00 (5.00-20.00) | 0.176 | 0.964 |
| Role-physical | 0 (0-0) | 0 (0-0) | 0.649 | 0.714 |  | 0 (0-0) | 0 (0-0) | 0.433 | 0.964 |
| Bodily pain | 0 (0-16.00) | 10.50 (0-24.50) | 0.113 | 0.311 |  | 6.50 (0-20.00) | 10.00 (-26.00-52.00) | 0.963 | 0.964 |
| General health | 0 (-5.00-10.00) | 0 (-5.00-17.50) | 0.720 | 0.720 |  | 0 (-5.00-15.00) | 0 (-10.00-20.00) | 0.908 | 0.964 |
| Vitality | 0 (-5.00-0) | 5.00 (-5.00-15.00) | 0.041 | 0.226 |  | 0 (-5.00-10.00) | 5.00 (-5.00-15.00) | 0.549 | 0.964 |
| Social function | 0 (0-12.50) | 12.50 (0-12.50) | 0.253 | 0.385 |  | 0 (0-12.50) | 0 (-12.50-12.50) | 0.475 | 0.964 |
| Role-emotional | 0 (0-0) | 0 (0-0) | 0.628 | 0.714 |  | 0 (0-0) | 0 (0-0) | 0.398 | 0.964 |
| Mental health | 0 (-8.00-0) | 4.00 (-2.00-8.00) | **0.012** | 0.132 |  | 0 (-4.00-8.00) | -4.00 (-4.00-4.00) | 0.563 | 0.964 |
| PCS | 1.75 (-1.75-12.50) | 6.50 (2.34-11.13) | 0.280 | 0.385 |  | 5.00 (0-12.00) | 3.75 (-4.00-48.00) | 0.964 | 0.964 |
| MCS | 2.00 (-4.50-4.38) | 3.25 (-0.88-12.63) | 0.169 | 0.372 |  | 2.38 (-1.50-9.00) | -2.25 (-2.88-32.88) | 0.767 | 0.964 |
| rs11160668, no. (%) | 24 (53.33) | 21 (46.67) |  |  |  | 41 (91.11) | 4 (8.89) |  |  |
| Total score | 4.09 (1.28-9.44) | 3.50 (-0.63-9.81) | 0.577 | 0.705 |  | 4.06 (-0.63-9.81) | 2.19 (0.28-5.19) | 0.675 | 0.888 |
| Physical function | 5.00 (-2.50-17.50) | 0 (-5.00-10.00) | 0.714 | 0.785 |  | 5.00 (-5.00-15.00) | 5.00 (-5.00-20.00) | 0.888 | 0.888 |
| Role-physical | 0 (0-0) | 0 (0-0) | 0.442 | 0.705 |  | 0 (0-0) | 0 (0-0) | 0.527 | 0.888 |
| Bodily pain | 16.00 (6.50-27.00) | 0 (0-0) | **0.001** | **0.011** |  | 10.00 (0-20.00) | 0 (-3.00-19.50) | 0.475 | 0.888 |
| General health | 5.00 (-2.50-17.50) | 0 (-5.00-0) | 0.087 | 0.479 |  | 0 (-5.00-15.00) | 2.50 (-7.50-12.50) | 0.823 | 0.888 |
| Vitality | 2.50 (-5.00-15.00) | 0 (-5.00-0) | 0.173 | 0.634 |  | 0 (-5.00-10.00) | 2.50 (-5.00-10.00) | 0.808 | 0.888 |
| Social function | 12.50 (0-12.50) | 0 (0-12.50) | 0.505 | 0.705 |  | 0 (0-12.50) | 0 (0-6.25) | 0.677 | 0.888 |
| Role-emotional | 0 (0-0) | 0 (0-0) | 0.443 | 0.705 |  | 0 (0-0) | 0 (0-0) | 0.528 | 0.888 |
| Mental health | 0 (-4.00-4.00) | 0 (-4.00-8.00) | 0.532 | 0.705 |  | 0 (-4.00-4.00) | 0 (-8.00-4.00) | 0.792 | 0.888 |
| PCS | 8.38 (2.75-11.13) | 1.75 (-1.75-12.50) | 0.333 | 0.705 |  | 5.00 (0-12.00) | 4.88 (-2.63-11.75) | 0.750 | 0.888 |
| MCS | 2.38 (-1.69-9.00) | 2.13 (-2.50-7.13) | 0.900 | 0.900 |  | 2.13 (-1.88-9.00) | 2.88 (-2.25-4.06) | 0.826 | 0.888 |
| rs1190605, no. (%) | 18 (40.00) | 27 (60.00) |  |  |  | 42 (93.33) | 3 (6.67) |  |  |
| Total score | 2.66 (-1.50-6.88) | 5.00 (0.44-10.06) | 0.215 | 0.380 |  | 3.78 (-0.31-9.75) | 0.44 (-3.13-40.44) | 0.856 | 0.964 |
| Physical function | 0 (-10.00-5.00) | 5.00 (0-20.00) | 0.048 | 0.194 |  | 0 (-5.00-15.00) | 20.00 (5.00-20.00) | 0.176 | 0.964 |
| Role-physical | 0 (0-0) | 0 (0-0) | 0.861 | 0.861 |  | 0 (0-0) | 0 (0-0) | 0.433 | 0.964 |
| Bodily pain | 0 (0-12.00) | 11.00 (0-26.00) | 0.050 | 0.194 |  | 6.50 (0-20.00) | 10.00 (-26.00-52.00) | 0.963 | 0.964 |
| General health | 0 (-5.00-5.00) | 0 (-5.00-20.00) | 0.494 | 0.604 |  | 0 (-5.00-15.00) | 0 (-10.00-20.00) | 0.908 | 0.964 |
| Vitality | 0 (-10.00-0) | 0 (-5.00-15.00) | 0.053 | 0.194 |  | 0 (-5.00-10.00) | 5.00 (-5.00-15.00) | 0.549 | 0.964 |
| Social function | 0 (0-12.50) | 12.50 (0-12.50) | 0.245 | 0.380 |  | 0 (0-12.50) | 0 (-12.50-12.50) | 0.475 | 0.964 |
| Role-emotional | 0 (0-0) | 0 (0-0) | 0.836 | 0.861 |  | 0 (0-0) | 0 (0-100.00) | 0.398 | 0.964 |
| Mental health | 0 (-8.00-0) | 0 (-4.00-8.00) | 0.092 | 0.253 |  | 0 (-4.00-8.00) | -4.00 (-4.00-4.00) | 0.563 | 0.964 |
| PCS | 1.63 (-2.50-12.50) | 8.00 (2.25-12.00) | 0.186 | 0.380 |  | 5.00 (0-12.00) | 3.75 (-4.00-48.00) | 0.964 | 0.964 |
| MCS | 0.50 (-4.50-6.38) | 3.13 (-0.25-10.13) | 0.276 | 0.380 |  | 2.38 (-1.50-9.00) | -2.25 (-2.88-32.88) | 0.767 | 0.964 |
| rs1190606, no. (%) | 19 (42.22) | 26 (57.78) |  |  |  | 38 (84.44) | 7 (15.56) |  |  |
| Total score | 2.13 (-1.50-6.88) | 5.00 (0.44-10.06) | 0.164 | 0.246 |  | 4.53 (-0.63-9.75) | 2.94 (0.44-11.06) | 0.888 | 0.983 |
| Physical function | 0 (-10.00-5.00) | 5.00 (0-20.00) | 0.093 | 0.207 |  | 2.50 (-5.00-15.00) | 5.00 (-5.00-20.00) | 0.788 | 0.983 |
| Role-physical | 0 (0-0) | 0 (0-0) | 0.788 | 0.788 |  | 0 (0-0) | 0 (0-0) | 0.983 | 0.983 |
| Bodily pain | 0 (0-16.00) | 10.50 (0-23.00) | 0.097 | 0.207 |  | 2.00 (0-20.00) | 10.00 (0-33.00) | 0.471 | 0.983 |
| General health | 0 (-5.00-10.00) | 0 (-5.00-20.00) | 0.440 | 0.538 |  | 0 (-5.00-15.00) | 0 (-5.00-0) | 0.555 | 0.983 |
| Vitality | 0 (-10.00-0) | 2.50 (-5.00-15.00) | **0.041** | 0.207 |  | 0 (-5.00-5.00) | 5.00 (-5.00-15.00) | 0.391 | 0.983 |
| Social function | 0 (0-12.50) | 12.50 (0-12.50) | 0.113 | 0.207 |  | 0 (0-12.50) | 12.50 (-12.50-12.50) | 0.844 | 0.983 |
| Role-emotional | 0 (0-0) | 0 (0-0) | 0.764 | 0.788 |  | 0 (0-0) | 0 (0-0) | 0.949 | 0.983 |
| Mental health | 0 (-8.00-0) | 4.00 (-4.00-8.00) | **0.006** | 0.066 |  | 0 (-4.00-4.00) | 4.00 (-4.00-12.00) | 0.214 | 0.983 |
| PCS | 1.50 (-2.50-13.50) | 6.50 (2.50-12.00) | 0.179 | 0.246 |  | 6.50 (0-12.50) | 3.25 (-2.50-12.00) | 0.707 | 0.983 |
| MCS | 0 (-4.75-4.38) | 3.25 (-0.25-10.13) | 0.113 | 0.207 |  | 2.06 (-1.88-9.00) | 3.38 (-2.25-10.13) | 0.594 | 0.983 |
| rs12161908, no. (%) | 42 (93.33) | 3 (6.67) |  |  |  | 45 (100.00) | 0 (0.00) |  |  |
| Total score | 3.34 (-0.31-9.81) | 6.06 (-4.44-9.75) | 0.946 | 0.946 |  | – | – | – | – |
| Physical function | 2.50 (-5.00-20.00) | 5.00 (0-15.00) | 0.731 | 0.804 |  | – | – | – | – |
| Role-physical | 0 (0-0) | 0 (0-0) | 0.594 | 0.804 |  | – | – | – | – |
| Bodily pain | 6.50 (0-20.00) | 16.00 (0-28.00) | 0.514 | 0.804 |  | – | – | – | – |
| General health | 0 (-5.00-10.00) | 15.00 (0-35.00) | 0.514 | 0.804 |  | – | – | – | – |
| Vitality | 0 (-5.00-10.00) | 0 (-15.00-0) | 0.333 | 0.804 |  | – | – | – | – |
| Social function | 0 (0-12.50) | 0 (-12.50-12.50) | 0.475 | 0.804 |  | – | – | – | – |
| Role-emotional | 0 (0-0) | 0 (0-0) | 0.595 | 0.804 |  | – | – | – | – |
| Mental health | 0 (-4.00-8.00) | 0 (-8.00-0) | 0.445 | 0.804 |  | – | – | – | – |
| PCS | 4.63 (-0.25-12.00) | 9.00 (0-19.50) | 0.682 | 0.804 |  | – | – | – | – |
| MCS | 2.38 (-1.88-9.00) | 0 (-8.88-3.13) | 0.306 | 0.804 |  | – | – | – | – |
| rs3818188, no. (%) | 14 (31.11) | 31 (68.89) |  |  |  | 41 (91.11) | 4 (8.89) |  |  |
| Total score | 0.66 (-0.63-6.88) | 5.00 (-0.19-9.81) | 0.404 | 0.902 |  | 5.00 (-0.19-9.81) | 0.59 (-14.58-2.66) | 0.090 | 0.194 |
| Physical function | 7.50 (0-20.00) | 0 (-5.00-15.00) | 0.186 | 0.682 |  | 5.00 (0-20.00) | -5.00 (-27.50-0) | 0.084 | 0.194 |
| Role-physical | 0 (0-0) | 0 (0-0) | 0.960 | 1.000 |  | 0 (0-0) | 0 (-37.50-0) | 0.105 | 0.194 |
| Bodily pain | 0 (0-10.00) | 12.00 (0-23.00) | 0.073 | 0.682 |  | 9.00 (0-20.00) | 10.00 (2.00-19.00) | 0.713 | 0.784 |
| General health | 0 (-5.00-10.00) | 0 (-5.00-15.00) | 0.492 | 0.902 |  | 0 (-5.00-15.00) | 2.50 (-2.50-10.00) | 0.792 | 0.790 |
| Vitality | 0 (-10.00-10.00) | 0 (-5.00-10.00) | 0.872 | 1.000 |  | 0 (-5.00-10.00) | -7.50 (-22.50-2.50) | 0.163 | 0.232 |
| Social function | 0 (-12.50-12.50) | 12.50 (0-12.50) | 0.162 | 0.682 |  | 0 (0-12.50) | -6.25 (-12.50-6.25) | 0.169 | 0.232 |
| Role-emotional | 0 (0-0) | 0 (0-0) | 0.906 | 1.000 |  | 0 (0-0) | 0 (0-0) | 0.106 | 0.194 |
| Mental health | 0 (-4.00-8.00) | 0 (-4.00-4.00) | 1.000 | 1.000 |  | 0 (-4.00-8.00) | -4.00 (-14.00--2.00) | 0.077 | 0.194 |
| PCS | 3.13 (-0.25-10.00) | 8.00 (0-12.50) | 0.632 | 0.993 |  | 5.00 (0-12.50) | 2.13 (-15.75-6.63) | 0.281 | 0.343 |
| MCS | 1.00 (-2.88-4.38) | 2.63 (-1.25-9.00) | 0.477 | 0.902 |  | 3.13 (-1.25-9.00) | -3.31 (-14.85-0.13) | 0.056 | 0.194 |
| *BH*, Benjamini-Hochberg method based on the false discovery rate | | | | | | | | | |

| **Table S27 Association between DYNC1H1 polymorphisms and anxiety in SLE patients** | | | | | | | | | | | |
| --- | --- | --- | --- | --- | --- | --- | --- | --- | --- | --- | --- |
| **Polymorphisms** | **Dominant model** | | | | |  | **Recessive model** | | | | |
|  | **Crude *OR* (*95% CI*)** | **Crude**  ***P* value** | **Adjusted^*^**  ***OR* (*95% CI*)** | **Adjusted**  ***P* value** | ***P_BH_*** |  | **Crude *OR* (*95% CI*)** | **Crude**  ***P* value** | **Adjusted^*^**  ***OR* (*95% CI*)** | **Adjusted**  ***P* value** | ***P_BH_*** |
| rs1004903 | 1.009 (0.620-1.641) | 0.972 | 1.006 (0.601-1.684) | 0.983 | 0.983 |  | 0.945 (0.401-2.230) | 0.898 | 0.923 (0.371-2.292) | 0.862 | 0.944 |
| rs11160668 | 1.210 (0.733-1.995) | 0.456 | 1.161 (0.683-1.974) | 0.581 | 0.983 |  | 4.006 (0.527-30.449) | 0.180 | 3.761 (0.472 -29.969) | 0.211 | 0.944 |
| rs1190605 | 0.823 (0.506-1.336) | 0.430 | 0.818 (0.487-1.376) | 0.449 | 0.983 |  | 0.895 (0.414-1.933) | 0.778 | 0.785 (0.346-1.784) | 0.564 | 0.944 |
| rs1190606 | 1.034 (0.627-1.703) | 0.897 | 1.108 (0.652-1.882) | 0.704 | 0.983 |  | 1.364 (0.644-2.889) | 0.417 | 1.234 (0.554-2.747) | 0.607 | 0.944 |
| rs12161908 | 0.843 (0.389-1.827) | 0.665 | 0.741 (0.323-1.698) | 0.478 | 0.983 |  | − | − | − | − | − |
| rs3818188 | 0.923 (0.529-1.608) | 0.776 | 1.046 (0.581-1.885) | 0.880 | 0.983 |  | 0.991 (0.562-1.749) | 0.976 | 1.022 (0.555-1.884) | 0.944 | 0.944 |
| *OR*, odds ratio; *CI*, confidence interval; *BH*, Benjamini-Hochberg method based on the false discovery rate;  **^*^**Adjusted for age, height, weight, sex, marital status, smoking, drinking, tea consumption, history of GCs therapy, GCs dose, and SLEDAI scores at baseline | | | | | | | | | | | |

| **Table S28 Association between DYNC1H1 polymorphisms and depression in SLE patients** | | | | | | | | | | | |
| --- | --- | --- | --- | --- | --- | --- | --- | --- | --- | --- | --- |
| **Polymorphisms** | **Dominant model** | | | | |  | **Recessive model** | | | | |
|  | **Crude *OR* (*95% CI*)** | **Crude**  ***P* value** | **Adjusted^*^**  ***OR* (*95% CI*)** | **Adjusted**  ***P* value** | ***P_BH_*** |  | **Crude *OR* (*95% CI*)** | **Crude**  ***P* value** | **Adjusted^*^**  ***OR* (*95% CI*)** | **Adjusted**  ***P* value** | ***P_BH_*** |
| rs1004903 | 1.034 (0.625-1.709) | 0.897 | 1.056 (0.623-1.789) | 0.840 | 0.840 |  | 1.045 (0.421-2.594) | 0.925 | 1.227 (0.469-3.205) | 0.677 | 0.943 |
| rs11160668 | 0.909 (0.548-1.506) | 0.710 | 0.856(0.502-1.461) | 0.568 | 0.840 |  | 1.043 (0.296-3.674) | 0.948 | 0.952 (0.249-3.640) | 0.943 | 0.943 |
| rs1190605 | 1.049 (0.637-1.726) | 0.852 | 1.065 (0.628-1.805) | 0.816 | 0.840 |  | 0.946 (0.423-2.117) | 0.893 | 0.967 (0.413-2.262) | 0.938 | 0.943 |
| rs1190606 | 1.051 (0.629-1.757) | 0.850 | 1.155 (0.673-1.983) | 0.601 | 0.840 |  | 1.063 (0.514-2.198) | 0.868 | 1.090 (0.502-2.365) | 0.828 | 0.943 |
| rs12161908 | 1.067 (0.457-2.490) | 0.882 | 0.879 (0.361-2.142) | 0.776 | 0.840 |  | − | − | − | − | − |
| rs3818188 | 1.136 (0.652-1.980) | 0.652 | 1.204 (0.670-2.166) | 0.535 | 0.840 |  | 1.145 (0.628-2.090) | 0.658 | 1.154 (0.610-2.184) | 0.660 | 0.943 |
| *OR*, odds ratio; *CI*, confidence interval; *BH*, Benjamini-Hochberg method based on the false discovery rate;  **^*^**Adjusted for age, height, weight, sex, marital status, smoking, drinking, tea consumption, history of GCs therapy, GCs dose, and SLEDAI scores at baseline | | | | | | | | | | | |

| **Table S29 Association between DYNC1H1 polymorphisms and HQROL in baseline patients** | | | | | | | | | | | |
| --- | --- | --- | --- | --- | --- | --- | --- | --- | --- | --- | --- |
| **Polymorphisms** | **Dominant model** | | | | |  | **Recessive model** | | | | |
|  | **Crude *OR* (*95% CI*)** | **Crude**  ***P* value** | **Adjusted^*^**  ***OR* (*95% CI*)** | **Adjusted**  ***P* value** | ***P_BH_*** |  | **Crude *OR* (*95% CI*)** | **Crude**  ***P* value** | **Adjusted^*^**  ***OR* (*95% CI*)** | **Adjusted**  ***P* value** | ***P_BH_*** |
| rs1004903 | 1.064 (0.734-1.542) | 0.745 | 1.081 (0.733-1.594) | 0.694 | 0.694 |  | 0.896 (0.461-1.742) | 0.746 | 0.970 (0.481-1.957) | 0.932 | 0.932 |
| rs11160668 | 1.233 (0.846-1.797) | 0.275 | 1.207 (0.815-1.786) | 0.348 | 0.418 |  | 1.762 (0.681-4.561) | 0.243 | 1.741 (0.651-4.655) | 0.269 | 0.666 |
| rs1190605 | 1.225 (0.847-1.772) | 0.280 | 1.230 (0.835-1.813) | 0.295 | 0.418 |  | 1.159 (0.633-2.122) | 0.633 | 1.371 (0.724-2.596) | 0.333 | 0.666 |
| rs1190606 | 0.870 (0.593-1.274) | 0.473 | 0.812 (0.544-1.212) | 0.308 | 0.418 |  | 0.869 (0.512-1.476) | 0.603 | 0.886 (0.507-1.549) | 0.671 | 0.805 |
| rs12161908 | 1.575 (0.841-2.950) | 0.156 | 1.790 (0.925-3.463) | 0.084 | 0.418 |  | 2.018 (0.182-22.408) | 0.568 | 2.123 (0.183-24.625) | 0.547 | 0.805 |
| rs3818188 | 0.810 (0.532-1.232) | 0.324 | 0.758 (0.488-1.175) | 0.215 | 0.418 |  | 0.629 (0.406-0.976) | 0.039 | 0.618 (0.388-0.983) | **0.042** | 0.252 |
| *OR*, odds ratio; *CI*, confidence interval; *BH*, Benjamini-Hochberg method based on the false discovery rate;  **^*^**Adjusted for age, height, weight, sex, marital status, smoking, drinking, tea consumption, history of GCs therapy, GCs dose, and SLEDAI scores at baseline | | | | | | | | | | | |
